# Detector‐specific correction factors for small‐field photon dosimetry in magnetic resonance‐guided radiation therapy: A systematic review and meta‐analysis

**DOI:** 10.1002/mp.70201

**Published:** 2026-01-07

**Authors:** Muhammed Emin Bedir, Ahtesham Ullah Khan, Wesley Culberson

**Affiliations:** ^1^ Vocational School of Technical Sciences Department of Electric and Energy Karamanoglu Mehmetbey University Karaman Türkiye; ^2^ School of Medicine and Public Health Department of Medical Physics University of Wisconsin‐Madison Madison Wisconsin USA

**Keywords:** detector response, detector‐specific correction factors, magnetic resonance‐guided radiation therapy (MRgRT), meta‐analysis, small‐field dosimetry

## Abstract

**Background:**

Accurate small‐field (SF) dosimetry is essential for Magnetic Resonance‐guided Radiation Therapy (MRgRT), particularly for stereotactic treatments. The static magnetic field (B‐field) alters detector response, necessitating detector‐specific correction factors $k_{B,Q}^{f,f}$ to ensure dosimetric accuracy. While numerous studies have reported such factors, the published data shows considerable variability, creating uncertainty for clinical practice.

**Purpose:**

This study was undertaken to (1) systematically review the literature on SF photon dosimetry in MRgRT and (2) perform a meta‐analysis to pool quantitative data and investigate the sources of this variability.

**Methods:**

A systematic search of four databases was conducted following PRISMA 2020 guidelines. A qualitative synthesis was performed on all included studies (*n* = 86). A random‐effects meta‐analysis was performed on studies providing correction factors (beam quality correction factor, *k*_msr_; field output correction factor, *k*_clin_) or uncorrected output factors (FOF) for fields ≤ 4 × 4 cm^2^ with associated uncertainties (*n *= 13 studies, 441 data points). Heterogeneity (*I* ^2^) was quantified, and subgroup analyses (B‐field, detector model, and orientation) and meta‐regression (field size) were used to investigate its sources.

**Results:**

The qualitative review identified key themes in foundational physics, detector‐specific characteristics, and clinical quality assurance. The meta‐analysis revealed extremely high heterogeneity (*I^2^* > 92%) across all pooled data, confirming that a single global‐average correction factor is statistically inappropriate. Subgroup analyses demonstrated that this heterogeneity is systematically driven by three main factors: magnetic field strength (e.g., 0.35 T vs. 1.5 T), specific detector model (with significant variations observed even among models of the same type), and detector orientation relative to the beam and B‐field. Meta‐regression showed that correction factors *k*_msr_ and *k*_clin_ demonstrated no statistically significant dependence on field size in this SF regime, which suggests that the theoretical variation expected due to beam property changes is statistically masked by the high heterogeneity (*I^2 ^> 92*) observed in the literature; conversely, FOFs showed a strong, statistically significant field‐size dependence.

**Conclusion:**

The findings confirm that detector response in MRgRT is a complex function of the measurement setup. A single correction factor is insufficient for clinical use. Dosimetric accuracy in MRgRT requires the use of correction factors that are specific to the MR‐Linac's B‐field strength, the exact detector model, and the measurement orientation. This analysis provides pooled, stratified data to support clinical physicists and inform the development of future dosimetry protocols.

## 1 INTRODUCTION

Magnetic resonance‐guided radiation therapy (MRgRT) represents a critical technology in the field of radiation oncology. The integration of magnetic resonance imaging (MRI) with a linear accelerator (MR‐Linac) provides superior soft‐tissue contrast for target and organ‐at‐risk (OAR) visualization. This unique capability enables real‐time motion management and online adaptive radiotherapy (ART), a process already being leveraged to tailor daily treatments for challenging sites such as thoracic and abdominal cancers.^1^, ^2^ This allows for the reduction of planning margins and facilitates dose escalation, which is particularly beneficial for stereotactic body radiotherapy (SBRT) and stereotactic radiosurgery (SRS) that rely on small, highly conformal radiation field. s.^3^, ^4^ However, the clinical advantages of MRgRT are predicated on the ability to deliver these complex treatments with a high degree of dosimetric accuracy.

The presence of a strong, static magnetic field introduces fundamental physics challenges that are absent in conventional radiotherapy. The Lorentz force, acting on secondary electrons produced by photon interactions, alters their trajectories. This perturbation manifests in system‐specific dosimetric effects. In perpendicular MR‐Linacs (e.g., Elekta Unity, ViewRay MRIdian), where the magnetic field is transverse to the beam direction, the most prominent phenomenon is the Electron Return Effect (ERE). At tissue–air or tissue–lung interfaces, electrons that would normally leave the volume are forced to curve back, causing a significant dose enhancement at the exit surface—an effect that has been the subject of intense experimental and computational investigation.^5^, ^6^ Conversely, in inline MR‐Linacs, where the magnetic field is parallel to the beam, the field acts as a magnetic lens, focusing contaminant electrons from the accelerator head onto the patient's skin and causing a substantial increase in entrance dose.^7^, ^8^, ^9^

Driven by advances in early cancer detection and intervention leading to treatment of smaller lesions, these physical complexities are compounded in the small‐field (SF) regime (typically defined as fields ≤ 4 × 4 cm^2^) used for stereotactic body radiation therapy (SBRT). In modern radiotherapy, such as SBRT, small fields present unique dosimetric challenges. A field is considered “small” when its dimensions are on the order of the lateral range of secondary electrons, leading to a loss of charged particle equilibrium. While no single geometric size serves as a rigid definition, in clinical practice, this regime is typically considered for fields with dimensions of 4 × 4 cm^2^ or smaller.^10^ This threshold serves as the operational upper bound for this review, and the term “small‐field” is used inclusively to describe the entire dosimetric range from this bound down to the “very small” fields (e.g., ≈ 5 mm) used for stereotactic procedures. SF dosimetry is already challenging due to the loss of lateral charged particle equilibrium, partial source occlusion, and the influence of detector volume averaging.^11^ The superposition of magnetic field effects further perturbs the radiation field and can significantly alter the response of dosimetry detectors. This has necessitated a complete re‐evaluation of quality assurance (QA) protocols, from assessing the impact of geometric distortions on MRI‐based planning to developing novel end‐to‐end testing procedures using specialized anthropomorphic phantoms.^12^, ^13^, ^14^ The magnitude and direction of a detector's response alteration depend on a complex interplay of factors, including its construction materials, its sensitive volume geometry, and its orientation relative to the magnetic field and radiation beam.^15^, ^16^ Consequently, standard dosimetry protocols, such as *IAEA TRS‐483*, are not directly applicable, and detector‐specific correction and small field output factors, denoted as $k_{B,Q}^{f,f}$, are required to convert a detector's reading in the magnetic field to an accurate dose measurement.

Over the past several years, numerous individual studies have been dedicated to characterizing this new dosimetric landscape. Researchers have employed a wide array of techniques, including detailed Monte Carlo (MC) simulations to model accelerator heads and detector responses^17^, ^18^ and extensive experimental measurements to determine correction factors for a variety of detectors.^11^, ^16^ While these studies have been instrumental, they also reveal a landscape of varying experimental setups, detector choices, and simulation parameters. This has resulted in a collection of detector‐specific correction factors that, while valuable, lack the consolidated authority of a pooled analysis.

While a qualitative systematic review is crucial for identifying this landscape (our first objective), it cannot, by itself, produce the single, statistically robust “consensus values” that the clinical community requires. A meta‐analysis, in contrast, offers a powerful quantitative method to pool these disparate data points, thereby increasing statistical power and yielding a more precise, consolidated estimate.^19^ Crucially, this quantitative synthesis also allows for a formal investigation of heterogeneity, enabling us to systematically dissect why reported values differ.^19^ The absence of such a pooled, quantitative analysis presents a significant challenge in MRgRT. The commissioning of MR‐Linac systems is rendered a complex, “custom task” because the magnetic field interacts with the radiation beam and alters detector response.^7^ This effect is not uniform; the resulting measurements show significant variability depending on the specific detector model used, with different values reported for various ionization chambers and solid‐state detectors.^20^ This problem is especially pronounced in SF photon dosimetry, where literature on the topic remains “scarce” for some configurations.^21^ To account for these physical effects, formalisms have been proposed that require “detector type specific output correction factors”,^22^ often denoted as $k_{B,Q}^{f,f}$.^20^, ^22^ However, the wide heterogeneity in reported values for these factors, driven by different detectors, magnetic field strengths, and measurement setups, and the lack of formal guidelines creates significant uncertainty for clinical physicists tasked with commissioning MR‐Linac systems.^7^ Therefore, this study was undertaken to (1) systematically review the published literature on SF photon dosimetry in MRgRT systems, identifying key themes and challenges, and (2) perform a focused meta‐analysis to calculate pooled, detector‐specific correction and small field output factors $k_{B,Q}^{f,f}$ to provide robust, evidence‐based guidance for the medical physics community.

## 2 MATERIALS AND METHODS

### 2.1 Protocol

This systematic review and meta‐analysis was conducted and reported in accordance with the Preferred Reporting Items for Systematic Reviews and Meta‐Analyses (PRISMA) 2020 statement.^23^ The complete PRISMA 2020 checklist is provided in (Table S1).

### 2.2 Eligibility criteria

Studies were selected with predefined eligibility criteria, which are detailed in (Table 1).

**TABLE 1:** Eligibility criteria for study selection.

Criterion	Description
**1.1**	**Reports a Core Quantitative Outcome**: The study must explicitly report a numerical value for a detector‐specific correction factor or a SF output factor (OF) measured under MR‐Linac conditions.
**1.2**	**Includes Uncertainty**: The reported quantitative outcome must be accompanied by its associated statistical uncertainty (SD, SE, or CI).
**1.3**	**Provides Sufficient Methodological Context**: The study must identify the specific detector model, MR‐Linac system, field size(s), and detector orientation.
**2.1**	**Comparative Analysis**: The study compares different detectors, techniques, or TPS algorithms.
**2.2**	**Methodological Guidance**: The study discusses challenges, solutions, or best practices in MRgRT dosimetry.
**2.3**	**Performance Validation**: The study validates a system or method using metrics like gamma analysis or percent difference.
**2.4**	**Foundational Physics**: The study explains the underlying physics of detector response in a magnetic field.
**2.5**	**Future Directions**: The study identifies gaps in the literature or suggests future research.

*Note*: Studies meeting all criteria 1.1–1.3 for small fields (≤ 4 × 4 cm^2^) were included in the meta‐analysis (Group 1). Studies meeting any of criteria 2.1–2.5, or meeting Group 1 criteria for large fields only, were included in the qualitative synthesis (Group 2).

### 2.3 Information sources and search strategy

A comprehensive literature search was conducted across four electronic databases: Web of Science, Scopus, PubMed/MEDLINE, and Google Scholar. The search was limited to publications from 1 January2020to20July2025. This timeframe was specifically selected to ensure that the extracted dosimetric data (correction factors and output factors) primarily adhere to the formalisms established in the IAEA TRS‐483 code of practice (published in 2017). Excluding studies prior to this period minimizes the heterogeneity arising from the use of pre‐standardization protocols, ensuring that the pooled estimates in the meta‐analysis are methodologically consistent and clinically relevant to current standards. The search strategy combined keywords and subject headings related to three core concepts: (1) the MRgRT environment (e.g., “MR‐Linac”, “magnetic field”), (2) dosimetry concepts (e.g., “small field dosimetry”, “correction factors”, “detector response”), and (3) validation methods (e.g., “Monte Carlo”, “quality assurance”). The detailed search query for each database is provided in (Table S2), with a summary of search results before and after filtering. The filters applied are detailed in (Table S3).

### 2.4 Selection process

The study selection was performed in a two‐stage process. First, titles and abstracts of all identified records were screened for relevance against the eligibility criteria. A total of 320 unique articles were screened at this stage. The criterias for study selection are detailed in (Text A1). A full list of these 320 articles with screening decisions is provided in (Table S4). Subsequently, the full texts of all potentially relevant articles were retrieved and assessed for final inclusion. This resulted in 86 articles undergoing full‐text review.

### 2.5 Data collection and quality assessment

Data from the included studies were extracted into standardized forms. For the qualitative synthesis, information on study objectives, methodology, key findings, and conclusions was extracted. For the quantitative meta‐analysis, the following data were extracted: study identifier, detector model, MR‐Linac system and magnetic field strength, detector orientation, field size, output value formalism, output value, and its associated uncertainty (standard deviation or confidence interval). A quality and risk‐of‐bias assessment was performed for all included studies using a tool based on the eligibility criteria. The detailed results of this assessment are provided in (Text S2, Tables S5 and S6)

### 2.6 Data synthesis and statistical analysis

#### 2.6.1 Qualitatitive synthesis

A narrative synthesis of the findings from the qualitative review was conducted, with results grouped by thematic area.

#### 2.6.2 Quantitative synthesis (meta analysis)

For the quantitative synthesis, a meta‐analysis was performed using a random‐effects model to account for anticipated heterogeneity between studies. This meta‐analysis systematically synthesized data on three key dosimetric quantities defined within the International Atomic Energy Agency (IAEA) Technical Reports Series No. 483 (TRS‐483) formalism for SF dosimetry (International Atomic Energy Agency & American Association of Physicists in Medicine, 2017).^24^ The quantities investigated were the beam quality correction factor (*k*_msr_), which corrects the detector response from a standard calibration field to the machine‐specific reference (msr) field of the MR‐Linac; the field output correction factor (*k*_clin_), which corrects the ratio of detector readings to determine the true absorbed dose ratio between a small clinical field and the msr field; and the uncorrected field output factor (FOF), representing the raw ratio of detector readings between a clinical field and the msr field.

The quantitative meta‐analysis relies on the formalism for small fields in the presence of magnetic fields, adapting the nomenclature from IAEA TRS‐483. The absorbed dose to water at the reference depth in a magnetic field for a machine‐specific reference field $f_{msr}$ is given by:

$$D_{w,Q_{msr}}^{f_{msr}}=M_{Q_{msr}}^{f_{msr}}\cdot N_{D,w,Q_{0}}\cdot k_{Q_{msr}Q_{0}}^{f_{msr}f_{ref}}$$

where:

- $M_{Q_{msr}}^{f_{msr}}$ is is the corrected detector reading in the *msr* field.
- $N_{D,w,Q_{0}}$ is the calibration coefficient in terms of absorbed dose to water for the detector in a reference beam quality $Q_{0}$ (typically *^60^Co*).
- $k_{Q_{msr}Q_{0}}^{f_{msr}f_{ref}}$(denoted in this study as $k_{msr}$ is the beam quality correction factor that accounts for the differences between the reference conditions and the *msr* field in the magnetic field.

For small clinical fields $\left(f_{clin}\right)$, the absorbed dose is determined using the field output factor $(\Omega_{Q_{clin}Q_{msr}}^{f_{clin}f_{msr}}):$

$$D_{w,Q_{clin}}^{f_{clin}}=D_{w,Q_{msr}}^{f_{msr}}\cdot \Omega_{Q_{clin}Q_{msr}}^{f_{clin}f_{msr}}$$

The field output factor is formally defined as the product of the ratio of detector readings (uncorrected field output factor, FOF) and the detector‐specific output correction factor:

$${\;\Omega }_{Q_{clin}Q_{msr}}^{f_{clin}f_{msr}}=\left[\frac{M_{Q_{clin}}^{f_{clin}}}{M_{Q_{msr}}^{f_{msr}}}\right]\cdot k_{Q_{clin}Q_{msr}}^{f_{clin}f_{msr}}$$

where $k_{Q_{clin}Q_{msr}}^{f_{clin}f_{msr}}$ (denoted in this study as $k_{clin}$ is the field output correction factor that corrects the detector response for the difference between the clin and msr fields in the presence of the magnetic field.

Although the title emphasizes SF dosimetry, the scope definition for the quantitative meta‐analysis, which is strictly focused on fields (≤ 4 × 4 cm^2^), necessarily includes the beam quality correction factor $k_{\mathrm{msr}}$ within its analytical domain. This is because the overall dosimetric accuracy of any small field measurement is directly predicated on the accurate determination of $k_{\mathrm{msr}}$, which provides the magnetic field‐specific correction at the machine reference condition $f_{msr}$. Consequently, $k_{\mathrm{msr}}$ is analyzed not as an independent reference dosimetry topic, but as the foundational reference step required to achieve accurate dose determination for the small clinical fields, thereby ensuring the integrity of the subsequent *k*_clin_, and FOF values. The broader qualitative synthesis (comprising the remaining *n* = 73 studies) was designed to capture general methodological discussions and comparative analyses, including those studies focused on larger fields (>4 × 4 cm^2^) that did not meet the strict criteria for meta‐analysis.

A random‐effects meta‐analysis model, utilizing the Restricted Maximum Likelihood (REML) estimator, was employed to calculate the pooled mean estimates and their corresponding 95% confidence intervals (CIs) for each dosimetric quantity. A random‐effects model was deemed most appropriate due to the anticipated high degree of variability in experimental setups, detector models, and measurement conditions across the included studies. It is noted that this approach treats all 441 data points as independent entries. While many data points are “nested” within the 13 contributing studies, this method was chosen to maximize the granularity of the subgroup and meta‐regression analyses. The potential impact of this statistical assumption is addressed as a limitation in Section 4.

The degree of heterogeneity among studies was quantified using the *I* ^2^ statistic. An *I* ^2^ value greater than 75% was considered indicative of substantial heterogeneity, as per conventional guidelines,^25^ warranting further investigation into its sources. To dissect this heterogeneity, comprehensive subgroup analyses were performed on a series of pre‐defined categorical variables. These variables included: Detector Type (e.g., Ion Chamber, Diode), specific Detector Model (e.g., PTW 31016 PinPoint 3D), Magnetic Field Strength (B‐Field), MR‐Linac System (e.g., Elekta Unity, ViewRay MRIdian), the method of data generation (Experimental Measurements vs. MC simulations), and Detector Orientation relative to the radiation beam and magnetic field. For the continuous variable of SF size, a meta‐regression analysis was conducted to model its relationship with the three dosimetric quantities.

**FIGURE A1:**
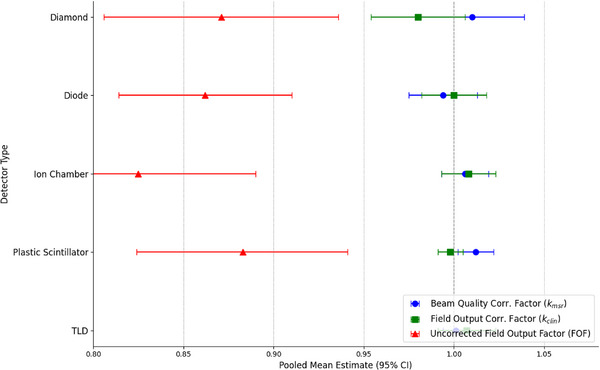
Forest plot comparing pooled mean estimates and 95% confidence intervals for beam quality correction factor (*k*_msr_), field output correction factor (*k*_clin_), and uncorrected field output factor (FOF) across different detector types (diamond, diode, ion chamber, plastic scintillator, TLD). The vertical dashed line indicates unity for correction factors. Subgroup heterogeneity (*I* ^2^) is reported for each detector type.

**FIGURE A2:**
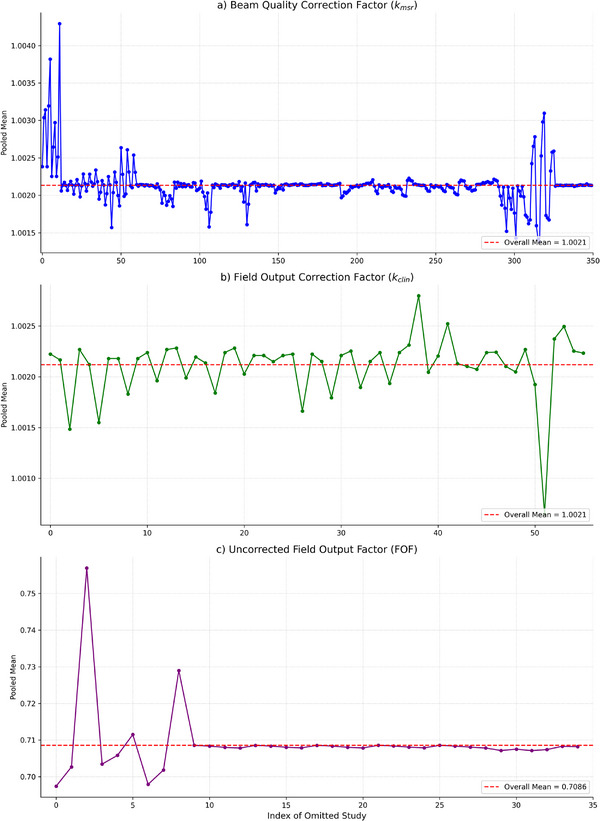
Publication bias. (a) Beam quality correction factor (*k*_msr_), (b) field output correction factor (*k*_clin_), and (c) uncorrected field output factor (FOF). The *y*‐axis represents the precision of each study (inverse of the standard error), while the *x*‐axis represents the measured output value. In the absence of publication bias, studies are expected to be distributed symmetrically around the pooled mean estimate (vertical solid line).

**FIGURE A3:**
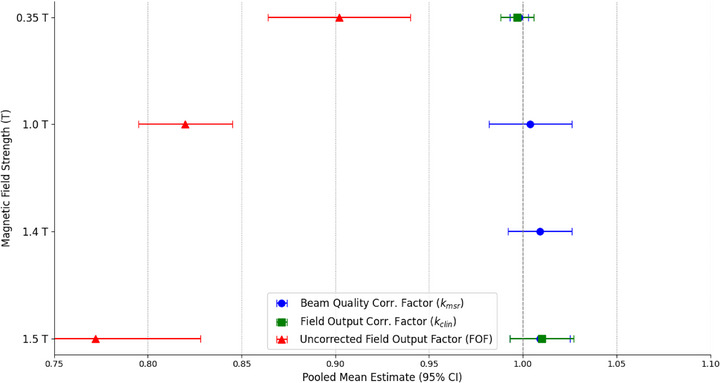
Forest plot illustrating the effect of magnetic field strength (0.35, 1.0, 1.4, and 1.5 T) on the pooled mean estimates and 95% confidence intervals for beam quality correction factor (*k*_msr_), field output correction factor (*k*_clin_), and uncorrected field output factor (FOF). Subgroup heterogeneity (*I* ^2^) is reported for each B‐field strength category.

To dissect this heterogeneity, comprehensive subgroup analyses were performed, such as the stratification by detector type detailed in (Figure A1). To ensure the robustness of our findings, a leave‐one‐out sensitivity analysis was performed. This iterative process involved removing one study at a time and recalculating the pooled mean estimate to confirm that no single study exerted a disproportionate influence on the overall results. The outcomes of this analysis are detailed in (Figure A2). Furthermore, the potential for publication bias was assessed through visual inspection of funnel plots, which are presented in (Figure A3). The calculation and mathmetical framework of meta‐analysis is explained in (Text S3).

## 3 RESULTS

### 3.1 Study selection

**FIGURE 1:**
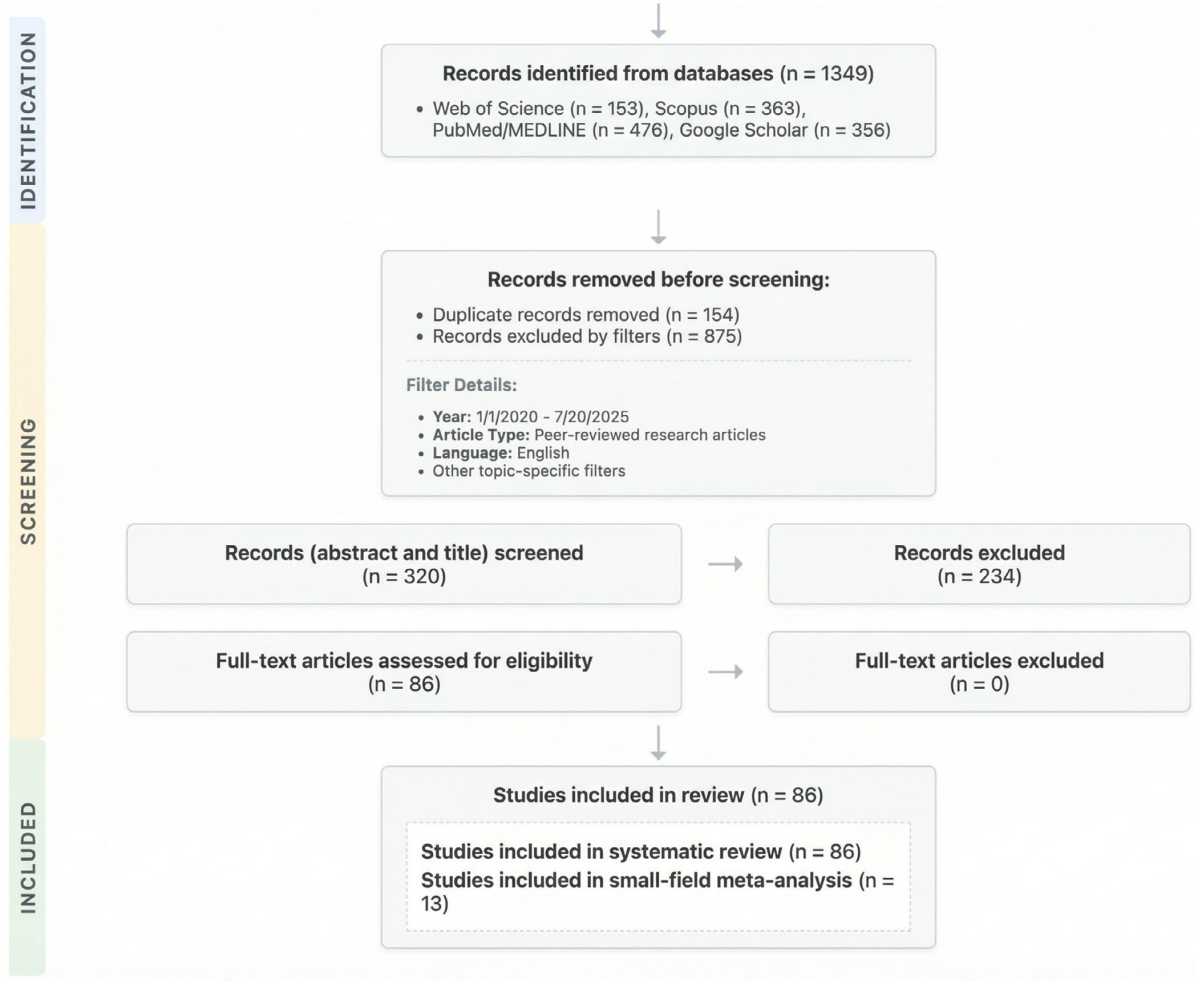
(PRISMA 2020 Flow Diagram): Figure illustrates the flow of information through the different phases of the systematic review. It maps out the number of records identified, included, and excluded, and the reasons for exclusions.

The initial database search yielded 474 records. After removing 154 duplicates, 320 unique records were screened, which led to the exclusion of 234 records based on title and abstract. The remaining 86 articles were assessed via full‐text review and all were included in the systematic review. The final inclusion/exclusion decision for each of these 86 articles, with detailed justifications against the criteria in (Table 1), is provided in (Table S7). Of the 86 studies, 13 provided 441 data points eligible for the SF meta‐analysis. The study selection process is detailed in the PRISMA flow diagram (Figure 1).

### 3.2 Study characteristics and quality assessment

The 86 included studies were published between 2020 and June 20, 2025, and investigated a range of MRgRT systems, including the 1.5 T Elekta Unity, the 0.35 T ViewRay MRIdian, and various research prototypes with both inline and perpendicular magnetic field orientations. A wide variety of detectors were characterized, including multiple models of ionization chambers, solid‐state diodes, diamond detectors, plastic scintillators, and optically stimulated luminescence dosimeters (OSLDs). The overall methodological quality of the included studies was high. Based on a five‐domain assessment, 76 of the 86 studies (88.4%) were rated as having a low risk of bias, with most providing detailed descriptions of their experimental or computational setups and a clear statement of uncertainties. Detailed results of the quality and risk‐of‐bias assessment are available in (Text S2).

### 3.3 Qualitative synthesis

The 73 studies included in the qualitative synthesis addressed a broad spectrum of challenges and innovations in MRgRT dosimetry. Our analysis identified four primary, often interconnected, themes that describe the field's focus: clinical implementation and quality assurance, foundational physics and interface dosimetry, the influence of detector‐specific characteristics, and the development of novel dosimetry systems. The key findings from each of the 73 studies are detailed in (Table S8). The progression of these themes illustrates a logical evolution of research: from identifying fundamental physics problems to understanding detector responses, and finally, to developing novel tools and clinical procedures to ensure safety and accuracy.

A significant body of research focused on characterizing the novel physics perturbations unique to the MRgRT environment. In perpendicular systems, studies consistently investigated the ERE at exit surfaces, with some using novel optical imaging techniques to reveal that its magnitude could differ from TPS predictions by up to 20%,^26^ while other work confirmed the accuracy of TPS calculations for ERE around air cavities.^6^ For inline systems, research focused on the entrance dose enhancement from magnetically focused contaminant electrons,^8^ and explored novel mitigation strategies, such as magnetic deflectors and helium‐filled volumes, to restore a skin‐sparing effect.^27^ The field continues to evolve, with recent work identifying and describing a previously unreported phenomenon, the “lateral scatter electron return effect” (LS‐ERE), which can cause asymmetric dose elevations outside the primary field in parallel‐orientation systems.^28^

Flowing directly from the observation of physical perturbations, many studies explored detector‐specific characteristics and why different detectors respond differently in a magnetic field. It was consistently found that a detector's response is a direct consequence of its physical construction. MC simulations provided deep physical insight, showing that the magnetic field significantly alters the electron fluence spectra within the detector itself.^15^ Key drivers of a detector's unique response were identified as its internal geometry, the density of the detector material, the number of density interfaces, and the presence of high‐density, high‐Z extracameral components.^16^

The unique challenges of the MRgRT environment have also spurred significant innovation in the development of novel dosimetry systems and methods. Research has focused on establishing new methods for traceable, absolute dosimetry using graphite calorimetry^29^ and water calorimetry.^30^ Regarding the latter, we include the foundational work of de Prez et al. (2019) to ensure technical completeness, acknowledging that this seminal study falls slightly outside our primary systematic search window. Furthermore, the use of alanine has been validated as a robust transfer standard.^31^ Beyond reference‐class dosimetry, significant effort has gone into characterizing and developing robust protocols for novel relative dosimetry systems, such as plastic scintillators,^32^ and characterizing the orientation and energy dependence of OSLDs.^33^

Finally, the largest body of research addressed the practical challenges of clinical implementation and QA. A critical finding replicated across multiple studies was the “air gap effect,” where small air gaps around detectors in solid phantoms cause substantial dose over‐responses (e.g., +13% for a 1 mm gap).^34^ This represents a major clinical safety risk, as an unaware physicist could incorrectly “adjust” machine output based on this erroneous reading, leading to a systematic underdosing of patients. This finding strongly reinforces the recommendation to use water phantoms for any reference‐class measurements and has spurred the development of specialized detectors designed to be immune to this effect.^35^

While this thematic analysis reveals the overarching research trends, a clinical physicist is often faced with practical questions about a specific detector they intend to use. To translate these broad themes into actionable guidance, the following sections synthesize the key findings from the literature as they pertain to each major detector class.

#### 3.3.1 Ionization chambers

Ionization chambers (ICs) remain the gold standard for reference dosimetry and were the most frequently investigated detector class. The primary finding is that air‐filled ICs require significant, orientation‐dependent correction factors. Studies consistently report that chambers oriented perpendicular to the magnetic field require larger corrections than those oriented parallel,^36^, ^37^ a conclusion supported by numerous experimental investigations^31^, ^38^, ^39^, ^40^, ^41^ and MC simulations.^42^ The specific chamber construction, including the volume and shape of the collecting electrode and guard rings, was shown to be a critical determinant of the final correction factor.^16^ For clinical QA, the “air gap effect” is a major concern for ICs, where measurements in solid phantoms can be severely distorted by localized ERE in surrounding air,^34^ necessitating the use of water phantoms or specially designed, gapless phantoms, and chambers.^35^

#### 3.3.2 Semiconductor detectors (diodes and diamond)

Semiconductor detectors are highly valued in MRgRT dosimetry for their high spatial resolution, which is critical for SF measurements. However, their response in magnetic fields requires careful characterization. Studies on silicon diodes confirmed that their response can be significantly affected by the magnetic field, showing dependencies on dose rate and beam energy that necessitate careful correction.^43^ In contrast, the PTW 60019 microDiamond detector has consistently emerged as a particularly robust and promising option for relative dosimetry. Its near‐tissue equivalence and high stability were highlighted in multiple investigations. For example, in one comprehensive detector assessment for a low‐field system, the microDiamond showed the best overall agreement for relative dosimetry tasks across a full range of field sizes.^44^ Other studies have validated its use for output factor measurements in high‐field systems, solidifying its role as a preferred detector for commissioning and routine QA.^11^ Another key semiconductor device, the high‐resolution MOSkin detector, was specifically validated as an ideal tool for characterizing the steep and complex dose gradients at patient surfaces, with these measurements providing benchmark data for ERE and entry skin dose.^45^ Finally, the application of semiconductor technology in larger array formats has led to significant workflow improvements, with the validation of diode array phantoms for time‐resolved, dynamic QA allowing for efficient verification of complex deliveries like gated treatments and sliding window IMRT.^46^

#### 3.3.3 Scintillation and OSLD detectors

Plastic scintillation detectors (PSDs) were consistently shown to be excellent candidates for MRgRT dosimetry due to their near‐water equivalence, which leads to very small intrinsic magnetic field dependencies.^47^ The primary challenge for PSDs is not the magnetic field itself, but the accurate removal of signal from Cherenkov light generated in the optical fiber (the “stem effect”), for which robust protocols and uncertainty analyses have been developed.^32^ The characterization of multi‐point scintillation systems has shown their promise for advanced applications,^48^ and inorganic scintillators are also being explored.^49^ Optically stimulated luminescence dosimeters (OSLDs) were frequently used for in vivo and surface dosimetry, including quantifying skin‐sparing^50^ and ERE.^51^ While their dose–response linearity is unaffected by the magnetic field, their angular dependence is significantly increased, requiring either a large, orientation‐specific correction factor or ensuring the OSLD is calibrated and used in the same orientation.^33^ OSLDs have also been used to assess out‐of‐field dose.^52^

#### 3.3.4 Radiochromic film

Radiochromic film was consistently validated across numerous studies as a powerful and indispensable tool for high‐resolution, two‐dimensional dosimetry in MR‐Linacs. Its most significant advantage, confirmed repeatedly, is that its dose response is almost entirely independent of the magnetic field's presence, strength, and orientation.^53^ This unique property makes it an invaluable and trusted standard for validating the accuracy of TPS dose calculations. Because of its high fidelity, film has been the preferred dosimeter for experimentally verifying complex physical phenomena like the ERE at tissue‐air interfaces.^6^ Its utility is especially pronounced in the commissioning of high‐precision treatments like SRS and SBRT, where film is used to measure and verify small field penumbra and shapes.^54^ However, these studies also highlight the importance of careful protocol, such as performing measurements in water or adding water around the film to minimize air gaps, which can otherwise introduce a localized ERE and corrupt the measurement.^54^ Film has also been central to end‐to‐end workflow validation using complex, deformable phantoms,^13^ reaffirming its status as a reliable, high‐resolution dosimeter for MRgRT QA.

#### 3.3.5 Clinical workflow, commissioning, and advanced modeling studies

A vast number of studies focused on the broader clinical workflow and the computational tools required for safe and effective MRgRT. This includes detailed commissioning reports from new MR‐Linac centers,^55^, ^56^ and the development of novel end‐to‐end QA phantoms.^14^, ^57^ Many studies demonstrated the clinical feasibility and dosimetric advantages of MRgRT for various sites, including the brain,^3^, ^58^ head and neck,^59^ gynecological cancers,^1^ and pancreas.^2^ The ability to perform online adaptation was shown to be critical for improving target coverage and sparing organs at risk,^60^, ^61^ though the dosimetric impact of intrafraction motion remains a challenge.^62^ The entire adaptive workflow has been experimentally validated,^63^ and specific techniques like respiratory gating^64^ and an intrafraction drift motion correction^65^ have been assessed. The management of motion for sites like the larynx has also been quantified.^66^ In addition to technical validation, clinical processes were also a focus, with studies validating practices like online contouring by trained radiographers to improve workflow efficiency.^67^

To ensure safety, the development and integration of independent dose verification software was a key theme,^68^, ^69^, ^70^ alongside platforms to automate and streamline the online QA process.^71^ The foundational physics of MRgRT was explored in studies characterizing the ERE with novel optical methods, investigating a new “Lateral Scatter ERE^28^”, and modeling dose perturbations from gas cavities.^72^ Others focused on characterizing out‐of‐field dose and the electron streaming effect.^73^ The fundamental interactions within detectors were also explored in detail.^15^ Finally, a major area of innovation is in advanced computational modeling. This includes the development of vendor‐independent MC models of accelerator heads,^17^, ^74^ new MC subroutines for complex geometries,^75^ and novel dose calculation algorithms.^76^ There is a strong focus on MRI‐only workflows, with numerous studies validating deep learning‐based synthetic CT (sCT) generation for both photon and proton.^12^, ^77^, ^78^, ^79^, ^80^, ^81^, ^82^, ^83^ The field is moving toward real‐time dose estimation^84^ and even AI‐based dose calculation engines that completely bypass the need for an sCT.^85^, ^86^ This large body of work, validated with various dosimeters, underpins the ongoing effort to make MRgRT safer, faster, and more accurate.

### 3.4 Quantitative synthesis (small‐field meta‐analysis)

Thirteen studies provided 441 data points that met the criteria for inclusion in the meta‐ analysis. The detailed characteristics and extracted data for each of these 441 data points are presented in (Table S9).

#### 3.4.1 Overall pooled estimates and pervasive heterogeneity

The initial meta‐analysis, pooling all 441 extracted data points, was first performed to quantify the overall level of heterogeneity. While this pooling yielded global average estimates (eg., *k*_msr_ = 1.006[95%CI:0.993,1.019]; *k*_clin_ = 1.008[95%CI:0.993,1.023]; and FOF = 0.871[95%CI:0.838,0.904], as detailed in (Table S10), it must be emphasized that these values are not clinically substantiated and should not be used.

A critical finding from this initial analysis was the extremely high level of heterogeneity across all three groups (*I* ^2^ > 92% for all). These *I* ^2^ values indicate that the global pooled means are statistically inappropriate, as they misleadingly average data from fundamentally different underlying experimental conditions (e.g., different B‐fields, detector models, and orientations). This pervasive heterogeneity confirms that a single global average is insufficient to describe the complex dosimetric landscape of MR‐Linacs and provides a strong mandate for the detailed subgroup analyses that follow.

#### 3.4.2 Impact of detector type and model

The choice of radiation detector is a critical factor in small field dosimetry, and its importance is amplified in the presence of a magnetic field. When stratified by broad detector categories, as illustrated in (Figure A1) and quantified in (Table S11), different detector types exhibit distinct behaviors, particularly for the uncorrected FOF. Ion chambers demonstrate the lowest pooled FOF of 0.825[95%CI:0.760,0.890], whereas plastic scintillators show the highest FOF of 0.883[95%CI:0.824,0.941]. This discrepancy is a clear manifestation of the detector volume averaging effect, where a detector's finite size leads to an underestimation of the dose in a steep dose gradient.^87^

**FIGURE 2:**
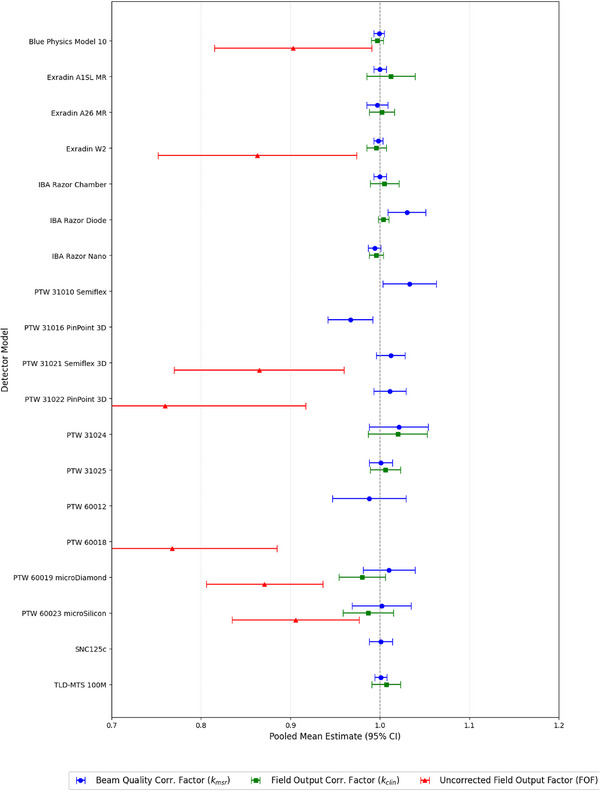
Forest plot detailing pooled mean estimates and 95% confidence intervals for *k*_msr_, *k*_clin_, and FOF for specific detector models. Subgroup heterogeneity (*I* ^2^) is provided for each model where sufficient data exists.

Drilling down to the level of specific detector models, as shown in (Figure 2) (with detailed data in (Tables S12, S13, and S14), reveals significant performance variability that is masked by the broader type‐based analysis. This demonstrates that a “one‐size‐fits‐all” approach to correction factors for a given detector type is inadequate for high‐accuracy dosimetry. For instance, within the diode category, the IBA Razor Diode has a pooled *k*_msr_ of 1.030[95%CI:1.010,1.051], while the PTW 60012 diode has a value of 0.988[95%CI:0.947,1.029]. Even more striking variations are observed among ion chambers: the PTW 31010 Semiflex shows a large correction factor of 1.033[95%CI:1.003,1.063], whereas the PTW 31016 PinPoint 3D exhibits a value significantly below unity at 0.967[95%CI:0.942,0.992]. These model‐specific differences arise from unique interactions between the detector's internal geometry, construction materials, and the external magnetic field, which collectively influence charge collection efficiency.^88^ This finding underscores the necessity of using model‐specific, rather than type‐specific, correction factors.

#### 3.4.3 Sensitivity and bias assesment

To ensure the overall conclusions of the meta‐analysis were not disproportionately influenced by any single publication, a leave‐one‐out sensitivity analysis was performed. This involved systematically removing each publication one at a time and recalculating the pooled estimate for the remaining studies.

**FIGURE 3:**
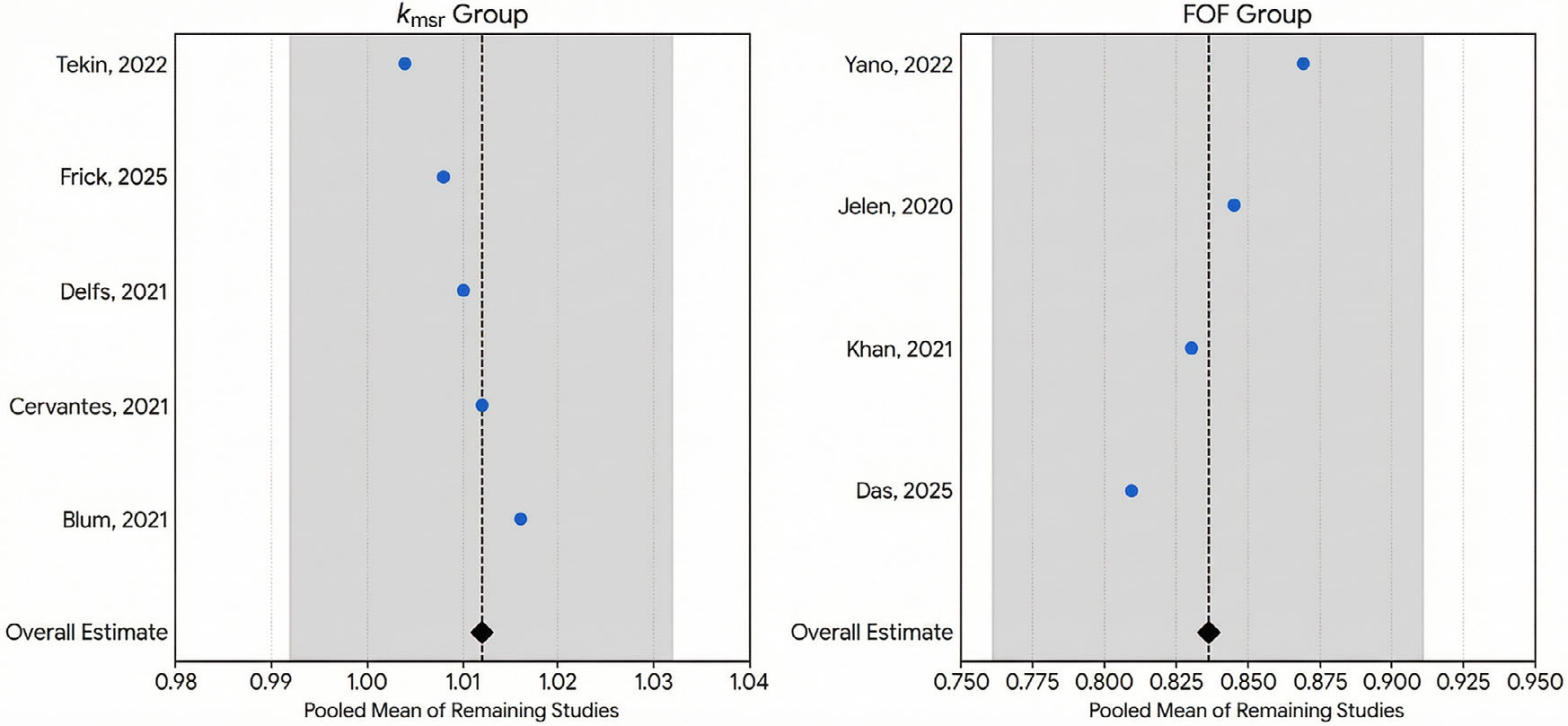
Leave‐one‐out sensitivity analysis: This analysis evaluates the robustness of the pooled estimates for the *k*_msr_ (left panel) and FOF (right panel) groups. Each circle represents the recalculated pooled mean after omitting the corresponding study listed on the *y*‐axis. The vertical dashed line and black diamond indicate the overall pooled mean. The grey shaded area represents the 95% confidence interval (CI) of the overall estimate. For both groups, all recalculated means fall well within the overall CI, confirming that the meta‐analysis results are robust and not unduly influenced by any single publication.

The results, presented in (Figure 3), demonstrate the robustness of our findings for the *k*_msr_ and FOF groups. For the *k*_msr_ group, the overall pooled mean was 1.012 (95% CI: 0.992–1.032). When individual studies were omitted, the recalculated pooled means remained stable, ranging from a minimum of 1.004 (when omitting^89^) to a maximum of 1.016 (when omitting^11^). Similarly, for the FOF group, the overall pooled mean was 0.836 (95% CI: 0.761–0.911). The recalculated means varied only slightly, from 0.809 (when omitting^90^) to 0.869 (when omitting^91^).

For both groups, all recalculated estimates remained well within the confidence interval of the original overall estimate. This indicates that the conclusions drawn are a true synthesis of the available literature and are not skewed by any single influential publication. It should be noted that this analysis was not performed for the *k*_clin_ group, as it consisted of only two publications, which is insufficient for a meaningful sensitivity assessment.

In addition to the leave‐one‐out analysis, the potential for publication bias was thoroughly assessed. For this, funnel plots were generated for each of the three dosimetric quantities, as presented in (Figure A2). This graphical method plots the effect size of each study against its precision (standard error). In the absence of bias, the distribution of studies should be symmetrical and resemble an inverted funnel, as smaller, less precise studies are expected to scatter more widely at the bottom, while larger, high‐precision studies cluster tightly at the top. Upon visual inspection of the plots, the studies for *k*_msr_, *k*_clin_, and FOF were observed to be distributed in a generally symmetric pattern around the overall pooled mean estimate. This symmetry indicates that there is no apparent relationship between study size and effect size, suggesting that a significant publication bias is unlikely to have influenced the results of this meta‐analysis.

#### 3.4.4 The defining influence of the magnetic field

**FIGURE A4:**
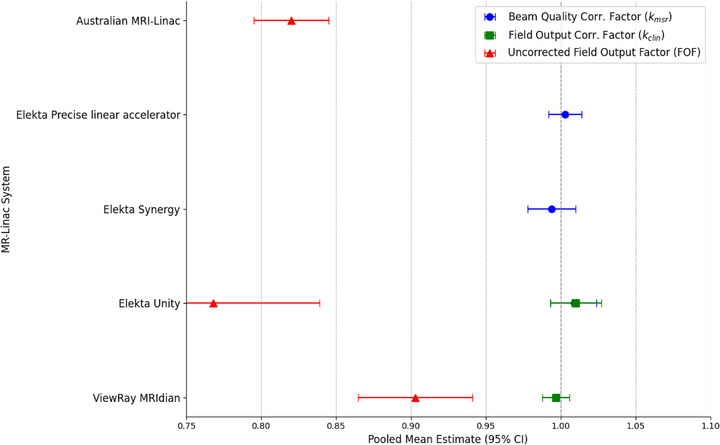
Forest plot comparing the pooled mean estimates and 95% confidence intervals for beam quality correction factor (*k*_msr_), field output correction factor (*k*_clin_), and uncorrected field output factor (FOF) across different MR‐Linac systems (Elekta Unity, Elekta Precise, Elekta Synergy, ViewRay MRIdian, Australian MRI‐Linac). Subgroup heterogeneity (*I* ^2^) is provided for each system.

The primary physical difference between conventional and MRgRT is the strong, static magnetic field. This analysis quantifies its profound and systematic impact on dosimetry. (Figure A3) and the corresponding data in (Table S15) illustrate a clear, monotonic relationship between B‐field strength and the measured dosimetric quantities. As the magnetic field strength increases from 0.35 to 1.5 T, the pooled mean *k_msr_* increases from 0.998to1.009, and the pooled mean *k*_clin_ increases from 0.998to1.010. Concurrently, the uncorrected *FOF* shows a strong inverse trend, decreasing from a pooled mean of 0.902at0.35Tto0.814at1.5T. This demonstrates that the raw detector signal is significantly suppressed in stronger magnetic fields, a direct consequence of the Lorentz force deflecting secondary electrons.^92^ This fundamental physical relationship directly explains the observed dosimetric differences between the two major commercial MR‐Linac systems, which operate at different field strengths. As shown in (Table S16) and (Figure A4), the 1.5 T Elekta Unity and the 0.35 T ViewRay MRIdian exhibit starkly different FOF profiles, a direct consequence of their respective magnetic field strengths.

#### 3.4.5 Influence of field size (meta‐regression)

**FIGURE 4:**
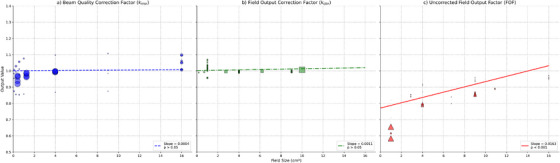
Meta‐regression plots showing the relationship between field size (cm^2^) and (a) *k_msr_*, (b) *k_clin_*, and (c) FOF. The solid line represents the fitted regression line. The slope and p−value of the regression are displayed in each panel. The size of the points is proportional to the inverse variance (weight) of each study.

To rigorously assess the impact of field size, a meta‐regression analysis was performed. The results, visualized in (Figure 4) and detailed in (Table S17), provide a definitive quantification of this relationship. In stark contrast, the uncorrected FOF (Panel c) exhibits a strong, positive, and highly statistically significant linear relationship with field size. The regression model shows that the FOF increases by +0.0163 for every 1 cm^2^ increase in field size (p<0.001). This finding is theoretically expected because the raw signal (FOF) is dominated by geometric and physical effects: as the field size increases, the contribution of phantom scatter increases and the detrimental effect of detector volume averaging is diminished, both of which serve to increase the raw measured signal.

Conversely, for both *k*_msr_ and *k*_clin_ (Panels a and b), the analysis revealed no statistically significant dependence on field size (*p* > 0.05), with regression lines that are essentially flat. This outcome is theoretically robust and desirable. The purpose of *k*_clin_ is to account for the detector's intrinsic response to beam quality and deviation from water equivalence, isolating these effects from the geometric field size dependence. While theoretically a subtle dependence might exist, this non‐significant result implies that the extreme heterogeneity (*I* ^2^ > 92) present in the literature data overwhelms any underlying physical trend, masking the expected variation of *k*_msr_ and *k*_clin_ as a function of field size. This conclusion was rigorously validated by sensitivity checks performed: a subgroup analysis excluding plastic scintillators still yielded a non‐significant dependence (*p* > 0.05), and a further analysis restricted to only the smallest fields (≤ 1 × 1 cm^2^) also found no statistically significant dependence, confirming the robustness of this finding across all SF conditions.

#### 3.4.6 Complex impact of detector orientation

**FIGURE A5:**
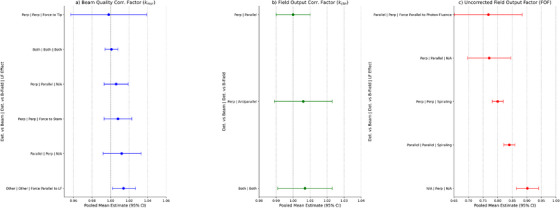
Forest plot examining the influence of detector orientation (relative to the beam and magnetic field) on the pooled mean estimates and 95% confidence intervals for beam quality correction factor (*k*_msr_), field output correction factor (*k*_clin_), and uncorrected field output factor (FOF). Specific orientations and their corresponding Lorentz force effects (where applicable) are indicated on the *y*‐axis. Subgroup heterogeneity (*I* ^2^) is reported for each orientation category.

The orientation of a detector relative to the beam and the magnetic field is a critical parameter that dictates the direction and magnitude of the Lorentz force on secondary electrons. The data, specifically drawn from small field measurements in MR‐Linac systems, are visualized in (Figure A5) (supported by (Table S18) for *k*_msr_) and demonstrates that this is not a minor perturbation but a first‐order effect that systematically alters measured values. For *k*_msr_, distinct differences are observed based on the orientation. For a detector oriented perpendicular to the beam, the pooled mean is 1.008 when the Lorentz force is directed toward the detector stem, but it changes to 0.998 when the force is directed toward the tip. A parallel orientation yields yet another value of 1.012. These systematic shifts provide direct physical evidence of the Lorentz force altering electron trajectories within and around the detector's sensitive volume, thereby changing the collected charge and the resulting signal.^88^ This finding elevates detector orientation from a simple methodological detail to a primary dosimetric parameter that must be rigorously specified and controlled.

#### 3.4.7 Comparison of generation methods

**FIGURE A6:**
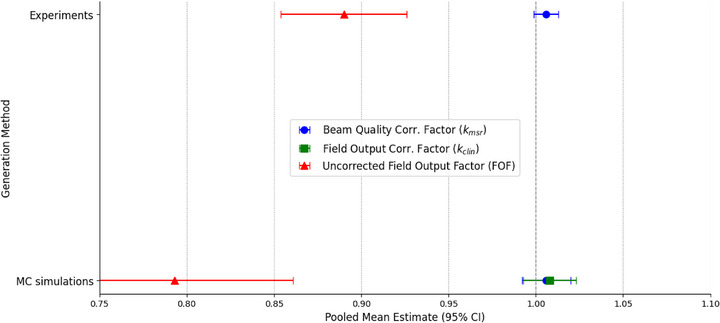
Forest plot comparing pooled mean estimates and 95% confidence intervals for beam quality correction factor (*k*_msr_), field output correction factor (*k*_clin_), and uncorrected field output factor (FOF) between experimental measurements and Monte Carlo (MC) simulations. Subgroup heterogeneity (*I* ^2^) is reported for each method.

The analysis also compared data derived from experimental measurements with those from MC simulations, as shown in (Figure A6) and detailed in (Table S19). For the beam quality correction factor *k_msr_*, the agreement is excellent, with both methods yielding an identical pooled mean estimate of 1.006. This provides strong confidence in the ability of modern MC codes to accurately model the complex physics of detector response in a magnetic field. However, a notable discrepancy was observed for the uncorrected FOF. The pooled mean FO*F* from experimental studies was 0.890[95%CI:0.854,0.926], significantly higher than the 0.793[95%CI:0.725,0.861] derived from MC simulations. This significant difference is likely a reflection of variance in specific machine characteristics, such as the true size and profile of the radiation source in experimental Linacs. As has been shown in the literature, even minor variations in source size can lead to substantial differences in small field FOF measurements,^93^ explaining the observed spread between idealized MC models and real‐world results.

## 4 DISCUSSION

This systematic review and meta‐analysis provides a comprehensive evaluation of the current state of SF photon dosimetry in MRgRT. The results systematically dissect the sources of the high heterogeneity observed in the literature, confirming that the magnetic field strength, detector model, and measurement geometry are the dominant factors influencing dosimetric accuracy.

The most profound finding is the systematic and quantifiable impact of the magnetic field. The observed trends are a direct manifestation of the Lorentz force acting on secondary electrons produced by photon interactions.^92^ In the transverse B‐field configuration common to both the Elekta Unity and ViewRay MRIdian systems, this force deflects electrons, leading to a fundamentally asymmetric dose deposition kernel. This physical principle underpins the significant dosimetric differences observed between the 1.5 and 0.35 T platforms. This electron deflection also gives rise to the *ERE* at tissue‐air interfaces, where electrons exiting a dense medium into air are forced to curve back, depositing excessive dose at the exit surface. The air‐filled sensitive volume of an ionization chamber acts as such an interface. The Lorentz force alters the path lengths of electrons within the air cavity and modifies charge collection efficiency, providing a physical explanation for the strong dependence of detector response on both B‐field strength and orientation relative to the field, as demonstrated in this analysis.^88^

This analysis moves beyond this general physical explanation to provide a quantitative‐qualitative link for why specific detector models behave so differently. The meta‐analysis revealed that a “one‐size‐fits‐all” correction for a given detector type is statistically indefensible; for instance, the PTW 31010 Semiflex ion chamber showed a pooled *k*_msr_ of 1.033, while the PTW 31016 PinPoint 3D had a value significantly below unity at 0.967. Our qualitative synthesis (which reviewed 73 additional papers) provides the physical explanation for this large quantitative discrepancy. The literature consistently identified that a detector's response is not a simple function of its air cavity, but a complex interplay of its entire physical construction.^15^ Specifically, the shape and size of the sensitive volume, along with the presence and geometry of high‐density, high‐Z extracameral components such as the central electrode and guard rings are critical determinants of the final correction factor.^16^ These components perturb the local electron fluence,^15^ and the Lorentz force acts on electrons within these different materials, leading to the unique, model‐specific response that the meta‐analysis has now quantified.

This integration of findings extends across the entire analysis.

- **On Detector Type**: The meta‐analysis quantitatively shows that ion chambers, as a type, have the lowest pooled FOF (0.825), while plastic scintillators have the highest (0.883). Our qualitative synthesis provides the physical reason: ion chambers are highly susceptible to volume averaging^87^ and perturbations from the ERE within their air cavities.^34^ Conversely, the qualitative literature consistently praised plastic scintillators for their “near‐water equivalence,” confirming their minimal intrinsic B‐field dependence.^47^
- **On B‐Field Strength**: Another key quantitative finding from our meta‐regression is the strong, inverse relationship between B‐field strength and the uncorrected FOF: the FOF drops from 0.902 at 0.35 T to 0.814 at 1.5 T. The qualitative synthesis and foundational physics studies^92^ explain this as a direct consequence of the Lorentz force. The stronger 1.5 T field deflects secondary electrons more aggressively than the 0.35 T field,^92^ resulting in the significant detector signal suppression that we have quantitatively measured.
- **On Orientation**: Similarly, our quantitative finding that detector orientation is a ‘first‐order effect’ *k*_msr_ differing for ‘Force to Stem’ [1.008] versus ‘Force to Tip’ [0.998]) is fully supported by the physical explanations in the qualitative literature (e.g., Refs. 16, 36). These orientations directly alter electron trajectories and charge collection efficiency.^88^

By linking the quantitative “what” from the meta‐analysis to the qualitative “why” from the broader literature, these findings move from statistical observation to actionable physical guidance for the clinic. The findings also highlight how the magnetic field exacerbates known challenges in conventional small field dosimetry. The volume averaging effect, clearly identified by the low FOF of ion chambers, is likely amplified in an MR‐Linac environment.^94^ The curving electron trajectories mean that a detector's sensitive volume may sample a more heterogeneous portion of the dose profile than in a zero‐field environment.

These results have direct implications for the application of existing dosimetry protocols. This meta‐analysis provides a large‐scale, quantitative synthesis of the magnetic field's influence on these correction factors, offering an empirical perspective on the magnitude of the “magnetic field correction” that must be considered in the dosimetry chain. The results presented herein provide a quantitative dataset that suggests reinforces the caution warranted when directly applying conventional small field dosimetry protocols (e.g., IAEA TRS‐483) in a magnetic field without considering B‐field specific effects.^24^ This work highlights the potential need for further guidance or a dedicated code of practice for MR‐Linac dosimetry.

While this review provides a robust synthesis of the available data, several limitations must also be acknowledged. The primary limitation of the quantitative part of this study is the hierarchical structure of the data. The 441 data points were extracted from only 13 studies, with many studies contributing dozens of nonindependent, nested data points (e.g., from the same detector on the same machine). Our analysis employed a standard random‐effects model that treats each data point as an independent entry, an assumption made to facilitate the detailed subgroup and meta‐regression analyses. This approach may underestimate the true variance and lead to overly precise (i.e., narrow) confidence intervals for the pooled estimates. A more complex multilevel or hierarchical random‐effects model would be a statistically more robust method to account for this intra‐study clustering. This analysis is also inherently limited by the pooling of data from studies that may have subtle but important methodological differences, a challenge common to any meta‐analysis. While the random‐effects model accounts for this statistically, these differences are a key source of the observed heterogeneity, and the high heterogeneity (*I* ^2^ > 92%), though a key finding in itself, limits the precision of the overall pooled estimates. As we have cautioned, these pooled values are not intended as clinical correction factors but rather as statistical tools to dissect the sources of variation. Third, a portion of the quantitative data had to be extracted from published figures rather than tables, as noted in (Table S9). Although a consistent digital scaling method was used, this process introduces a small, unavoidable potential for measurement error compared to using tabulated data directly. Finally, while funnel plot analysis suggested no significant publication bias, the possibility that studies with null or contradictory findings remain unpublished can never be entirely excluded in a systematic review.

## 5 CONCLUSION AND FUTURE OUTLOOK

This comprehensive meta‐analysis has systematically quantified the principal factors affecting small field dosimetry in MR‐guided radiotherapy. The findings demonstrate that dosimetric quantities in MR‐Linacs are profoundly influenced by magnetic field strength, with high‐field (1.5 T) systems exhibiting significantly different dosimetric characteristics compared to low‐field (0.35 T) systems. Detector selection is a paramount aspect of MR‐Linac dosimetry, as significant performance variations exist not only between detector types but, more critically, between specific detector models within the same class. This underscores that model‐specific correction factors are essential for achieving dosimetric accuracy. Furthermore, detector orientation relative to the radiation beam and the magnetic field is a first‐order effect, not a minor perturbation, mandating that orientation be rigorously controlled. Finally, the results presented herein provide a robust, quantitative dataset that suggests existing small field dosimetry protocols require careful consideration and potential adaptation for application in a magnetic field.

Based on the synthesis of the available evidence, several recommendations can be proposed for different audiences. For clinical medical physicists, it is strongly recommended to use detector‐ and model‐specific correction factors that have been validated for the specific magnetic field strength of the clinical system in use. The critical importance of maintaining a consistent and well‐documented detector orientation for all commissioning and quality assurance measurements cannot be overstated.

For the research community, there is a clear need for further experimental studies that directly compare the performance of different detector models, particularly those identified as significant outliers in this analysis. Efforts should also be directed toward developing and validating robust MC models for a wider range of commercial detectors to reconcile observed discrepancies between simulated and experimental data.

For protocol and standards bodies, such as the IAEA and AAPM, the findings of this study may inform the development of future guidance documents or addenda to existing protocols for MRgRT dosimetry. Future guidance could benefit from stratification by magnetic field strength and the inclusion of model‐specific correction factors that account for orientation effects. Such developments would be a valuable step toward enhancing the standardization of dosimetry for this transformative treatment modality.

## CONFLICT OF INTEREST STATEMENT

The authors of this manuscript declare that they have no conflict of interest related to the research or the publication of these findings.

## Supporting information



Supporting Information

Supporting Information

## APPENDIX A SUPPORTIVE AND INFORMATIONAL TEXTS

### TEXT A1. Study selection criteria

Studies were included in this systematic review if they reported original research on photon beam dosimetry for MRgRT systems. The scope was limited to publications from 1January2020to20July2025 to capture the most current state of this rapidly evolving field. Only full research articles published in English were considered; reviews, editorials, conference abstracts, and theses were excluded.

To be included in the quantitative meta‐analysis (Group 1), a study had to meet a stricter set of criteria. It was required to report a specific numerical value for a detector‐specific correction factor (e.g., $k_{B}^{Q}$) or a small‐field output factor for a field size equivalent to or smaller than $4\times 4\;{\mathrm{cm}}^{2}$. This quantitative result must have been accompanied by a measure of statistical uncertainty (e.g., standard deviation, standard error, or confidence interval) to allow for proper weighting in the meta‐analysis. Furthermore, the study had to provide sufficient methodological context, including the specific models of the detector and MR‐Linac system, the magnetic field strength, the field size(s), and the orientation of the detector relative to the magnetic field and beam.

Studies that did not meet all the stringent criteria for the meta‐analysis but still provided valuable information were included in the qualitative synthesis (Group 2). This included studies that: (1) provided quantitative data but only for large fields $\left(\;>4\times 4\;{\mathrm{cm}}^{2}\right)$; (2) performed a comparative analysis of different detectors, techniques, or treatment planning algorithms; (3) offered methodological guidance, best practices, or discussions of challenges in MRgRT dosimetry; (4) validated a system or method using metrics like gamma analysis; (5) explained the fundamental physics of detector response in a magnetic field; or (6) identified knowledge gaps and suggested future research directions. This two‐tiered approach ensured that while the meta‐analysis remained focused and statistically rigorous, the broader context and valuable insights from the wider literature were captured in the narrative review.
