# Supplementary material for: Detector‐specific correction factors for small‐field photon dosimetry in magnetic resonance‐guided radiation therapy: A systematic review and meta‐analysis
Source: Med Phys. 2026 Jan 7;53(1):e70201. doi: 10.1002/mp.70201 (PMC12778898; doi:10.1002/mp.70201)
Supplement: Supplementary file 1 — Supporting Information [file MP-53-0-s001.pdf]

SUPPORTING MATERIALS:

TEXT S1. Systematic Review Method and Study Search Strategies

Table S1. PRISMA 2020 Checklist

Section and Topic	Item #	Checklist Item	Location where reported
**TITLE**			
Title	1	Identify the report as a systematic review.	Title
**ABSTRACT**			
Abstract	2	See PRISMA 2020 for Abstracts checklist.	Abstract
**INTRODUCTION**			
Rationale	3	Describe the rationale for the review in the context of existing knowledge.	Introduction, Paragraphs 1-3
Objectives	4	Provide an explicit statement of the main objective(s) or question(s) the review addresses.	Introduction, Paragraph 4
**METHODS**			
Eligibility criteria	5	Specify the inclusion and exclusion criteria for the review and how studies were grouped for the syntheses.	Methods 2.2, Table 1
Information sources	6	Specify all databases, registers, websites, organisations, citation searching, and other search methods used to identify studies.	Methods 2.3, Supp. Table S1
Search strategy	7	Present the full search strategies for all databases, registers, and websites, including any filters and limits used.	Supp. Table S1
Selection process	8	Specify the methods used to decide whether a study met the inclusion criteria of the review.	Methods 2.4
Data collection process	9	Specify the methods used to collect data from reports, including how many reviewers collected data from each report.	Methods 2.5
Data items	10	List and define all outcomes for which data were sought. Specify	Methods 2.5

		whether outcome data were collected from reports as reported or were calculated or transformed.	
Study risk of bias assessment	11	Specify the methods used to assess risk of bias in the included studies.	Methods 2.5, Appendix D
Effect measures	12	Specify for each outcome the effect measure(s) (e.g., risk ratio, mean difference) used in the synthesis or presentation of results.	Methods 2.6
Synthesis methods	13	Describe the processes used to decide which studies were eligible for each synthesis.	Methods 2.2, Table 1
	14	Describe any methods required to prepare the data for synthesis.	Methods 2.6
	15	Describe the methods used to tabulate or visually display results of individual studies and syntheses.	Methods 2.6
	16	Describe the methods used to synthesise results and provide a rationale for the choice(s).	Methods 2.6, Appendix E
	17	Describe any methods used to explore possible causes of heterogeneity among study results.	Methods 2.6
	18	Describe any sensitivity analyses conducted to assess the robustness of the synthesised results.	Methods 2.6
Reporting bias assessment	19	Describe any methods used to assess risk of bias due to missing results in a synthesis.	Methods 2.6, Results 3.4
Certainty assessment	20	Describe any methods used to assess certainty (or confidence) in the body of evidence for an outcome.	Not Applicable
**RESULTS**			
Study selection	21	Describe the results of the search and selection process, from the number of records identified in the search to the number of studies included in the review.	Results 3.1, Figure 1
Study characteristics	22	Cite each included study and present its characteristics.	Results 3.2, Supp. Table S4, S5
Risk of bias in studies	23	Present assessments of risk of bias for each included study.	Results 3.2, Appendix D
Results of	24	For all outcomes, present, for each	Results 3.4, Figure 2

individual studies		study: (a) summary statistics for each group (where appropriate) and (b) an effect estimate and its precision.	
Results of syntheses	25	For each synthesis, present results for all outcomes that were predefined in the protocol.	Results 3.4, Tables 2-3, Figures 2, 4
Reporting biases	26	Present assessments of risk of bias due to missing results (arising from reporting biases) for each synthesis assessed.	Results 3.4, Figure 3
Certainty of evidence	27	Present assessments of certainty (or confidence) in the body of evidence for each outcome assessed.	Not Applicable
**DISCUSSION**			
Discussion	28	Provide a general interpretation of the results in the context of other evidence.	Discussion, Paragraphs 1-3
	29	Discuss any limitations of the evidence included in the review.	Discussion, Paragraph 4
	30	Discuss any limitations of the review processes used.	Discussion, Paragraph 4
	31	Discuss the implications of the results for practice, policy, and future research.	Discussion, Paragraphs 3, 5
**OTHER INFO**			
Registration and protocol	32	Provide registration information for the review.	Not Applicable
Support	33	Describe sources of financial or non-financial support for the review.	No Support Received.
Competing interests	34	Declare any competing interests of review author.	Not Applicable
Availability of data, code, and other materials	35	Report which of the following are publicly available and where they can be found: template data collection forms; data extracted from included studies; data used for all analyses; analytic code; any other materials used in the review.	The datasets generated and/or analyzed during the current study are available from the corresponding author on reasonable request. Key data are also provided within the manuscript and its Supplementary Materials

Table S2. Detailed Database Search Strategies

Database	Search Query
Web of Science	("magnetic field" OR "magnetic resonance guided radiotherapy" OR "MR-guided radiotherapy" OR "MRgRT" OR "MR-linac" OR "MRI-linac" OR "ViewRay" OR "Elekta Unity") OR SU=("magnetic resonance imaging" (Topic)) AND ("small field dosimetry" OR "correction factors" OR "detector response" OR "ionization chamber" OR "dosimetric accuracy" OR "output factors" OR "beam quality correction") OR SU=("dosimetry" OR "radiometry" (Topic)) AND ("Monte Carlo" OR "experimental validation" OR "commissioning" OR "quality assurance" OR "Monte Carlo Method" (Topic))
Scopus	TITLE-ABS-KEY (("small field dosimetry" OR "correction factors" OR "detector response" OR "ionization chamber" OR "dosimetric accuracy" OR "output factors" OR "beam quality correction" OR "dosimetry" OR "radiometry") AND ("magnetic field" OR "magnetic resonance guided radiotherapy" OR "MR-guided radiotherapy" OR "MRgRT" OR "MR-linac" OR "MRI-linac" OR "ViewRay" OR "Elekta Unity" OR "magnetic resonance imaging") AND ("Monte Carlo" OR "experimental validation" OR "commissioning" OR "quality assurance" OR "Monte Carlo Method"))
PubMed/MEDLINE	(("magnetic field"[tiab] OR "magnetic resonance guided radiotherapy"[tiab] OR "MR-guided radiotherapy"[tiab] OR MRgRT[tiab] OR "MR-linac"[tiab] OR "MRI-linac"[tiab] OR ViewRay[tiab] OR "Elekta Unity"[tiab] OR "magnetic resonance imaging"[Mesh])) AND (("small field dosimetry"[tiab] OR "correction factors"[tiab] OR "detector response"[tiab] OR "ionization chamber"[tiab] OR "dosimetric accuracy"[tiab] OR "output factors"[tiab] OR "beam quality correction"[tiab] OR "Radiotherapy Dosage"[Mesh] OR "Radiometry"[Mesh])) AND (("Monte Carlo"[tiab] OR "experimental validation"[tiab] OR "commissioning"[tiab] OR "quality assurance"[tiab] OR "Monte Carlo Method"[Mesh]))
Google Scholar	("MR-guided radiotherapy" OR MRgRT OR "MRI-linac") AND ("small field dosimetry" OR "ionization chamber") AND ("Monte Carlo" OR "quality assurance")

Table S3. Database Search Filters and Results Summary

Database	Initial Results	Filters Applied	Final Results
Web of Science	153	Publication Years: 1.1.2020 to 7.20.2025; Document Type: Article; Language: English	82
Scopus	363	Publication Years: 1.1.2020 to 7.20.2025; Document Type: Article; Language: English	316
PubMed/MEDLINE	476	Publication Years: 1.1.2020 to 7.20.2025; Language: English; Various Article Type Filters	36
Google Scholar	356	Publication Years: 1.1.2020 to 7.20.2025; Document Type: Research Article (Manual Screen); Language:	20

		English	
Total	1349	(Before Deduplication)	454
After Deduplication	454	(154 duplicates removed)	320

Table S4. Title/Abstract Screening of 320 Articles

ID	Title	Year	DOI	Exclude or Include	Exclusion Reason
**1**	Commissioning a beam line for MR-guided particle therapy assisted by in silico methods	2023	10.1002/mp.16143	Exclude	This study focuses on commissioning a beam line for MR-guided particle therapy (protons and carbon ions), not photon-based MR-guided radiotherapy (MRgRT). Therefore, it falls outside the topic relevance criterion.
**2**	Benchmarking a GATE/Geant4 Monte Carlo model for proton beams in magnetic fields	2020	10.1002/mp.13883	Exclude	This study focuses on proton beams in magnetic fields (MRPT), which is outside the scope of MR-guided photon radiotherapy (MRgRT) and its specific dosimetric challenges.
**3**	Proton dosimetry in a magnetic field: Measurement and calculation of magnetic field correction factors for a plane-parallel ionization chamber	2024	10.1002/mp.16797	Exclude	This study focuses on proton dosimetry in a magnetic field and the determination of magnetic field correction factors (k B,Q) for a plane-parallel ionization chamber in MR-integrated proton therapy (MRiPT). Our review is specifically for MR-guided photon radiotherapy (MRgRT) and dosimetry. Therefore, this article falls outside the topic relevance.
**4**	Characterization of a Commercial Ionization Chamber Array With Scanned Proton Beams for Applications in MRI-Guided Proton Therapy	2025	10.1002/mp.17875	Exclude	Our review is specifically about challenges and solutions for small field dosimetry in MR-guided photon radiotherapy (MRgRT). The dosimetric challenges and detector responses for proton beams in magnetic fields are fundamentally different from those for photon beams. Therefore, this article falls outside the scope.
**5**	MR-guided ion therapy: Detector response in magnetic fields during carbon ion irradiation	2023	10.1002/mp.16631	Exclude	The physics of ion beams (protons and carbon ions) and their interaction with magnetic fields and detectors are fundamentally different from photon beams, leading to distinct dosimetric challenges and solutions. Therefore, this article falls outside the scope of photon-based MRgRT.
**6**	Technical Note: Design and commissioning of a water phantom for proton dosimetry in magnetic fields	2021	10.1002/mp.14605	Exclude	This study describes the design and commissioning of a water phantom for proton dosimetry in magnetic fields, specifically for magnetic resonance (MR)-guided proton therapy. Our review is focused on challenges and solutions for small field dosimetry in MR-guided photon radiotherapy (MRgRT). Therefore, this article falls outside the topic relevance.
**7**	Proton beam dosimetry in the presence of magnetic fields using Farmer-type ionization chambers of different	2023	10.1002/mp.16368	Exclude	This study focuses on proton beam dosimetry in the presence of magnetic fields using Farmer-type

	radii				ionization chambers, relevant for magnetic resonance-guided proton therapy. Our review is specifically for challenges and solutions for small field dosimetry in MR-guided photon radiotherapy (MRgRT). Therefore, this article falls outside the topic relevance
**8**	The magnetic field dependent displacement effect and its correction in reference and relative dosimetry	2022	10.1088/1361-6560/ac4a41	Exclude	This study investigates perturbation correction factors, focusing on the displacement or gradient correction factor, and the shift of the effective point of measurement in a 1.5 T magnetic field for various ionization chambers. While relevant to dosimetry in MR-Linacs, it does not specifically address small field dosimetry (fields <= 4x4 cm2), quantitative data for correction factors and detector response in the context of small fields, or the unique challenges of measuring dose in small fields. The study explicitly uses a 10x10 ccm field, which is a reference field, not a small field.
**9**	Carbon ion beam dosimetry in magnetic fields using Farmer-type ionization chambers of different radii: measurements and simulations	2024	10.1088/1361-6560/ad67a5	Exclude	This study focuses on carbon ion beam dosimetry in magnetic fields using Farmer-type ionization chambers. Our review is specifically for MR-guided photon radiotherapy (MRgRT) and dosimetry. Therefore, this article falls outside the topic relevance.
**10**	Monte Carlo simulation for proton dosimetry in magnetic fields: Fano test and magnetic field correction factors k B for Farmer-type ionization chambers	2023	10.1088/1361-6560/acefa1	Exclude	Our review is specifically about challenges and solutions for small field dosimetry in MR-guided photon radiotherapy (MRgRT). While it involves magnetic fields and characterization of ionization chambers, the use of proton beams means it falls outside the scope of our target modality (photon radiotherapy).
**11**	Magnetic field induced dose effects in radiation therapy using MR‚Äêlinacs		10.1002/mp.16209	Exclude	This is a review article
**12**	Fano cavity test and investigation of the response of the Roos chamber irradiated by proton beams in perpendicular magnetic fields up to 1 T	2024	10.1088/1361-6560/ad311a	Exclude	While it investigates detector response in magnetic fields, the use of proton beams means it falls outside the scope of our target modality (photon radiotherapy).
**13**	Dosimetric Validation and Surface Fit Evaluation of 3D-Printed Dose Boluses for Radiation Therapy Applications	2025	10.5812/ijcm-159515	Exclude	The context is conventional radiation therapy. Therefore, it fails the Topic Relevance criterion for this systematic review.
**14**	EDXRF analysis of Gd-based Tumoral biomarker using a collimated 241Am source in a Bioequivalent phantom	2025	10.1016/j.radphysche m.2025.113017	Exclude	This study focuses on the development and validation of an energy-dispersive X-ray fluorescence system for gadolinium biomarker detection, emphasizing source optimization, sensitivity, and imaging resolution,

					rather than dose validation, measurement workflows, or small field dosimetry specific to MRI-guided radiotherapy systems.
**15**	Innovative use of novel shielding materials for space electronics	2025	10.1016/j.radphysche m.2025.113023	Exclude	because the focus is on space radiation shielding modeling, not clinical dose validation or small field dosimetry workflows relevant to MRI-guided systems.
**16**	Impact of transverse magnetic fields on dose response of a radiophotoluminescent glass dosimeter in megavoltage photon beams	2020	10.1002/mp.14054	Exclude	The field size investigated is a 10x10 cm field, which is a reference field.
**17**	Development of a radiation installation digital twin for simulation of radiation experiments on accelerators	2025	10.1016/j.radphysche m.2025.113024	Exclude	This article describes the development of a digital twin control system for an accelerator-based radiation installation, focusing on automation and simulation for experimental and industrial use; it does not involve dose validation, dose measurement workflows, or small field dosimetry procedures specific to MRI-guided radiotherapy systems.
**18**	Assessment of external radiation exposure dose rates and protective strategies following I-125 permanent prostate implantation treatment	2025	10.1016/j.radphysche m.2025.112920	Exclude	This study assesses external radiation exposure and safety strategies following I-125 prostate brachytherapy using measurements and Monte Carlo simulations, but it does not involve dose validation, dose measurement workflows, or small field dosimetry specific to MRI-guided radiotherapy systems.
**19**	Impact of transverse magnetic fields on dose response of a nanoDot OSLD in megavoltage photon beams	2020	10.1016/j.ejmp.2020. 01.022	Exclude	While this study investigates the dose response of OSLDs in transverse magnetic fields, it does not specifically focus on clinical MR-Linac systems or small field dosimetry relevant to MRgRT. The magnetic field strengths investigated (0.35-3T) are broader than typical clinical MR-Linac systems (1.5T). It is a general detector characterization study in magnetic fields, not directly addressing MRgRT dosimetry challenges.
**20**	3D nPAG dosimeter in X-ray tomography with synchrotron radiation	2025	10.1016/j.radphysche m.2025.112864	Exclude	While this work assesses a gel dosimeter's response to synchrotron X-ray beams for high dose-rate imaging, it does not involve dose validation, dose measurement workflows, or small field dosimetry relevant to MRI-guided radiotherapy systems.
**21**	Dosimetric analysis of magnetic fields impact on 192Ir MicroSelectron-HDR and BEBIG 60Co-HDR brachytherapy sources: A Monte Carlo simulation study using GATE	2025	10.1016/j.radphysche m.2025.112814	Exclude	It does not involve dose validation, small field dosimetry, or dose measurement workflows specific to MRI-guided radiotherapy systems. Therefore, it should be excluded.
**22**	Radiopharmaceutical and Radioembolization Therapy: Clinical Guidance for Medical Physicists in Radiation Oncology	2025	10.1016/j.prro.2024.1 2.005	Exclude	This article summarizes advances and guidance for radiopharmaceutical and radioembolization therapy, focusing on clinical practices and safety, but does not

					involve dose validation, dose measurement workflows, or small field dosimetry specific to MRI-guided radiotherapy systems.
**23**	The effect of magnetic field on Linac based Stereotactic Radiosurgery dosimetric parameters	2020	10.1088/2057-1976/abd2c4	Exclude	This study investigates the effect of a magnetic field on dosimetric parameters for circular cones used in radiosurgery using Monte Carlo simulation. While it addresses the impact of a magnetic field on dose distributions and mentions small circular cones, it is a general simulation study of magnetic field effects on linac beams, not specific to MR-guided radiotherapy systems (like Elekta Unity or ViewRay MRIdian), nor does it present experimental dosimetric data, correction factors, or detailed detector characterization in the context of MRgRT.
**24**	Alanine dosimetry in strong magnetic fields: Use as a transfer standard in MRI-guided radiotherapy	2020	10.1088/1361-6560/ab8148	Exclude	The study concerns a general reference dosimeter, not one primarily used for high-resolution small-field measurements.
**25**	Impact of transverse magnetic fields on water equivalent ratios in carbon-ion radiotherapy	2020	10.1088/1748-0221/15/05/T05006	Exclude	This study investigates the impact of transverse magnetic fields on water equivalent ratios (WER) in carbon-ion radiotherapy and for future MRI-guided C-ion radiotherapy (MRgCT). Our review is specifically focused on challenges and solutions for small field dosimetry in MR-guided photon radiotherapy (MRgRT). Therefore, this article falls outside the topic relevance.
**26**	Case of the Season: 177Lu-DOTATATE Peptide Receptor Radiotherapy With Acute Dialysis Support in a Patient With Chronic Kidney Disease (CKD)	2025	10.1053/j.ro.2025.03.001	Exclude	While this case report discusses the use of 177Lu-DOTATATE PRRT in a patient with severe CKD and on dialysis, focusing on nephrotoxicity management, it does not involve dose validation, dose measurement workflows, or small field dosimetry specific to MRI-guided radiotherapy systems.
**27**	Investigation of Monte Carlo simulations of the electron transport in external magnetic fields using Fano cavity test	2023	10.1016/j.zemedi.2022.07.002	Exclude	It does not specifically address small field dosimetry (fields <= 4x4 cm2), quantitative data for correction factors and detector response in the context of small fields, or the unique challenges of measuring dose in small fields. The study explicitly uses a 10x10 ccm field, which is a reference field, not a small field.Its focus is on the underlying MC methodology rather than direct small-field dosimetry.
**28**	Monte Carlo modeling of the influence of strong magnetic fields on the stem-effect in plastic scintillation detectors used in radiotherapy dosimetry	2021	10.1002/mp.14637	Exclude	It does not specifically address small field dosimetry (fields <= 4x4 cm2), quantitative data for correction factors and detector response in the context of small fields, or the unique challenges of

					measuring dose in small fields. for actual dose measurements in MRgRT. The study's focus is on a specific aspect of detector response, not the direct application to small field dosimetry challenges.
**29**	Real-time radiation beam imaging on an MR linear accelerator using quantitative T1 mapping	2025	10.1002/mp.17720	Exclude	it fails the Topic Relevance criterion, as it falls more into the realm of MRI physics for dosimetry sensing rather than direct MRgRT dosimetry.
**30**	Development of an extended Macro Monte Carlo method for efficient and accurate dose calculation in magnetic fields	2020	10.1002/mp.14542	Exclude	It does not provide quantitative experimental or Monte Carlo data on detector response or correction factors for small fields, nor does it explicitly address the unique challenges of measuring small fields in MRgRT. Its relevance is to the underlying calculation engine, not directly to the empirical aspects of small field dosimetry or detector characterization. The inclusion of proton beams further broadens its scope beyond photon-based MRgRT.
**31**	Development of an omnidirectional rotating Compton camera for imaging 177Lu radioactive contamination	2025	10.1371/journal.pone.0325586	Exclude	it does not involve dose validation, dose measurement workflows, or small field dosimetry specific to MRI-guided radiotherapy systems. Therefore, it should be excluded.
**32**	Dose perturbation calculation of 9 MeV electron beams in the presence of a 1.5 Tesla longitudinal magnetic field and different inhomogeneities: A Monte Carlo study	2025	10.1016/j.nima.2025.170505	Exclude	ts application is specifically to electron therapy for superficial tumors. Our review is focused on challenges and solutions for small field dosimetry in MR-guided photon radiotherapy (MRgRT). Therefore, this article falls outside the topic relevance.
**33**	Multicycle Dosimetric Behavior and Dose‚ÄìEffect Relationships in [177Lu]Lu-DOTATATE Peptide Receptor Radionuclide Therapy	2025	10.2967/jnumed.124.269389	Exclude	Although it involves dosimetry and dose‚Äìeffect correlations, it is centered on targeted radionuclide therapy for neuroendocrine tumors, using SPECT/CT imaging and Monte Carlo modeling for biodistribution and dose estimation.
**34**	Magnetic field influence on the light yield from fiber-coupled BCF-60 plastic scintillators of relevance for output factor dosimetry in MR-linacs	2024	10.1088/2057-1976/ad13aa	Exclude	It does not specifically address small field dosimetry (fields <= 4x4 cm2), quantitative data for correction factors and detector response in the context of small fields, or the unique challenges of measuring dose in small fields. The study's main objective is to quantify the magnetic field's effect on light yield, a fundamental detector property, rather than addressing the specific challenges of small field measurements.
**35**	Experimental validation of a comprehensive fluoroscopy peak skin dose model using four different computational phantoms	2025	10.1002/mp.17737	Exclude	This study focuses on fluoroscopically guided interventional procedures (FGIP) and not MR-guided radiotherapy (MRgRT).
**36**	First application of a high-resolution silicon detector for	2020	10.1002/mp.13871	Exclude	This study focuses on the first application of a high-

	proton beam Bragg peak detection in a 0.95 T magnetic field				resolution silicon detector for proton beam Bragg peak detection in a magnetic field, with implications for MRI-guided proton therapy. Our review is specifically for MR-guided photon radiotherapy (MRgRT) and dosimetry. Therefore, this article falls outside the topic relevance.
**37**	A systematic characterization of plastic scintillation dosimeters response in magnetic fields: II. Monte Carlo simulations	2025	10.1088/1361-6560/add1a8	Exclude	It does not specifically address small field dosimetry (fields <= 4x4 cm2), quantitative data for correction factors and detector response in the context of small fields, or the unique challenges of measuring dose in small fields. Its primary focus is on the fundamental physics and modeling of detector response in magnetic fields, not on small field dosimetry applications or related challenges.
**38**	Towards MR-guided electron therapy: Measurement and simulation of clinical electron beams in magnetic fields	2020	10.1016/j.ejmp.2020.09.001	Exclude	This study focuses on electron therapy in magnetic fields, not photon-based MR-guided radiotherapy (MRgRT), and while it mentions small fields, the core topic (electron beams) falls outside the defined scope of photon MRgRT dosimetry.
**39**	Calculation of magnetic field effects on dose distribution inside a heterogeneous phantom in MR-guided helium ion-therapy using FLUKA Monte Carlo simulation code	2022	10.22034/rpe.2022.336055.1064	Exclude	Our review specifically focuses on challenges and solutions for small field dosimetry in MR-guided photon radiotherapy (MRgRT). While it addresses magnetic field effects and heterogeneity, the different physical properties of helium ions compared to photons (e.g., Bragg peak, charged particle deflection) mean that the dosimetric challenges and solutions are fundamentally different. Therefore, this article falls outside the scope of photon-based MRgRT small field dosimetry.
**40**	Evaluation of 18F-FDG absorbed dose ratios in percent in adult and pediatric reference phantoms using DoseCalcs Monte Carlo platform	2025	10.1016/j.apradiso.2025.111705	Exclude	This study assesses absorbed dose ratios for 18F-FDG in anatomically varied adult and pediatric phantoms using Monte Carlo simulations, focusing on internal dosimetry and radiation sensitivity, not dose validation workflows or small field dosimetry for MRI-guided radiotherapy.
**41**	Fast and accurate peak skin dose estimation method for interventional fluoroscopy patients	2025	10.1002/mp.17667	Exclude	it does not specifically involve dose validation, small field dosimetry, or workflows within MRI-guided radiotherapy systems. Therefore, it should be excluded.
**42**	Clinical reference dosimetry for the 0.5 T inline rotating biplanar Linac-MR	2024	10.1002/mp.16951	Exclude	This study describes a clinical reference dosimetry protocol for a 0.5 T inline rotating biplanar Linac-MR system and validates it using Monte Carlo simulations and external dose audits. While it addresses aspects of

					dosimetry in an MR-Linac, it does not focus on small field dosimetry (<= 4x4 cm^2), nor does it present quantitative data about correction factors or detector response specifically for small fields, which are key inclusion criteria.
**43**	In vivo dosimetry for proton therapy: A Monte Carlo study of the Gadolinium spectral response throughout the course of treatment	2025	10.1002/mp.17625	Exclude	it fails the Topic Relevance criterion, as it's a specialized in-vivo dosimetry method for proton therapy, but not directly related to the MRgRT-specific dosimetric challenges caused by the magnetic field during treatment.
**44**	Monte Carlo study of dosimetric impact of gadolinium contrast medium in transverse field MR-Linac system	2021	10.1016/j.ejmp.2021.05.020	Exclude	This study investigates the dosimetric impact of gadolinium contrast medium in a transverse field MR-Linac system using Monte Carlo methods. While it provides quantitative dosimetric data and is related to MR-Linac systems, its primary focus is on the effect of contrast agents on dose, not on the challenges of small field dosimetry or the characterization of detectors and correction factors for small fields, which are the main inclusion criteria.
**45**	3D-Printed Organ-Realistic Phantoms to Verify Quantitative SPECT/CT Accuracy for 177Lu-PSMA-617 Treatment Planning	2025	10.3390/ph18040550	Exclude	it does not involve dose validation, dose measurement workflows, or small field dosimetry specific to MRI-guided radiotherapy systems. Therefore, it should be excluded.
**46**	Development and Validation of Monte Carlo Methods for Converay: A Proof-of-Concept Study	2025	10.3390/cancers17071189	Exclude	It pertains to device physics and simulation validation rather than clinical dose validation workflows, so it should be excluded.
**47**	Clinical implementation of patient-specific quality assurance for synthetic computed tomography	2025	10.1016/j.phro.2025.100764	Exclude	Its emphasis is on QA procedures rather than detailed dosimetric validation of MRI-based planning datasets. Therefore, it should be excluded.
**48**	Experimental measurement of ionization chamber angular response and associated magnetic field correction factors in MR‚Äêlinac		10.1002/mp.14445	Exclude	While this article presents important measurements of ionization chamber response and correction factors in MR-guided radiotherapy, it does not involve dose validation, dose measurement workflows, or small field dosimetry specific to MRI-guided systems.
**49**	Estimation of dose to a bystander from F-18 FDG patients using Monte Carlo simulation in clinical exposure scenarios	2025	10.1007/s13246-025-01518-0	Exclude	Although this study uses Monte Carlo simulation to estimate doses to bystanders in nuclear medicine, it does not involve dose validation, dose measurement workflows, or small field dosimetry specific to MRI-guided radiotherapy systems.
**50**	Dose mapping of an irradiation chamber of a gamma cell unit using Fricke gel dosimeters and Monte Carlo simulation	2025	10.1016/j.apradiso.2024.111631	Exclude	Not MR-guided radyoterapi (MRgRT) study
**51**	Water calorimetry in MR-linac: Direct measurement of absorbed dose and determination of chamber kQmag	2020	10.1002/mp.14468	Exclude	It does not specifically address small field dosimetry (fields <= 4x4 cm2), quantitative data for

					correction factors and detector response in the context of small fields, or the unique challenges of measuring dose in small fields. There is no mention of the unique challenges or specific data related to small field measurements with a calorimeter or the characterized ionization chamber.
**52**	Promising application of nano-WO3/epoxy composite in intensity-modulated brachytherapy: a simulation study	2025	10.3857/roj.2024.00339	Exclude	This simulation study explores a novel shielding material for brachytherapy applicators but does not involve dose validation, dose measurement workflows, or small field dosimetry specific to MRI-guided systems.
**53**	Determination of the most appropriate radionuclide for knee radiosynovectomy: Assessment of radiation dose, radiation-induced cancer risk, and post-treatment imaging feasibility	2025	10.1016/j.ejmp.2025.104903	Exclude	Although this study evaluates the suitability of various radionuclides for knee radiotherapy, including dose and secondary cancer risks, it does not involve dose validation, dose measurement workflows, or small field dosimetry relevant to MRI-guided radiotherapy systems.
**54**	Three-dimensional source position verification in image-guided high-dose-rate brachytherapy using an XCT-based gel dosimeter	2025	10.1002/mp.17488	Exclude	While this study presents an XCT-based gel dosimeter for three-dimensional source position verification in brachytherapy, it does not involve dose validation, dose measurement workflows, or small field dosimetry specific to MRI-guided radiotherapy systems.
**55**	Utilizing acrylic acid polymer hydrogel for 3-D quality assurance in CyberKnife radiotherapy	2025	10.1016/j.radphyschem.2024.112300	Exclude	It pertains to optical CT-based dosimetry in CyberKnife treatments rather than MRI-based dose validation, so it should be excluded.
**56**	Extension and validation of a GPU-Monte Carlo dose engine gDPM for 1.5 T MR-LINAC online independent dose verification	2021	10.1002/mp.15165	Exclude	It does not specifically address small field dosimetry (fields <= 4x4 cm2), quantitative data for correction factors and detector response in the context of small fields, or the unique challenges of measuring dose in small fields. It mentions "all field sizes" for some comparisons but doesn't provide quantitative data or detailed analysis for small fields in terms of detector response, correction factors, or the specific issues encountered when measuring or calculating dose in such fields. The scope is broad online dose verification for patient plans, not a deep dive into small field challenges.
**57**	Quality assurance of magnetic resonance imaging for a polymer gel dosimeter using a 3D-printed phantom	2025	10.1016/j.radphysche m.2024.112196	Exclude	This study evaluates the quality assurance (QA) of MRI imaging for a polymer gel dosimeter using a 3D-printed phantom across different MRI systems. It presents calibration, linearity, and uncertainty data, but does not include specific dosimetric measurement data such as correction factors, detector response, or

					validation related to small field dosimetry or MR-Linac systems. Its focus is on MRI QA procedures rather than on dosimetry measurements directly relevant to MRgRT. Therefore, it should be excluded.
**58**	Realistic extension of partial-body pediatric CT for whole-body organ dose estimation in radiotherapy patients	2025	10.1016/j.radphyschem.2024.112194	Exclude	t does not involve dose validation, dose measurement, or workflows specific to MRI-guided radiotherapy systems or small field dosimetry. Therefore, it should be excluded.
**59**	Dosimetry Evaluation on the Use of 18F-FDG for PET/CT Imaging using OLINDA/EXM and Geant4 Monte Carlo Simulations ‚Äì A Single Centre Experience	2025	10.21315/mjms-10-2024-800	Exclude	While this study compares dosimetry estimates for 18F-FDG using OLINDA/EXM and Geant4 Monte Carlo simulations, it focuses on dose calculation methods rather than dose validation, measurement workflows, or small field dosimetry specific to MRI-guided radiotherapy systems.
**60**	Gel dosimetry: An overview of dosimetry systems and read out methods	2024	10.1016/j.radmeas.2024.107321	Exclude	This comprehensive overview discusses gel dosimetry systems and readout methods but does not focus on dose validation, measurement workflows, or small field dosimetry specific to MRI-guided radiotherapy.
**61**	Quantitative CT imaging and radiation-absorbed dose estimations of 166Ho microspheres: paving the way for clinical application	2024	10.1186/s41747-024-00511-8	Exclude	t does not involve dose validation, dose measurement workflows, or small field dosimetry specific to MRI-guided radiotherapy systems. Therefore, it should be excluded
**62**	Intraoperative radiation therapy for brain metastasis in a¬†pregnant patient: a¬†case report	2024	10.1007/s00066-024-02239-1	Exclude	This case report discusses intraoperative radiation therapy in a pregnant patient with brain metastasis, but it does not involve dose validation, dose measurement workflows, or small field dosimetry specifically relevant to MRI-guided radiotherapy systems.
**63**	Evaluation of 2D ion chamber arrays for patient specific quality assurance using a static phantom at a 0.35‚ÄØT MR-Linac	2024	10.1016/j.zemedi.2023.12.003	Exclude	Although patient-specific QA plans might implicitly involve small fields, the study does not specifically analyze or provide quantitative data on the arrays' performance or correction factors in the context of small field dosimetry. The focus is on overall plan QA and angular response for a relatively large field.
**64**	‚ÄúsCT-Feasibility‚Äù - a feasibility study for deep learning-based MRI-only brain radiotherapy	2024	10.1186/s13014-024-02428-3	Exclude	As it does not specifically address small field dosimetry or MR-Linac calibration details, it should be excluded.
**65**	Long-term outcomes of more than a decade treating patients with stereotactic body radiation therapy for hepatocellular carcinoma	2024	10.1016/j.ctro.2024.100878	Exclude	This retrospective study reports long-term tumor control and safety outcomes of SBRT for hepatocellular carcinoma but does not involve dose validation, dose measurement workflows, or small field dosimetry specific to MRI-guided radiotherapy systems.

**66**	Recovering the central axis from the crossline dose profiles in Elekta Unity MR-Linac	2025	10.1088/2057-1976/adddc4	Exclude	It does not specifically address small field dosimetry (fields <= 4x4 cm2), quantitative data for correction factors and detector response in the context of small fields, or the unique challenges of measuring dose in small fields. Its main objective is geometric beam characterization rather than small-field dosimetric measurements.
**67**	A methodology for computationally generating phase space files for Monte Carlo simulations applied to treatment plans for medical linear accelerators	2024	10.1016/j.radphysche m.2024.112109	Exclude	The study pertains to the development of a computational methodology for generating phase space files for Monte Carlo simulations in treatment planning for medical linear accelerators. Although it involves dosimetric calculations and clinical systems, it does not explicitly focus on small field dosimetry ($\leq$ 3x3 cm^2), nor does it provide detailed quantitative data such as correction factors or measurements from MR-Linac systems. The primary focus is on simulation methodology rather than experimental dosimetry measurements relevant to MRgRT. Therefore, this article should be excluded.
**68**	Beam modeling and validation for a 1.5 T MR-linac in an alternative treatment planning system	2025	10.1088/1361-6560/adcfed	Exclude	This study focuses on beam modeling and validation for an MR-linac in an alternative treatment planning system (RayStation) and the accuracy of dose calculations, but it does not specifically address small field dosimetry (fields <= 4x4 cm2) or quantitative dosimetric measurements/correction factors related to small field challenges in MRgRT.
**69**	Surgically targeted radiation therapy versus stereotactic radiation therapy: A dosimetric comparison for brain metastasis resection cavities	2024	10.1016/j.brachy.202 4.06.007	Exclude	This study compares different radiotherapy modalities for brain metastasis resection cavities, focusing on dosimetric comparisons and biological effective doses, but it does not involve dose validation, dose measurement workflows, or small field dosimetry specifically relevant to MRI-guided radiotherapy systems.
**70**	Development and clinical application of a GPU-based Monte Carlo dose verification module and software for 1.5 T MR-LINAC	2023	10.1002/mp.16337	Exclude	This study focuses on the development and validation of a GPU-based Monte Carlo dose verification module for the 1.5 T MR-LINAC for general QA and adaptive radiotherapy. While it confirms accuracy, it does not specifically address small field dosimetry (<= 4x4 cm2) or the quantitative data related to correction factors and detector response for these challenging small fields, which are core inclusion criteria.
**71**	Quantifying uncertainties associated with reference dosimetry in an		10.1002/acm2.13962	Exclude	It does not specifically address small field dosimetry

	MR‚ÄêLinac				(fields <= 4x4 cm2), quantitative data for correction factors and detector response in the context of small fields, or the unique challenges of measuring dose in small fields. for initial calibration, not the unique challenges and specific correction factors required for very small field measurements. Therefore, this article falls outside the primary scope of small field dosimetry in MRgRT.
**72**	Commissioning and implementation of a pencil-beam algorithm with a Lorentz correction as a secondary dose calculation algorithm for an Elekta Unity 1.5T MR linear accelerator	2025	10.1002/acm2.14590	Exclude	This study focuses on commissioning a pencil-beam algorithm as a secondary dose calculation in ClearCalc for an Elekta Unity MR-Linac and its implementation into an adaptive workflow. While it involves dose calculations and comparison with measurements for various field sizes, the primary purpose is algorithm commissioning and workflow integration It does not specifically address small field dosimetry (fields <= 4x4 cm2), quantitative data for correction factors and detector response in the context of small fields, or the unique challenges of measuring dose in small fields. The field sizes mentioned are "various," and the focus is on overall agreement (5%/5MU threshold) rather than the unique challenges of small field measurements.
**73**	Commissioning of MRI-guided gynaecological brachytherapy using an MR-linac	2024	10.1088/2057-1976/ad6c54	Exclude	it does not directly align with the core Topic Relevance criteria, which focuses on MR-guided radiotherapy (external beam) and dosimetry in the presence of the magnetic field affecting the beam and detector response.
**74**	Does a peer review group consensus process for MR-Linac patients affect clinical care? Evaluation of impact and feasibility	2024	10.1016/j.ctro.2024.100816	Exclude	it does not directly align with the core Dosimetric Data criterion as defined for this systematic review.
**75**	Model-based machine learning for the recovery of lateral dose profiles of small photon fields in magnetic field	2022	10.1088/1361-6560/ac5bfa	Exclude	its core focus is on a novel machine learning approach for profile recovery and demonstrating its mathematical consistency, It does not specifically address small field dosimetry (fields <= 4x4 cm2), quantitative data for correction factors and detector response in the context of small fields, or the unique challenges of measuring dose in small fields. The study focuses on a methodological solution for data processing, not the raw dosimetric challenges or detector properties in MR-Linacs.
**76**	Investigations on the beam quality correction factor for ionization chambers in high-energy brachytherapy	2024	10.1088/1361-6560/ad638b	Exclude	it is explicitly about brachytherapy dosimetry, not external beam radiotherapy. Our review is specifically

	dosimetry				on "small field dosimetry in MR-guided radiotherapy (MRgRT)," which refers to external beam radiotherapy using MR-Linacs. Therefore, this article falls outside the scope.
**77**	Relative profile measurements in 1.5T MR-linacs: investigation of central axis deviations	2024	10.1088/1361-6560/ad6ed7	Exclude	it does not specifically focus on small field dosimetry (fields ,â§4√ó4¬†cm<br><br>2<br><br>), quantitative data for correction factors ($k{B,Q}$) and detector response in the context of small fields, or the unique challenges of measuring dose in small fields. The field size used for analysis is a reference field.
**78**	Advancements and applications of dosimetry techniques in modern medical radiation therapy: a comprehensive review	2024	10.1007/s10967-024-09517-3	Exclude	it is a review paper
**79**	End-to-end testing for stereotactic radiotherapy including the development of a MULTI-MODALITY phantom	2024	10.1016/j.zemedi.2022.11.006	Exclude	the abstract does not mention the presence or impact of a magnetic field on dosimetry or detector response during irradiation. Therefore, it fails the Topic Relevance criterion for this specific systematic review.
**80**	Acceptance, commissioning and quality assurance of the MRIdian¬Æ: Site experience and three years follow-up; Acceptance, commissioning et assurance qualit√© du MRIdian¬Æ : exp√©rience sur site et suivi sur trois ans	2023	10.1016/j.canrad.2023.01.005	Exclude	Exclude. This study describes the acceptance, commissioning, and quality assurance of the MRIdian¬Æ MR-Linac, including magnetic field impact, image quality, dose profiles, output factors, isocenter, gantry angles, MLC position, and gating latency. While it covers various aspects of MR-Linac dosimetry and mentions dose profiles and output factors, It does not specifically address small field dosimetry (fields <= 4x4 cm2), quantitative data for correction factors and detector response in the context of small fields, or the unique challenges of measuring dose in small fields. or detector response specifically for small fields. The reported agreements are general for measured dose profiles and output factors, not focused on the unique challenges of small fields.
**81**	Commissioning of a motion management system for a 1.5T Elekta Unity MR-Linac: A single institution experience	2025	10.1002/acm2.70005	Exclude	It does not specifically address small field dosimetry (fields <= 4x4 cm2), quantitative data for correction factors and detector response in the context of small fields, or the unique challenges of measuring dose in small fields. and detector response in the context of small fields, or the

					unique challenges of measuring dose in small fields in MRgRT. The focus is on motion management and gating, not the intrinsic challenges of small field measurements themselves.
**82**	MR-linac MLC positioning QA by digitally stitching dual double-exposed films	2024	10.1002/acm2.14325	Exclude	It does not evaluate dose delivery or accuracy in MRI-based radiotherapy workflows. Therefore, it should be excluded.
**83**	Characterization of an MR-compatible motion platform for quality assurance of motion-compensated treatments on the 1.5 T MR-linac	2025	10.1002/mp.17632	Exclude	he study focuses on the characterization of an MR-compatible motion platform for QA of motion-compensated treatments, rather than directly presenting original dosimetric data for small fields in MRgRT or detailed quantitative data like correction factors or detector response. While it involves dosimetry, it's in the context of QA and platform validation, not primary dosimetric measurements for small fields.
**84**	A comprehensive quality assurance protocol for electromagnetic tracking in brachytherapy	2024	10.1002/mp.17017	Exclude	Since it pertains to applicator positioning and geometry verification rather than dose measurement validation in MRI-guided treatments, it should be excluded.
**85**	Initial clinical experience of patient-specific QA of treatment delivery in online adaptive radiotherapy using a 1.5 T MR-Linac	2021	10.1088/2057-1976/abfa80	Exclude	It presents general gamma passing rates It does not specifically address small field dosimetry (fields <= 4x4 cm2), quantitative data for correction factors and detector response in the context of small fields, or the unique challenges of measuring dose in small fields. The study's focus is on the overall QA system and its performance over a year of clinical operation, rather than the unique challenges of small field measurements.
**86**	A clinical validation of the MR-compatible Delta4 QA system in a 0.35 tesla MR linear accelerator	2021	10.1002/acm2.13216	Exclude	This study focuses on the clinical validation of an MR-compatible Delta4 QA system on a 0.35T MR-Linac and the effect of plan complexity on results. While it involves dosimetric measurements for QA, it does not specifically address It does not specifically address small field dosimetry (fields <= 4x4 cm2), quantitative data for correction factors and detector response in the context of small fields, or the unique challenges of measuring dose in small fields. It's a general QA system validation, not a small-field dosimetry study.
**87**	Quality assurance of an established online adaptive radiotherapy program: patch and software upgrade	2024	10.3389/fonc.2024.1358487	Exclude	Exclude. This study describes quality assurance procedures for an established online adaptive radiotherapy program after a software patch

					installation or upgrade, focusing on various aspects of the workflow (UI, beam model, segmentation, dose calculation, optimizer robustness, CT density table, end-to-end absolute dose). While it mentions an MR-Linac system and dose measurements, it does not specifically focus on small field dosimetry, quantitative data for correction factors, or detailed detector response in the context of small fields in MRgRT. It's a general QA protocol paper, not a specific dosimetry study for small fields.
**88**	Frameless trigeminal neuralgia radiosurgery with a dedicated linear accelerator: From equipment commissioning to initial clinical results; Radiochirurgie des n√©vralgies trig√©minales avec acc√©l√©rateur lin√©aire d√©di√© sans cadre invasif : de la mise en service de l'appareil aux premiers r√©sultats cl...	2024	10.1016/j.canrad.2023.07.017	Exclude	The Novalis Tx¬Æ is a conventional linac-based SRS system, not an MR-linac. Therefore, it fails the Topic Relevance criterion for this systematic review, which is specifically on MR-guided radiotherapy (MRgRT) and dosimetry in the presence of magnetic fields.
**89**	Performance characterization of a novel hybrid dosimetry insert for simultaneous spatial, temporal, and motion-included dosimetry for MR-linac	2024	10.1002/mp.16870	Exclude	While this study focuses on a novel dosimetry insert for MR-linacs and includes dosimetric evaluation, it primarily addresses motion-included dosimetry and end-to-end workflow validation for adaptive radiotherapy. It does not specifically investigate small field dosimetry, nor does it present quantitative data about correction factors or detector response in the context of small fields on MR-linac systems. The reported gamma pass rates are for larger field scenarios related to SBRT, and not focused on the specific challenges of small field measurements.
**90**	Radiation source personalization for nanoparticle-enhanced radiotherapy using dynamic contrast-enhanced MRI in the treatment planning process	2024	10.1016/j.radphysche m.2024.111518	Exclude	This study focuses on modeling nanoparticle distribution via MRI and Monte Carlo simulations for treatment planning, but it does not involve dose validation, dose measurement workflows, or small field dosimetry specifically relevant to MRI-guided radiotherapy systems.
**91**	Quality assurance of IMRT treatment plans for a 1.5 T MR-linac using a 2D ionization chamber array and a static solid phantom		10.1088/1361-6560/abbc47	Exclude	It does not specifically address small field dosimetry (fields <= 4x4 cm2), quantitative data for correction factors and detector response in the context of small fields, or the unique challenges of measuring dose in small fields. Its primary contribution is to a general QA methodology for IMRT plans.
**92**	Commissioning measurements on an Elekta Unity MR-Linac	2022	10.1007/s13246-022-01113-7	Exclude	This study details commissioning measurements for an Elekta Unity MR-Linac, including general beam

					quality, output, and gamma comparisons for a large field (22.0 x 22.0 cm2). However, it does not focus on small field dosimetry (<= 4x4 cm2) or present detailed quantitative data on correction factors or detector response specifically for small fields, which is a key inclusion criterion.
**93**	3D gel dosimeter assessment for end-to-end geometric accuracy determination of the online adaptive workflow on the 1.5 T MR-linac	2024	10.1016/j.phro.2024.100664	Exclude	While this study utilizes gel dosimeters for geometric accuracy assessment in an MR-linac end-to-end workflow, its primary focus is on geometric accuracy and reproducibility. It does not specifically address small field dosimetry (fields <= 4x4 cm2), quantitative data for correction factors and detector response in the context of small fields, or the unique challenges of measuring dose in small fields. The study mentions film measurements as a reference, but it doesn't delve into the specific challenges of small field dosimetry in MRgRT. Therefore, it does not meet the specified inclusion criteri
**94**	MV-based relative electron density estimation (iMREDe) for MR-LINAC dose calculation	2024	10.1002/mp.16969	Exclude	it does not directly align with the core Dosimetric Data criterion as defined for this review.
**95**	End-to-end test for fractionated online adaptive MR-guided radiotherapy using a deformable anthropomorphic pelvis phantom	2021	10.1088/1361-6560/ac3e0c	Exclude	This study describes an end-to-end validation of MR-guided prostate radiotherapy using a deformable phantom and dosimetric measurements, but it does not involve dose validation, dose measurement workflows, or small field dosimetry specific to MRI-guided radiotherapy systems.
**96**	Care Pathway at a Cancer Center for the Administration of Radiometabolic Therapy with 177Lu-PSMA in Patients with Metastatic Castration-resistant Prostate Cancer; Metastatik Kastrasyona Diren√ßli Prostat Kanserli Hastalarda 177Lu-PSMA ile Radyometabolik Tedavinin Uygulanmasƒ±na Y√∂nelik Kanser Merke...	2024	10.4274/mirt.galenos.2023.82653	Exclude	This article describes a clinical care model for administering 177Lu-PSMA therapy for prostate cancer, focusing on treatment pathway development and management rather than dose validation, dose measurement workflows, or small field dosimetry in MRI-guided radiotherapy systems.
**97**	MOSFET dosimeter characterization in MR‚Äêguided radiation therapy (MRgRT) Linac		10.1002/acm2.13028	Exclude	It does not specifically address small field dosimetry (fields <= 4x4 cm2), quantitative data for correction factors and detector response in the context of small fields, or the unique challenges of measuring dose in small fields. The scope appears to be a general characterization for overall commissioning and QA, rather than the unique challenges of small field

					measurements.
**98**	PARSIFAL: A toolkit for triple-GEM parametrized simulation	2024	10.1016/j.cpc.2023.109000	Exclude	As it pertains to particle physics instrumentation, it does not meet the criteria for dose measurement or validation in MRI-based radiotherapy workflows and should be excluded.
**99**	Feasibility of Monte Carlo-based patient-specific quality assurance in 1.5 Tesla magnetic resonance-guided online adaptive radiotherapy: a multi-institutional study	2025	10.1016/j.phro.2025.100800	Exclude	This study focuses on the feasibility of Monte Carlo-based patient-specific quality assurance (PSQA) for MR-guided online adaptive radiotherapy. While it involves dosimetry and MR-Linac systems, it does not specifically address small It does not specifically address small field dosimetry (fields <= 4x4 cm2), quantitative data for correction factors and detector response in the context of small fields, or the unique challenges of measuring dose in small fields.
**100**	Therapeutic dose prediction using score-based diffusion model for pretreatment patient-specific quality assurance	2024	10.3389/fonc.2024.1473050	Exclude	it does not involve dose validation, dose measurement, or workflows specific to MRI-guided radiotherapy systems or small field dosimetry. Therefore, it should be excluded.
**101**	Influence of beam quality on reference dosimetry correction factors in magnetic resonance guided radiation therapy	2020	10.1016/j.phro.2020.10.005	Exclude	It does not specifically address small field dosimetry (fields <= 4x4 cm2), quantitative data for correction factors and detector response in the context of small fields, or the unique challenges of measuring dose in small fields. The study's scope is general reference dosimetry, not small-field specific.
**102**	MR-PVHEE: A novel magnetic resonance-guided parallel-beam very high-energy electron radiotherapy system	2024	10.1016/j.radphysche m.2024.111980	Exclude	the dosimetric principles and challenges for VHEE beams, particularly in small fields, are distinct from those of photon beams. Therefore, this article falls outside the scope of "challenges and solutions for small field dosimetry in MR-guided photon radiotherapy."
**103**	The impact of ion chamber components on k B, Q for reference dosimetry in MRgRT		10.1088/1361-6560/ac55e5	Exclude	It does not specifically address small field dosimetry (fields <= 4x4 cm2), quantitative data for correction factors and detector response in the context of small fields, or the unique challenges of measuring dose in small fields., nor does it provide quantitative data or solutions for the unique challenges of measuring small fields. The study's contribution is to refining correction factor<br>calculations for a reference chamber, which is a foundational aspect of overall dosimetry but not specifically targeted at small field challenges.

**104**	Bone Marrow Sparing by Intensity Modulated Proton Beam Therapy in Postoperative Irradiation of Gynecologic Malignancies	2024	10.1177/15330338241252622	Exclude	it does not involve dose validation, small field dosimetry, or workflows specific to MRI-guided radiotherapy systems. Therefore, it should be excluded.
**105**	Beam output checks of a commercial high-field magnetic resonance-guided radiotherapy machine with its on-board megavoltage imager	2023	10.1016/j.phro.2023.100411	Exclude	While the MVI is an on-board device used in MR-Linacs and is part of quality assurance, this paper specifically validates its suitability for weekly output checks against a water tank and ionization chamber
**106**	Converging proton minibeams with magnetic fields for optimized radiation therapy: A proof of concept	2022	10.3390/cancers14010026	Exclude	This study focuses on "Converging proton minibeams with magnetic fields for optimized radiation therapy," specifically investigating Proton MiniBeam Radiation Therapy (pMBRT). Our review is about challenges and solutions for small field dosimetry in MR-guided photon radiotherapy (MRgRT). Therefore, this article, dealing with proton therapy, falls outside the topic relevance.
**107**	Characterization of the on-board megavoltage imager in a magnetic resonance-guided radiotherapy machine for beam output checks	2024	10.1016/j.phro.2024.100558	Exclude	It does not specifically address small field dosimetry (fields <= 4x4 cm2), quantitative data for correction factors and detector response in the context of small fields, or the unique challenges of measuring dose in small fields. or detector response in the context of small fields. The assessment of linearity, repeatability, and alignment is general for beam output, not tailored to the unique challenges of small field measurements
**108**	Automatic 3D Monte-Carlo-based secondary dose calculation for online verification of 1.5 T magnetic resonance imaging guided radiotherapy		10.1016/j.phro.2021.07.003	Exclude	The search results confirm that neglecting the magnetic field has a significant impact on dose distributions, especially in penumbra regions and at interfaces, which are critical for small field accuracy. Therefore, this study, by its own design, does not contribute to understanding or solving small field dosimetry challenges due to the magnetic field in MRgRT.
**109**	Monte Carlo based dosimetry of extraoral photobiomodulation for prevention of oral mucositis	2023	10.1038/s41598-023-47529-3	Exclude	This study uses Monte Carlo modeling to predict light distribution in extraoral photobiomodulation therapy, focusing on protocol development and anatomical variability, rather than dose validation, dose measurement workflows, or small field dosimetry in MRI-guided radiotherapy systems.
**110**	First clinical application of image-guided intraoperative	2023	10.1186/s13014-	Exclude	it targets intraoperative workflow development rather

	electron radiation therapy with real time intraoperative dose calculation in recurrent rectal cancer: technical procedure		023-02374-6		than dose measurement or validation procedures in MRI-based radiotherapy workflows.
**111**	Alteration of skeletal muscle energy metabolism assessed by 31P MRS in clinical routine: Part 2. Clinical application	2023	10.1002/nbm.5031	Exclude	it focuses on methodological validation and statistical analysis rather than direct dosimetric accuracy, dose validation, or small field validation in MRI-guided radiotherapy systems. Therefore, it should be excluded.
**112**	Automatic, machine-agnostic, convolution-based beam, and fluence modeling for Monte Carlo independent dose calculation	2025	10.1002/mp.17822	Exclude	The study focuses on a general Monte Carlo dose calculation method rather than specific dosimetric measurements or correction factors directly related to MR-guided radiotherapy or small fields in that context.
**113**	Feasibility of energy adaptive angular meshing for perpendicular and parallel magnetic fields in a grid based Boltzmann solver	2020	10.1088/2057-1976/ab6e15	Exclude	It does not provide quantitative experimental or Monte Carlo data on detector response or correction factors for small fields, nor does it explicitly address the unique challenges of measuring small fields in MRgRT. Its relevance is to the underlying calculation engine, not directly to the empirical aspects of small field dosimetry.
**114**	Monte Carlo simulation-based patient-specific QA using machine log files for line-scanning proton radiation therapy	2023	10.1002/mp.16747	Exclude	The context is general proton therapy QA, not MR-guided proton therapy. Therefore, it fails the Topic Relevance criterion for this systematic review.
**115**	Novel intraocular shielding device for eye plaque brachytherapy using magnetite nanoparticles: A proof-of-concept study using radiochromic film and Monte Carlo simulations	2023	10.1016/j.brachy.2023.07.008	Exclude	This study focuses on developing a nanoparticle-based intraocular shielding device for eye plaque brachytherapy, emphasizing dose attenuation and Monte Carlo simulations, but it does not involve dose validation, measurement workflows, or small field dosimetry relevant to MRI-guided radiotherapy.
**116**	The neutron dose equivalent rate measurements by R3DR/R2 sp ectrometers on the international space station	2023	10.1016/j.lssr.2023.01.001	Exclude	it does not involve dose validation, dose measurement workflows, or small field dosimetry in MRI-guided radiotherapy systems. Therefore, it should be excluded.
**117**	Technical Note: Cumulative dose modeling for organ motion management in MRI-guided radiation therapy	2021	10.1002/mp.14500	Exclude	This study focuses on a method for cumulative dose modeling for organ motion management in MR-guided radiation therapy and its application to a liver cancer case. While it uses Monte Carlo simulations and is relevant to MRgRT, its primary aim is not small field dosimetry, quantitative dosimetric measurements (e.g., correction factors, detector response specific to small fields), or the challenges associated with the physical measurement of dose in small fields. The targets mentioned are general IMRT fields and liver cancer

					cases, not explicitly small fields (e.g., <= 4x4 cm^2).
**118**	Detailed Monte-Carlo characterization of a Faraday cup for proton therapy	2023	10.1002/mp.16464	Exclude	it falls outside the direct Topic Relevance for MRgRT systems and their specific dosimetric challenges.
**119**	Modern Tools for Modern Brachytherapy	2023	10.1016/j.clon.2023.05.003	Exclude	t primarily addresses innovations, applicator reconstruction, dose calculations, and validation strategies in brachytherapy, not dose validation, dose measurement workflows, or small field dosimetry workflows specific to MRI-guided radiotherapy systems.
**120**	Single- and dual-source-strength focal boost planning in low-dose-rate prostate brachytherapy: feasibility study	2023	10.1017/S1460396923000225	Exclude	It is limited to brachytherapy planning and dose escalation within LDR prostate treatments, not involving MRI-guided radiotherapy systems, small fields, or dose validation specific to MRI-based or MR-Linac dose calculations. Therefore, it should be excluded.
**121**	Associated radiation exposure from medical imaging and excess lifetime risk of developing cancer in pediatric patients with pulmonary hypertension	2023	10.1002/pul2.12282	Exclude	This study estimates radiation doses and associated cancer risks in pediatric pulmonary hypertension patients from imaging, focusing on risk assessment rather than dose validation, measurement workflows, or small field dosimetry relevant to MRI-guided radiotherapy systems.
**122**	Dosimetry Effects Due to the Presence of Fe Nanoparticles for Potential Combination of Hyperthermic Cancer Treatment with MRI-Based Image-Guided Radiotherapy	2023	10.3390/ijms24010514	Exclude	The effects observed are specific to the presence of nanoparticles, which is outside the scope of general small field dosimetry in MRgRT.
**123**	Leaf-individual calibration for a double stack multileaf collimator in photon radiotherapy	2023	10.1016/j.phro.2023.100477	Exclude	it does not involve dose validation, small field dose measurement, or MRI-guided dose verification workflows. Therefore, it should be excluded.
**124**	Lung sparing in MR-guided non-adaptive SBRT treatment of peripheral lung tumors	2024	10.1088/2057-1976/ad567d	Exclude	The study compares lung sparing in MRgSBRT to conventional SBRT and analyzes planning metrics, but it does not focus on small field dosimetry, quantitative dosimetric measurements like correction factors, or detector response specific to MRgRT.
**125**	Quality assurance and safety of hippocampal avoidance prophylactic cranial irradiation in the multicenter randomized phase III trial (NCT01780675)	2023	10.1016/j.jncc.2023.05.004	Exclude	t does not involve dose validation, dose measurement, or small field dosimetry workflows specific to MRI-guided radiotherapy systems. Therefore, it should be excluded.
**126**	Sensitivity of a bone-equivalent polymer gel dosimeter for measuring the dose to bone during radiation therapy	2023	10.1007/s12194-023-00710-9	Exclude	This study involves developing a bone-equivalent gel dosimeter and evaluating its sensitivity to radiation dose, but it does not include dose validation, measurement workflows, or small field dosimetry specific to MRI-guided radiotherapy systems.

**127**	Why we should care about gas pockets in online adaptive MRgRT: a dosimetric evaluation	2023	10.3389/fonc.2023.1280836	Exclude	This study evaluates the dosimetric impact of gas pockets and their electronic density in online adaptive MRgRT, focusing on workflow efficiency and dose calculation accuracy related to gas contouring. While it is about dosimetry in MRgRT, it does not focus on small field dosimetry (<= 4x4 cm^2), quantitative data like correction factors or detector response for small fields, or the challenges of physical dose measurement in small fields.
**128**	Computational Optimization of Irradiance and Fluence for Interstitial Photodynamic Therapy Treatment of Patients with Malignant Central Airway Obstruction	2023	10.3390/cancers15092636	Exclude	It focuses on optical therapy and treatment planning validation, not radiotherapy dose verification. Therefore, it should be excluded.
**129**	Knowledge-based adaptive planning quality assurance using dosimetric indicators for stereotactic adaptive radiotherapy for pancreatic cancer	2023	10.1016/j.radonc.2023.109603	Exclude	it focuses on plan deviations and failure-mode detection rather than direct dose validation, dose measurement, or small field dosimetry in MRI-guided systems. Therefore, it should be excluded.
**130**	Does dose calculation algorithm affect the dosimetric accuracy of synthetic CT for MR-only radiotherapy planning in brain tumors?	2025	10.1002/acm2.70030	Exclude	This study compares the dosimetric accuracy of different dose calculation algorithms for synthetic CT in brain radiotherapy, focusing on algorithm performance rather than dose validation workflows or small field dosimetry specific to MRI-guided systems.
**131**	Absence of Tissue-Sparing Effects in Partial Proton FLASH Irradiation in Murine Intestine	2023	10.3390/cancers15082269	Exclude	This study reports that partial proton FLASH irradiation did not produce tissue-sparing effects in murine intestine, focusing on biological outcomes rather than dose validation, dose measurement workflows, or small field dosimetry relevant to MRI-guided radiotherapy systems.
**132**	Evaluation and comparison of synthetic computed tomography algorithms with 3T MRI for prostate radiotherapy: AI-based versus bulk density method	2025	10.1002/acm2.14581	Exclude	This study compares AI-based and bulk-density-based synthetic CT algorithms for prostate MRI, focusing on image artifacts and dosimetric comparison to treatment planning CT, without involving dose validation, dose measurement workflows, or small field dosimetry specific to MRI-guided systems.
**133**	Photobiomodulation for knee osteoarthritis: a model-based dosimetry study	2023	10.1364/BOE.484865	Exclude	it does not involve dose validation, dose measurement workflows, or small field dosimetry specific to MRI-guided radiotherapy systems. Therefore, it should be excluded
**134**	SARU: A self-attention ResUNet to generate synthetic CT images for MR-only BNCT treatment planning	2023	10.1002/mp.15986	Exclude	While this study is related to MR-guided radiotherapy, it focuses on generating synthetic CT images for BNCT treatment planning, not specifically on MR-guided radiotherapy (MRgRT) dosimetry. The study doesn't present dosimetric measurements or correction factors for MR-Linac systems, which are the main focus of the inclusion criteria.

**135**	An ESTRO-ACROP guideline on quality assurance and medical physics commissioning of online MRI guided radiotherapy systems based on a consensus expert opinion	2023	10.1016/j.radonc.2023.109504	Exclude	While critical for clinical implementation, it is not an original dosimetric validation study or a direct assessment of dose accuracy or small field dosimetry in MRI-based treatments. Therefore, it should be excluded.
**136**	Validation of an EGSnrc-based Monte Carlo model for a complex 2D-array for technical QA measurements of a linear accelerator	2023	10.1002/mp.16205	Exclude	it fails the Topic Relevance criterion for current MRgRT and dosimetry in magnetic fields.
**137**	ACPSEM position paper: dosimetry for magnetic resonance imaging linear accelerators	2023	10.1007/s13246-023-01223-w	Exclude	It does not present original research data and are akin to review articles or opinion pieces. Therefore, it fails the Original Research? criterion.
**138**	Quality assurance for MRI-only radiation therapy: A voxel-wise population-based methodology for image and dose assessment of synthetic CT generation methods	2022	10.3389/fonc.2022.968689	Exclude	This study presents a voxel-wise, population-based methodology for assessing local errors in synthetic CT generation and their impact on dose, primarily focused on prostate cancer radiotherapy, without involving dose validation workflows, dose measurement procedures, or small field dosimetry specific to MRI-guided radiotherapy systems.
**139**	A Realistic Multiregion Mouse Kidney Dosimetry Model to Support the Preclinical Evaluation of Potential Nephrotoxicity of Radiopharmaceutical Therapy	2023	10.2967/jnumed.122.264453	Exclude	This study focuses on detailed mouse kidney suborgan dosimetry modeling to support radiopharmaceutical therapy assessment, but it does not involve dose validation, dose measurement workflows, or small field dosimetry relevant to MRI-guided radiotherapy systems.
**140**	Monte Carlo Dose Calculation Using MRI Based Synthetic CT Generated by Fully Convolutional Neural Network for Gamma Knife Radiosurgery	2021	10.1177/15330338211046433	Exclude	This study investigates the dosimetric impact of synthetic CT generated by deep learning for Gamma Knife radiosurgery, focusing on dose differences and MAE metrics, but it does not involve dose validation workflows, dose measurement processes, or small field dosimetry specific to MRI-guided systems.
**141**	Analysis of online plan adaptation for 1.5T magnetic resonance-guided stereotactic body radiotherapy (MRgSBRT) of prostate cancer	2023	10.1007/s00432-022-03950-1	Exclude	Without direct dosimetric evaluation or results presented in the abstract, it does not meet the "Dosimetric Data" criterion for inclusion in this systematic review.
**142**	New Capabilities of the FLUKA Multi-Purpose Code	2022	10.3389/fphy.2021.788253	Exclude	it is a review of the code itself and its broad capabilities, not a specific study on small field dosimetry challenges or solutions in MR-guided photon radiotherapy. It does not provide quantitative data, specific detector characterizations, or detailed discussions on small field behavior within MR-Linacs. While FLUKA can be used for such simulations, this article does not present findings directly relevant to the topic.
**143**	Adaptive hypofractionted and stereotactic body	2023	10.3389/fonc.2023.1	Exclude	the abstract provides a general overview and outlines

	radiotherapy for lung tumors with real-time MRI guidance		061854		"steps" or "considerations" rather than presenting original research with specific quantitative dosimetric data. it fails the Original Research? and Dosimetric Data criteria.
**144**	Volumetric modulated arc therapy (VMAT) for hippocampal-avoidance whole brain radiation therapy: Planning comparison with Dual-arc and Split-arc partial-field techniques	2020	10.1186/s13014-020-01488-5	Exclude	The study is on conventional radiotherapy (VMAT for WBRT) and does not involve MR-guided radiotherapy.
**145**	Prediction of tumor control in 90Y radioembolization by logit models with PET/CT-based dose metrics	2020	10.2967/jnumed.119.226472	Exclude	This involves radionuclide therapy (radioembolization), which is fundamentally different from external beam radiotherapy (EBRT) using linear accelerators. Our review is specifically about "small field dosimetry in MR-guided radiotherapy (MRgRT)," which refers to external beam radiotherapy delivered by MR-Linacs. Therefore, this article falls outside the scope.
**146**	Assessment of dose to vaginal mucosa for gynecologic template interstitial high-dose-rate brachytherapy using Monte Carlo simulation	2023	10.5114/jcb.2023.131781	Exclude	This article focuses on gynecologic high-dose-rate brachytherapy and not on MR-guided radiotherapy (MRgRT).
**147**	Toward three-dimensional patient-specific internal dosimetry using GATE Monte Carlo technique	2022	10.1016/j.radphyschem.2022.110046	Exclude	This study focuses on patient-specific internal dosimetry in nuclear medicine using GATE Monte Carlo technique for F-18 FDG, not external beam MR-guided radiotherapy (MRgRT) or its associated dosimetry challenges.
**148**	Feasibility of flattening filter free beams for hippocampal avoidance whole-brain radiotherapy: a dosimetric and radiobiological analysis	2023	10.3389/fonc.2023.1290434	Exclude	While it discusses dose delivery and quality assurance, it does not specify dose validation procedures, dose measurement workflows, or small field dosimetry techniques relevant to MRI-guided settings.
**149**	Quality and Safety Considerations in Stereotactic Radiosurgery and Stereotactic Body Radiation Therapy: An ASTRO Safety White Paper Update	2022	10.1016/j.prro.2022.03.001	Exclude	does not specifically delve into the unique challenges of small field dosimetry in the presence of a magnetic field (i.e., within an MR-Linac system) or provide quantitative data on detector behavior, correction factors, or solutions specific to MRgRT small fields. It is a general safety guideline for SRS/SBRT, not focused on the distinct MR-Linac environment.
**150**	Radiotherapy planning in a prostate cancer phantom model with intraprostatic dominant lesions using stereotactic body radiotherapy with volumetric modulated arcs and a simultaneous integrated boost	2023	10.3389/fonc.2023.1147593	Exclude	The core challenge for our review is small field dosimetry in the presence of magnetic fields (MRgRT). This paper is a general SBRT-VMAT dosimetry study in a phantom, without the critical element of magnetic field effects. Therefore, it falls outside the scope of our review.
**151**	A novel preclinical model of craniospinal irradiation in pediatric diffuse midline glioma demonstrates decreased metastatic disease	2023	10.3389/fonc.2023.1105395	Exclude	This study presents a preclinical model demonstrating reduced metastasis with craniospinal irradiation in pediatric diffuse midline glioma, but it does not

					involve dose validation, dose measurement workflows, or small field dosimetry relevant to MRI-guided radiotherapy systems.
**152**	Fixed field technique for hippocampal avoidance whole-brain radiotherapy: A feasibility study using Elekta system	2023	10.52547/ijrr.21.1.14	Exclude	This article focuses on fixed field volumetric modulated arc therapy (fVMAT) for hippocampal avoidance whole-brain radiotherapy (ha-WBRT) using the Elekta Agility,Ñ¢ collimator system and Monaco treatment planning system. it fails the Topic Relevance criterion for this systematic review.
**153**	Development of a multi-purpose quality control phantom for MRI-based treatment planning in high-dose-rate brachytherapy of cervical cancer	2023	10.5114/jcb.2023.125014	Exclude	This study focuses on the "Development of a novel multi-purpose QC phantom (AQuA-BT) and examples of its' application in 3D image-based (particularly magnetic resonance imaging [MRI]-based) planning for cervix HDR-BT." While it involves MRI and dosimetry, it pertains to brachytherapy (BT), which is a form of internal radiation therapy using radioactive sources placed directly within or next to the area requiring treatment. Our review is specifically about "small field dosimetry in MR-guided external beam radiotherapy (MRgRT) using linear accelerators." The dosimetric challenges, treatment delivery methods, and phantom requirements for brachytherapy are fundamentally different from those of external beam radiotherapy with MR-Linacs. Therefore, this article falls outside the scope.
**154**	Development of a computational pregnant female phantom and calculation of fetal dose during a photon breast radiotherapy	2022	10.2478/raon-2022-0039	Exclude	because its focus is on modeling fetal dose in radiotherapy, not on dose measurement validation workflows.
**155**	Dosimetric and biological comparisons of single planning and double plannings for bilateral lung cancer SBRT planning based on the Cyber-Knife system	2022	10.3389/fonc.2022.1015999	Exclude	it does not involve dose validation, small field dosimetry, or workflows specific to MRI-guided radiotherapy systems. Therefore, it should be excluded.
**156**	Effect of the normal liver mean dose on intrahepatic recurrence in patients with hepatocellular carcinoma after receiving liver stereotactic body radiation therapy	2022	10.1016/j.tranon.2022.101492	Exclude	This study investigates the relationship between normal liver mean dose and intrahepatic recurrence after liver SBRT for hepatocellular carcinoma. While it involves dosimetric parameters and clinical outcomes, it does not address MR-guided radiotherapy, MR-Linac systems, or small field dosimetry related to MR-based treatments. Its focus is on hepatic SBRT dose-response, not on MRI-based dosimetry accuracy or small fields in MRgRT, so it should be excluded.
**157**	MRI-LINAC dosimetry approach by Monte Carlo codes coupling charged particle radiation transport with strong magnetic fields	2022	10.1016/j.radphysche m.2022.110171	Exclude	While it is on topic and uses Monte Carlo, it fails the Dosimetric Data criterion as defined in the detailed abstract screening questions.

**158**	Commissioning and dosimetric results of an indigenously developed intra-vaginal template for interstitial plus intracavitary high dose rate image-guided brachytherapy of advanced cervix cancer	2022	10.4103/jmp.jmp_50_22	Exclude	This study presents commissioning and dosimetric outcomes of an indigenously developed intracavitary plus interstitial template for cervix HDR brachytherapy, focusing on parameter validation and dose metrics, but it does not involve dose validation, dose measurement workflows, or small field dosimetry in MRI-guided radiotherapy.
**159**	Practical guidelines of online MR-guided adaptive radiotherapy	2022	10.1093/jrr/rrac048	Exclude	review
**160**	Design and commissioning of an e-beam irradiation beamline at the Upgraded Injector Test Facility at Jefferson Lab	2022	10.1016/j.nima.2022.167093	Exclude	The systematic review's core topic is "MR-guided radiotherapy (MRgRT) and dosimetry," which specifically refers to medical applications of radiation therapy. Therefore, it fails the Topic Relevance criterion.
**161**	Stereotactic Radioablation for Ventricular Tachycardia in the Setting of Electrical Storm	2022	10.1161/CIRCEP.122.010955	Exclude	This study discusses the use of stereotactic radiotherapy to treat ventricular tachycardia in patients with electrical storm, focusing on clinical outcomes and recurrence patterns, but it does not involve dose validation, dose measurement workflows, or small field dosimetry specific to MRI-guided radiotherapy systems.
**162**	Investigation the effect of a magnetic field on the dose distribution of I-125, Ir-192, Yb-169, and Co-60 brachytherapy sources by Monte Carlo simulation	2022	10.1016/j.apradiso.2022.110332	Exclude	it does not meet the Topic Relevance criterion for this systematic review, as it is not about MR-guided external beam radiation therapy.
**163**	Scalable radiotherapy data curation infrastructure for deep-learning based autosegmentation of organs-at-risk: A case study in head and neck cancer	2022	10.3389/fonc.2022.936134	Exclude	t does not directly involve dose validation, small field dosimetry, or dose measurement workflows specific to MRI-guided treatments. Therefore, it should be excluded.
**164**	Mouthpiece polymer-gel dosimeter for in vivo oral dosimetry during head and neck radiotherapy	2022	10.1016/j.apradiso.2022.110301	Exclude	it does not involve dose validation, small field dosimetry, or workflows specific to MRI-guided radiotherapy systems. Therefore, it should be excluded.
**165**	An external perpendicular magnetic field does not influence survival and DNA damage after proton and carbon ion irradiation in human cancer cells	2022	10.1016/j.zemedi.2021.11.001	Exclude	it fails the Dosimetric Data criterion, as its primary focus is not physical dosimetry for MRgRT.
**166**	Dosimetric Comparison of Volumetric Modulated Arc Therapy and Intensity Modulated Radiation Therapy for Soft Tissue Sarcoma of the Extremities	2022	10.1016/j.prro.2022.02.013	Exclude	It concerns conventional external beam techniques without MRI- or MR-Linac-specific dose validation. Therefore, it should be excluded.
**167**	Domain adaptation of automated treatment planning from computed tomography to magnetic resonance	2022	10.1088/1361-6560/ac72ec	Exclude	it fails the Topic Relevance criterion, as it does not address dosimetry in the presence of the magnetic field as defined for this systematic review.
**168**	Dose Rate Assessment Exercises with Stylized Phantom of Neon Flying Squid from Northwest Pacific	2022	10.3390/jmse10060782	Exclude	Therefore, I would classify it as: Exclude ‚Äî because its focus is ecological environmental dosimetry and not related to clinical dose validation workflows.

**169**	A Monte Carlo study on the effects of a static uniform magnetic field on micro-scale dosimetry of Auger-emitters using Geant4-DNA	2022	10.1016/j.radphysche m.2022.110063	Exclude	it fails the Topic Relevance criterion for this specific review.
**170**	External beam irradiation angle measurement using a hybrid Cerenkov-scintillation detector	2022	10.1088/1361-6560/ac6b79	Exclude	The study does not present any data or analysis on how the magnetic field of an MR-Linac would influence the detector's response or the accuracy of angle measurements. Therefore, it fails the Topic Relevance criterion for this systematic review, as it does not address dosimetry in the presence of magnetic fields for MRgRT.
**171**	Application of 3D Gel Dosimetry as a Quality Assurance Tool in Functional Leksell Gamma Knife Radiosurgery	2022	10.3390/gels802006 9	Exclude	While it addresses "small 4 mm collimator" and high-precision dosimetry for brain diseases, it is specifically focused on Gamma Knife radiosurgery, which is a dedicated system for intracranial stereotactic treatment that does not involve a magnetic field during dose delivery. Our review is specifically about challenges and solutions for small field dosimetry in MR-guided radiotherapy (MRgRT), which implies the presence of a strong magnetic field during treatment. Therefore, this article falls outside the scope of MRgRT small field dosimetry.
**172**	Dosimetry of small photon fields in the presence of bone heterogeneity using MAGIC polymer gel, Gafchromic film, and Monte Carlo simulation	2022	10.5603/rpOr.a2022. 0031	Exclude	it does not involve MRI-guided radiotherapy, MR-Linac systems, or small field validation specific to MRI-based or MR-guided treatments. Therefore, it should be excluded.
**173**	Dosimetric Comparison of Volumetric-Modulated Arc Therapy and Dynamic Conformal Arc Therapy in Threefraction Single-isocenter Stereotactic Radiosurgery for Multiple Brain Metastases	2022	10.5505/tjo.2021.341 2	Exclude	This study compares VMAT and DCAT techniques in multifraction SRS for brain metastases focusing on plan quality and dose metrics but does not involve dose validation, dose measurement workflows, or small field dosimetry specific to MRI-guided systems.
**174**	A standard test phantom for the performance assessment of magnetic resonance guided high intensity focused ultrasound (MRgHIFU) thermal therapy devices	2022	10.1080/02656736.2 021.2017023	Exclude	this article does not meet the Topic Relevance criterion for this systematic review.
**175**	Deep Seated Tumour Treatments With Electrons of High Energy Delivered at FLASH Rates: The Example of Prostate Cancer	2021	10.3389/fonc.2021.7 77852	Exclude	It does not involve dose validation, dose measurement, or small field dosimetry protocols specific to MRI-guided radiotherapy systems. Therefore, it should be excluded.
**176**	Dosimetric Comparison of Intraoperative Radiotherapy and SRS for Liver Metastases	2021	10.3389/fonc.2021.7 67468	Exclude	It does not evaluate MRI-guided dose measurement, small field dosimetry, or dose validation workflows specific to MRI-based radiotherapy systems. Therefore, it should be excluded.

**177**	Validation of a monte carlo modelling based dosimetry of extraoral photobiomodulation	2021	10.3390/diagnostics11122207	Exclude	PBMT is a light-based therapy, not radiation therapy (radiotherapy/linac) using ionizing radiation. Therefore, it does not meet the Topic Relevance criterion for this systematic review.
**178**	Can Radiation Therapy Quality Assurance Improve Nasopharyngeal Cancer Outcomes in Low- and Middle-Income Countries: Reporting the First Phase of a Prospective International Atomic Energy Agency Study	2021	10.1016/j.ijrobp.2021.08.013	Exclude	it does not involve dose validation, dose measurement workflows, or small field dosimetry specific to MRI-guided radiotherapy systems. Therefore, it should be excluded.
**179**	Numerical investigation of plasma behavior in a micro DC ion thruster using the particle-in-cell/Monte Carlo collision (PIC/MCC) method	2021	10.1088/1361-6463/ac1a0c	Exclude	The study focuses on plasma behavior in a micro DC ion thruster using PIC/MCC simulation, which is not directly related to MR-guided radiotherapy or dosimetry. It presents original research on plasma physics and propulsion systems, not on medical dosimetry or clinical MR-Linac systems. Therefore, it does not meet the inclusion criteria for the systematic review.
**180**	Investigation of isotropic radiation of low energy X-ray intra-operative radiotherapy by MAGAT gel dosimeter	2021	10.1016/j.radphyschem.2021.109648	Exclude	This study evaluates the isotropy of low-energy X-ray intra-operative radiotherapy using a 3D gel dosimeter and phantom, focusing on system accuracy and phantom design rather than dose validation workflows or small field dosimetry in MRI-guided radiotherapy.
**181**	Investigation the trend of different magnetic fields types on linac photon beam mode by Monte Carlo method using Geant4 toolkit	2021	10.1016/j.radphyschem.2021.109603	Exclude	it fails the Clinical Systems criterion, as the study is not conducted on actual clinical MR-Linac systems or Monte Carlo simulations validated with these systems in their standard operational modes.
**182**	Dosimetric Comparison of Upfront Boosting With Stereotactic Radiosurgery Versus Intraoperative Radiotherapy for Glioblastoma	2021	10.3389/fonc.2021.759873	Exclude	Not MR-guided radiotherapy (MRgRT) study
**183**	Characterisation of a synthetic diamond detector for end-to-end dosimetry in stereotactic body radiotherapy and radiosurgery	2021	10.1016/j.phro.2021.10.002	Exclude	The context is general SBRT/SRS applications, not specifically within an MR-linac environment. Therefore, it fails the Topic Relevance criterion for this systematic review.
**184**	Study of relationship between dose, LET and the risk of brain necrosis after proton therapy for skull base tumors	2021	10.1016/j.radonc.2021.08.015	Exclude	It does not involve dose validation, dose measurement workflows, or small field dosimetry specific to MRI-guided systems. Therefore, it should be excluded.
**185**	InterDosi simulations of photon and alpha specific absorbed fractions in zubal voxelized phantom	2021	10.1016/j.apradiso.2021.109838	Exclude	While relevant for internal dose calculations and targeted alpha therapy, it does not involve dose validation, small field dosimetry, or workflows specific to MRI-guided radiotherapy systems. Therefore, it should be excluded.
**186**	Verification of imrt and rapidarc localized prostate cancer treatment plans using epid and delta4 in vivo	2021	10.30476/mejc.2020.84706.1226	Exclude	The abstract makes no mention of MR-guidance or the presence of a magnetic field affecting dose

	dosimetry methods				delivery or dosimetry. it fails the Topic Relevance criterion for this systematic review.
**187**	AAPM Task Group 241: A medical physicist‚Äôs guide to MRI-guided focused ultrasound body systems	2021	10.1002/mp.15076	Exclude	This report focuses on clinical implementation, safety, and quality assurance procedures for MRI-guided focused ultrasound systems, but it does not involve dose validation, dose measurement workflows, or small field dosimetry relevant to MRI-guided radiotherapy systems.
**188**	Experimental characterization and Monte Carlo simulations of the dose enhancement on the millimeter scale of PAGAT infused with gadolinium	2021	10.1016/j.radphysche m.2021.109533	Exclude	it does not focus on MRI-guided radiotherapy dose validation, small field dosimetry, or MRI-specific dose accuracy. It pertains to neutron or photon dose enhancement in gels, not MRI-based clinical dose validation workflows, so it should be excluded.
**189**	Cone beam CT based validation of neural network generated synthetic CTs for radiotherapy in the head region	2021	10.1002/mp.14987	Exclude	This study is about image-based dose calculation accuracy, not the unique physical dosimetry challenges arising from the MR-linac's magnetic field itself.
**190**	Analyzing the performance of the URT treatment planning system and URT-Linac 506C for flattening filter (FF) and flattening filter free (FFF) photon beams of Monte Carlo algorithm by use of AAPM TG 119 test cases	2021	10.18869/acadpub.ijr r.19.3.695	Exclude	he abstract makes no mention of MR-guidance or the presence of a magnetic field affecting dose delivery or dosimetry. it fails the Topic Relevance criterion for this systematic review.
**191**	Comparison between postoperative TRUS-CT fusion with MRI-CT fusion for postimplant quality assurance in prostate LDR permanent seed brachytherapy	2021	10.1016/j.brachy.202 1.03.005	Exclude	This study evaluates the agreement between TRUS-CT fusion and MRI-CT fusion for prostate brachytherapy QA, but it does not contain dose validation, dose measurement workflows, or small field dosimetry data relevant to MRI-guided radiotherapy systems.
**192**	Faraday cup for commissioning and quality assurance for proton pencil beam scanning beams at conventional and ultra-high dose rates	2021	10.1088/1361-6560/abfbf2	Exclude	it does not involve dose validation, small field dosimetry, or workflows specific to MRI-guided radiotherapy systems. Therefore, it should be excluded.
**193**	Modelling and simulation of field directed linear assembly of aerosol particles	2021	10.1016/j.jcis.2021.0 2.050	Exclude	This article focuses on modeling and simulation of field-directed linear assembly of aerosol particles under external magnetic and electric fields. The purpose is to tune the structure and fractal dimension of aggregates grown in the aerosol phase for applications in material science or environmental science.
**194**	Use of GammaPlan convolution algorithm for dose calculation on CT and cone-beam CT images	2021	10.3857/roj.2020.006 40	Exclude	This study evaluates the use of convolution dose calculation algorithms on CBCT and CT images from Gamma Knife treatments, focusing on image artifacts, Hounsfield unit variation, and plan quality metrics. While relevant to dose calculation methods, it pertains to Gamma Knife radiosurgery with CBCT

					rather than MRI-based radiotherapy, MR-Linac systems, or small field dosimetry in MRI-guided treatments. Thus, it should be excluded.
**195**	Technical Note: Three-dimensional QA of simultaneous integrated boost radiotherapy treatments by a dose-volume histogram methodology and its comparison with 3D gamma results	2021	10.1002/mp.14859	Exclude	it does not involve dose validation, small field dosimetry, or dose measurement workflows specific to MRI-guided radiotherapy systems. Therefore, it should be excluded.
**196**	Generation of clinical 177Lu SPECT/CT images based on Monte Carlo simulation with GATE	2021	10.1016/j.ejmp.2021.04.002	Exclude	his article focuses on generating simulated 177Lu SPECT/CT images using GATE Monte Carlo modelling for patient-specific dosimetry and virtual clinical trials. Although relevant to dosimetry, it does not provide quantitative dosimetric measurement data, correction factors, or studies conducted specifically on MR-Linac systems or small field dosimetry. It primarily discusses simulation and image processing rather than experimental measurements relevant to MRgRT. Therefore, it should be excluded.
**197**	Treatment planning system and beam data validation for the ZAP-X: A novel self-shielded stereotactic radiosurgery system	2021	10.1002/mp.14740	Exclude	The core of this systematic review is specifically on MR-guided radiotherapy (MRgRT) and dosimetry in the presence of magnetic fields. Therefore, this study fails the Topic Relevance criterion.
**198**	Machine QA for the Elekta Unity system: A Report from the Elekta MR-linac consortium	2021	10.1002/mp.14764	Exclude	Review Article
**199**	On the impact of absorbed dose specification, tissue heterogeneities, and applicator heterogeneities on Monte Carlo-based dosimetry of Ir-192, Se-75, and Yb-169 in conventional and intensity-modulated brachytherapy for the treatment of cervical cancer	2021	10.1002/mp.14802	Exclude	This study examines the impact of dose reporting schemes, tissue, and applicator heterogeneities on Monte Carlo-based brachytherapy dosimetry for Ir-192, Se-75, and Yb-169. While it involves dosimetric calculations and tissue heterogeneity considerations, it does not focus on MRI-guided radiotherapy, small field dosimetry, or systems specifically used in MR-Linac treatments. Its primary relevance is in brachytherapy dose calculation methodology, so it should be excluded.
**200**	First clinical implementation of GammaTile permanent brain implants after FDA clearance	2021	10.1016/j.brachy.2020.12.005	Exclude	This article discusses the clinical implementation, treatment planning, and safety considerations of GammaTile brachytherapy for brain tumors, which do not specifically involve dose validation, dose measurement workflows, or small field dosimetry in MRI-guided radiotherapy systems.
**201**	Dose-response of Fricke- and PAGAT-dosimetry gels in kilovoltage and megavoltage photon beams: Impact of LET on sensitivity	2021	10.1016/j.ejmp.2021.03.002	Exclude	it does not involve dose validation, dose measurement, or workflows specific to MRI-guided radiotherapy systems or small-field dosimetry. Therefore, it should be excluded.
**202**	Mixed Reality Visualization of Radiation Dose for Health	2021	10.1007/s10916-	Exclude	This study describes a mixed reality system with

	Professionals and Patients in Interventional Radiology		020-01700-9		Monte Carlo simulation for real-time dose visualization in interventional radiology, focusing on system development and usability, not dose validation, measurement workflows, or small field dosimetry relevant to MRI-guided radiotherapy.
**203**	X-rays minibeam radiation therapy at a conventional irradiator: Pilot evaluation in F98-glioma bearing rats and dose calculations in a human phantom	2021	10.1016/j.ctro.2021.01.001	Exclude	This study investigates minibeam radiation therapy (MBRT) in rats and performs dose calculations in a human phantom using a conventional irradiator. While relevant to novel radiotherapy approaches, it does not focus on MR-guided radiotherapy, MR-Linacs, or small field dosimetry in the context of MR-based systems. It primarily concerns x-ray MBRT techniques and preclinical models, so it should be excluded.
**204**	Dose-Volume Effects and Risk Factors for Late Diarrhea in Cervix Cancer Patients After Radiochemotherapy With Image Guided Adaptive Brachytherapy in the EMBRACE I Study	2021	10.1016/j.ijrobp.2020.10.006	Exclude	This study investigates factors associated with late diarrhea after radiochemotherapy and brachytherapy in cervical cancer patients, focusing on dose and volume effects, but it does not involve dose validation, dose measurement workflows, or small field dosimetry relevant to MRI-guided radiotherapy systems.
**205**	Absorbed dose to water determination for kilo-voltage X-rays using alanine/EPR dosimetry systems	2021	10.1016/j.radphyschem.2020.108938	Exclude	The beam qualities investigated are relevant to conventional diagnostic or superficial therapy applications, not MR-Linac systems. Therefore, it fails the Topic Relevance criterion.
**206**	Boron neutron capture therapy using cyclotron-based epithermal neutron source and borofalan (10B) for recurrent or locally advanced head and neck cancer (JHN002): An open-label phase II trial	2021	10.1016/j.radonc.2020.11.001	Exclude	This phase II trial reports on the efficacy and safety of cyclotron-based BNCT for head and neck cancers, but it does not involve dose validation, dose measurement workflows, or small field dosimetry relevant to MRI-guided radiotherapy systems.
**207**	Challenges and recommendations for magnetic hyperthermia characterization measurements	2021	10.1080/02656736.2021.1892837	Exclude	it does not involve dose validation, small field dosimetry, or dose measurement workflows specific to MRI-guided radiotherapy. Therefore, it should be excluded.
**208**	Evaluation of MRI-based MAGIC polymer gel dosimeter in small photon fields	2021	10.18869/acadpub.ijrr.14.1.59	Exclude	it does not directly align with the core criterion of Topic Relevance for MR-guided radiotherapy (MRgRT) and dosimetry in the presence of magnetic fields during irradiation.
**209**	Validation of gamma knife perfexion dose profile distribution by a modified variable ellipsoid modeling technique	2021	10.3340/jkns.2020.0186	Exclude	This study focuses on verifying Gamma Knife Perfexion dose profiles using a modified variable ellipsoid modeling technique, comparing calculations with film measurements. It pertains to gamma knife radiosurgery dose verification, not MR-guided radiotherapy or small field dosimetry on MR-Linacs. It does not provide quantitative dosimetric data relevant

					to MRgRT or small fields, so it should be excluded.
**210**	Internal dosimetry of [99mTc]NTP15-5 radiotracer for cartilage imaging in preclinical and clinical models using the GATE Monte Carlo platform	2021	10.1002/mp.14603	Exclude	This study presents detailed dosimetry calculations for a specific radiotracer using Monte Carlo simulations in preclinical and clinical models, but it does not involve dose validation, dose measurement workflows, or small field dosimetry relevant to MRI-guided radiotherapy systems.
**211**	A novel minimally invasive dynamic-shield, intensity-modulated brachytherapy system for the treatment of cervical cancer	2021	10.1002/mp.14459	Exclude	This article details the development and dosimetric evaluation of a novel MRI-compatible, needle-free brachytherapy applicator for cervical cancer, focusing on dose improvements and system design, but it does not involve dose validation, dose measurement workflows, or small field dosimetry specific to MRI-guided systems.
**212**	FLASH Investigations Using Protons: Design of Delivery System, Preclinical Setup and Confirmation of FLASH Effect with Protons in Animal Systems	2020	10.1667/RADE-20-00068.1	Exclude	t does not directly involve dose validation, dose measurement workflows, or small field dosimetry specific to MRI-guided radiotherapy processes. Therefore, it should be excluded.
**213**	100% peer review in radiation oncology: is it feasible?	2020	10.1007/s12094-020-02394-8	Exclude	While the study evaluates the feasibility and efficiency of daily peer review processes in radiation oncology, it does not involve dose validation, dose measurement workflows, or small field dosimetry specific to MRI-guided radiotherapy systems.
**214**	Radiosurgery for ventricular tachycardia: preclinical and clinical evidence and study design for a German multi-center multi-platform feasibility trial (RAVENTA)	2020	10.1007/s00392-020-01650-9	Exclude	This study outlines a multi-center feasibility trial for cardiac radiosurgery in ventricular tachycardia, focusing on safety, dose delivery, and clinical endpoints, but it does not involve dose validation, dose measurement workflows, or small field dosimetry specific to MRI-guided radiotherapy systems.
**215**	First-stage validation of a portable imageable MR-compatible water calorimeter	2020	10.1002/mp.14448	Exclude	it does not involve dosimetry in an MR-guided radiotherapy system with an active magnetic field during treatment delivery, failing the Topic Relevance criterion for this systematic review.
**216**	Predictors of urinary toxicity with MRI-assisted radiosurgery for low-dose-rate prostate brachytherapy	2020	10.1016/j.brachy.2020.06.011	Exclude	This study analyzes treatment planning factors and dosimetric predictors of urinary toxicity after MRI-assisted prostate brachytherapy, focusing on dosimetric variables and clinical outcomes rather than dose validation, measurement workflows, or small field dosimetry specific to MRI-guided systems.
**217**	Intravoxel Incoherent Motion at 7 Tesla to quantify human spinal cord perfusion: limitations and promises	2020	10.1002/mrm.28195	Exclude	It pertains to perfusion imaging and biophysical parameter estimation, not directly to radiotherapy dosimetry validation workflows. Therefore, it should be excluded.

**218**	First system for fully-automated multi-criterial treatment planning for a high-magnetic field MR-Linac applied to rectal cancer	2020	10.1080/0284186X.2020.1766697	Exclude	it fails the Dosimetric Data criterion.
**219**	Analysis of the ferrous benzoic methylthymol-blue gel dosimeter in low-dose-level measurements	2020	10.1016/j.radphyschem.2020.108943	Exclude	This study focuses on developing a low-dose-sensitive ferrous benzoic methylthymol-blue gel dosimeter and its dosimetric properties, but it does not involve dose validation, dose measurement workflows, or small field dosimetry specifically relevant to MRI-guided radiotherapy systems.
**220**	New algorithm using L1 regularization for measuring electron energy spectra	2020	10.1063/1.5144897	Exclude	It is not related to MR-guided radiotherapy (MRgRT) or its dosimetry challenges in a clinical context. Therefore, it fails the Topic Relevance criterion.
**221**	Technical Note: Taking EGSnrc to new lows: Development of egs++ lattice geometry and testing with microscopic geometries	2020	10.1002/mp.14172	Exclude	The validation focuses on general microscopic dosimetry and geometry modeling, not the specific challenges or contexts of MRgRT. Therefore, it fails the Topic Relevance criterion for this systematic review.
**222**	Radiation protection of operators and patients in a hybrid Angio-MR suite	2020	10.1016/j.ejmp.2020.04.028	Exclude	This article focuses on radiation protection in a hybrid Angio-MR suite and not on MR-guided radiotherapy (MRgRT) dosimetry.
**223**	Radiological, dosimetric and mechanical properties of a deformable breast phantom for radiation therapy and surgical applications	2020	10.1088/2057-1976/ab834a	Exclude	It focuses on anatomical and mechanical properties, making it outside the scope for MRI-based dose validation.
**224**	End-to-end delivery quality assurance of computed tomography,Äìbased high-dose-rate brachytherapy using a gel dosimeter	2020	10.1016/j.brachy.2020.02.002	Exclude	ecause its focus is on end-to-end QA verification for brachytherapy, not dose validation or small field measurement workflows specific to MRI-guided radiotherapy.
**225**	Validation of an MR-guided online adaptive radiotherapy (MRgoART) program: Deformation accuracy in a heterogeneous, deformable, anthropomorphic phantom	2020	10.1016/j.radonc.2020.02.012	Exclude	it fails the Topic Relevance criterion, as it doesn't directly address dosimetry in the presence of the magnetic field in the sense of how the magnetic field affects the radiation or its measurement.
**226**	SciFi detector and associated method for real-time determination of profile and output factor for small fields in stereotactic radiotherapy	2020	10.1002/mp.14019	Exclude	it fails the Topic Relevance criterion for this systematic review, which specifically requires studies related to dosimetry in the presence of magnetic fields for MR-guided radiotherapy.
**227**	Feasibility Study of Polymer Gel Dosimetry Using a 3D Printed Phantom for Liver Cancer Radiotherapy	2020	10.3938/jkps.76.453	Exclude	it does not involve dose validation, dose measurement workflows, or small field dosimetry specific to MRI-guided systems. Therefore, it should be excluded.
**228**	Monte Carlo dosimetry study of novel rotating MRI-compatible shielded tandems for intensity modulated cervix brachytherapy	2020	10.1016/j.ejmp.2020.02.014	Exclude	it does not involve dose validation, small field dosimetry, or workflows specific to MRI-guided radiotherapy systems. Therefore, it should be

					excluded.
**229**	Detector response in the buildup region of small MV fields	2020	10.1002/mp.13973	Exclude	It does not involve MR-guided radiotherapy (MRgRT) or the effects of magnetic fields on dosimetry.
**230**	Estimation of technical treatment accuracy in fractionated stereotactic radiosurgery	2020	10.1017/S1460396919000128	Exclude	It addresses technical treatment precision but does not involve dose validation, small field dosimetry, or MRI-specific dosimetry workflows. Therefore, it should be excluded.
**231**	Characterization of positional accuracy of a double-focused and double-stack multileaf collimator on an MR-guided radiotherapy (MRgRT) Linac using an IC-profiler array	2020	10.1002/mp.13902	Exclude	it does not directly align with the detailed "Dosimetric Data" criterion, which emphasizes "quantitative (numerical) data about dosimetric measurements (e.g., correction factors, detector response, dose accuracy)" due to the magnetic field.
**232**	Dosimetric comparison of artificial walls of bladder and rectum with real walls in common prostate IMRT techniques: Patient and Monte Carlo study	2020	10.3233/XST-190592	Exclude	t does not directly address MRI-guided small field dosimetry, MR-only workflow validation, or dose accuracy in MR-Linac systems. Therefore, it should be excluded.
**233**	MRI-assisted radiosurgery: A quality assurance nomogram for palladium-103 and iodine-125 prostate brachytherapy	2020	10.1016/j.brachy.2019.10.002	Exclude	it does not involve dose validation, dose measurement workflows, or small field dosimetry specific to MRI-guided radiotherapy systems. Therefore, it should be excluded.
**234**	Entry skin dose reduction in an inline MRI-linac using an electron contamination deflector coupled with a helium volume	2025	10.1002/mp.17923	Include	Not Excluded
**235**	Skin Dose Modeling and Measurement in a High Field In-Line MRI-Linac System	2022	10.3389/fphy.2022.902744	Include	Not Excluded
**236**	Characterizing magnetically focused contamination electrons by off-axis irradiation on an inline MRI-Linac	2022	10.1002/acm2.13591	Include	Not Excluded
**237**	Skin dose investigations on a 0.5 T parallel rotating biplanar linac-MR using Monte Carlo simulations and measurements	2024	10.1002/mp.17246	Include	Not Excluded
**238**	The role of the construction and sensitive volume of compact ionization chambers on the magnetic field-dependent dose response	2021	10.1002/mp.14994	Include	Not Excluded
**239**	Dosimetry in magnetic fields with dedicated MR-compatible ionization chambers	2020	10.1016/j.ejmp.2020.10.021	Include	Not Excluded
**240**	Experimental and Monte Carlo-based determination of magnetic field correction factors in high-energy photon fields for two ionization chambers	2023	10.1002/mp.16345	Include	Not Excluded
**241**	Calculations of magnetic field correction factors for ionization chambers in a transverse magnetic field using Monte Carlo code TOPAS	2021	10.1016/j.radphyschem.2021.109405	Include	Not Excluded
**242**	Dosimetry in 1.5¬†T MR-Linacs: Monte Carlo	2023	10.1002/mp.16082	Include	Not Excluded

	determination of magnetic field correction factors and investigation of the air gap effect				
**243**	Monte Carlo calculation of detector perturbation and quality correction factors in a 1.5 T magnetic resonance guided radiation therapy small photon beams	2021	10.1088/1361-6560/ac3344	Include	Not Excluded
**244**	Small-cavity chamber dose response in megavoltage photon beams coupled to magnetic fields	2020	10.1088/1361-6560/aba6d6	Include	Not Excluded
**245**	Monte Carlo investigation of electron fluence perturbation in MRI-guided radiotherapy beams using six commercial radiation detectors	2022	10.1088/1361-6560/ac4b36	Include	Not Excluded
**246**	The dose response of PTW microDiamond and microSilicon in transverse magnetic field under small field conditions	2021	10.1088/1361-6560/ac0f2e	Include	Not Excluded
**247**	The dose response of high-resolution diode-type detectors and the role of their structural components in strong magnetic field	2020	10.1002/mp.14535	Include	Not Excluded
**248**	Monte Carlo optimization and experimental validation of a prototype ionization chamber for accurate magnetic resonance image guided radiation therapy (MRgRT) daily output constancy measurements in solid phantoms	2022	10.1002/mp.15695	Include	Not Excluded
**249**	Determination of output correction factors in magnetic fields using two methods for two detectors at the central axis	2025	10.1088/1361-6560/adb934	Include	Not Excluded
**250**	On the correction factors for small field dosimetry in 1.5T MR-linacs	2025	10.1088/1361-6560/ada682	Include	Not Excluded
**251**	Dose-response dependencies of OSL dosimeters in conventional linacs and 1.5T MR-linacs: an experimental and Monte Carlo study	2023	10.1088/1361-6560/ad051e	Include	Not Excluded
**252**	Beam quality correction factors for ionization chambers in a 0.35 T magnetic resonance (MR)-linac ‚Äì A Monte Carlo study	2024	10.1016/j.ejmp.2024.103314	Include	Not Excluded
**253**	Implementation of a dose calculation algorithm based on Monte Carlo simulations for treatment planning towards MRI guided ion beam therapy	2020	10.1016/j.ejmp.2020.04.027	Include	Not Excluded
**254**	Absolute dosimetry of a 1.5 T MR-guided accelerator-based high-energy photon beam in water and solid phantoms using Aerrow	2020	10.1002/mp.13968	Include	Not Excluded
**255**	Influence of magnetic field on a novel scintillation dosimeter in a 1.5 T MR‚Äêlinac		10.1002/acm2.14227	Include	Not Excluded
**256**	Magnetic field quality conversion factors experimentally measured in clinical MR-linac beams for seven MR-compatible ionization chamber models	2025	10.1002/acm2.14613	Include	Not Excluded
**257**	Optical imaging method to quantify spatial dose variation due to the electron return effect in an MR-linac	2020	10.1002/mp.13954	Include	Not Excluded

**258**	Experimental determination of magnetic field quality conversion factors for eleven ionization chambers in 1.5 T and 0.35 T MR‚Äêlinac systems		10.1002/mp.16781	Include	Not Excluded
**259**	Dose perturbations at tissue interfaces during parallel linac-MR treatments: The ‚ÄúLateral Scatter Electron Return Effect‚Äù (LS-ERE)	2024	10.1002/mp.17363	Include	Not Excluded
**260**	Traceable reference dosimetry in MRI guided radiotherapy using alanine: calibration and magnetic field correction factors of ionisation chambers		10.1088/1361-6560/abd2d6	Include	Not Excluded
**261**	Small-field output factor dependence on the field size definition in MR-Linac	2025	10.1002/mp.17857	Include	Not Excluded
**262**	Surface and near-surface dose measurements at beam entry and exit in a 1.5 T MR-Linac using optically stimulated luminescence dosimeters	2020	10.1088/1361-6560/ab64b6	Include	Not Excluded
**263**	High-resolution entry and exit surface dosimetry in a 1.5¬†T MR-linac	2023	10.1007/s13246-023-01251-6	Include	Not Excluded
**264**	Dosimetric evaluation of irradiation geometry and potential air gaps in an acrylic miniphantom used for external audit of absolute dose calibration for a hybrid 1.5¬†T MR-linac system	2022	10.1002/acm2.13503	Include	Not Excluded
**265**	Uncertainty of scintillator-based field-output factor measurements in MR-Linacs with the two-channel chromatic stem removal technique	2024	10.1016/j.radmeas.2024.107163	Include	Not Excluded
**266**	Measurement of surface dose in an MR-Linac with optically stimulated luminescence dosimeters for IMRT beam geometries	2020	10.1002/mp.14185	Include	Not Excluded
**267**	Novel PENELOPE geometry subroutine for patient-Specific dosimetry in the presence of electromagnetic fields	2025	10.1016/j.radphysche m.2025.113027	Include	Not Excluded
**268**	Out-of-field dose assessment for a 1.5 T MR-Linac with optically stimulated luminescence dosimeters	2021	10.1002/mp.14839	Include	Not Excluded
**269**	MR compatible detectors assessment for a 0.35¬†T MR-linac commissioning	2024	10.1186/s13014-024-02431-8	Include	Not Excluded
**270**	Sources of out-of-field dose in MRgRT: an inter-comparison of measured and Monaco treatment planning system doses for the Elekta Unity MR-linac	2021	10.1007/s13246-021-01039-6	Include	Not Excluded
**271**	Clinical utility of Gafchromic film in an MRI-guided linear accelerator	2021	10.1186/s13014-021-01844-z	Include	Not Excluded
**272**	Monte Carlo study of small-field dosimetry for an ELEKTA Unity MR-Linac system		10.1016/j.radphysche m.2021.109867	Include	Not Excluded
**273**	Development of an EGSnrc multi-leaf collimator component module and treatment head model for a low-field MRI linear accelerator	2025	10.1002/mp.17455	Include	Not Excluded
**274**	Experimental verification the electron return effect around spherical air cavities for the MR-Linac using Monte Carlo calculation	2020	10.1002/mp.14123	Include	Not Excluded

**275**	An evaluation of the use of EBT-XD film for SRS/SBRT commissioning of a 1.5 Tesla MR-Linac system	2022	10.1016/j.ejmp.2022.02.012	Include	Not Excluded
**276**	Technical note: Characterization of a multi-point scintillation dosimetry research platform for a low-field MR-Linac	2024	10.1002/mp.17192	Include	Not Excluded
**277**	Dosimetric evaluation of off-axis fields and angular transmission for the 1.5 T MR-linac	2022	10.1088/1361-6560/ac95f3	Include	Not Excluded
**278**	Dose Calculation Accuracy of Beam Models in RadCalc for a 1.5 T MR-Linac	2024	10.3390/cancers16030526	Include	Not Excluded
**279**	Development of a collapsed cone convolution/superposition dose calculation algorithm with a mass density-specific water kernel for magnetic resonance-guided radiotherapy	2023	10.1093/jrr/rrad011	Include	Not Excluded
**280**	Effect of 0.35 T and 1.5 T magnetic fields on superficial dose in MR-guided radiotherapy of laryngeal cancer	2023	10.1016/j.ctro.2023.100624	Include	Not Excluded
**281**	Commissioning of the First MRlinac in Latin America	2024	10.4103/jmp.jmp_6_24	Include	Not Excluded
**282**	Characterization of an inorganic scintillator for small‚Äêfield dosimetry in MR‚Äêguided radiotherapy		10.1002/acm2.12948	Include	Not Excluded
**283**	Computational and experimental small field dosimetry using a commercial plastic scintillator detector for the 0.35 T MR-linac		10.1016/j.ejmp.2024.102927	Include	Not Excluded
**284**	Experimental characterization of four ionization chamber types in magnetic fields including intra-type variation		10.1016/j.phro.2023.100523	Include	Not Excluded
**285**	Comparison of Prospectively Generated Glioma Treatment Plans Clinically Delivered on Magnetic Resonance Imaging (MRI)-Linear Accelerator (MR-Linac) Versus Conventional Linac: Predicted and Measured Skin Dose	2022	10.1177/15330338221124695	Include	Not Excluded
**286**	Validation of a diode-based phantom for high temporal and spatial measurements in a 1.5 T MR-linac	2025	10.1002/acm2.14604	Include	Not Excluded
**287**	Initial Feasibility and Clinical Implementation of Daily MR-Guided Adaptive Head and Neck Cancer Radiation Therapy on a 1.5T MR-Linac System: Prospective R-IDEAL 2a/2b Systematic Clinical Evaluation of Technical Innovation	2021	10.1016/j.ijrobp.2020.12.015	Include	Not Excluded
**288**	Commissioning Intracranial Stereotactic Radiosurgery for a Magnetic Resonance-Guided Radiation Therapy (MRgRT) System: MR-RT Localization and Dosimetric End-to-End Validation	2024	10.1016/j.ijrobp.2023.08.043	Include	Not Excluded
**289**	Experimental validation of multi-fraction online adaptations in magnetic resonance guided radiotherapy	2023	10.1016/j.phro.2023.100507	Include	Not Excluded
**290**	Clinical Implementation and Initial Experience With a 1.5 Tesla MR-Linac for MR-Guided Radiation Therapy for Gynecologic Cancer: An R-IDEAL Stage 1 and 2a First in	2022	10.1016/j.prro.2022.03.002	Include	Not Excluded

	Humans Feasibility Study of New Technology Implementation				
**291**	Development and evaluation of a GEANT4‚Äêbased Monte Carlo Model of a 0.35 T MR‚Äêguided radiation therapy (MRgRT) linear accelerator		10.1002/mp.14698	Include	Not Excluded
**292**	Integration of an independent monitor unit check for high-magnetic-field MR-guided radiation therapy system		10.3389/fonc.2022.951564	Include	Not Excluded
**293**	Validation of a Monte Carlo-based dose calculation engine including the 1.5 T magnetic field for independent dose-check in MRgRT		10.1016/j.ejmp.2024.102983	Include	Not Excluded
**294**	Characterizing local dose perturbations due to gas cavities in magnetic resonance-guided radiotherapy	2020	10.1002/mp.14120	Include	Not Excluded
**295**	Performance of a newly designed end-to-end phantom compatible with magnetic resonance-guided radiotherapy systems	2021	10.1002/mp.15153	Include	Not Excluded
**296**	Dosimetric optimization and commissioning of a high field inline MRI-linac		10.3389/fonc.2020.575383	Include	Not Excluded
**297**	Real-time motion-including dose estimation of simulated multi-leaf collimator-tracked magnetic resonance-guided radiotherapy	2024	10.1002/mp.16798	Include	Not Excluded
**298**	Dosimetric evaluation of respiratory gating on a 0.35-T magnetic resonance‚Äìguided radiotherapy linac	2022	10.1002/acm2.13666	Include	Not Excluded
**299**	ART2Dose: A comprehensive dose verification platform for online adaptive radiotherapy	2024	10.1002/mp.16806	Include	Not Excluded
**300**	Treatment planning evaluation and experimental validation of the magnetic resonance-based intrafraction drift correction	2024	10.1016/j.phro.2024.100580	Include	Not Excluded
**301**	On-line adaptive MR guided radiotherapy for locally advanced pancreatic cancer: Clinical and dosimetric considerations	2020	10.1016/j.tipsro.2020.06.001	Include	Not Excluded
**302**	Impact of intrafraction motion in pancreatic cancer treatments with MR-guided adaptive radiation therapy	2023	10.3389/fonc.2023.1298099	Include	Not Excluded
**303**	Treatment plan quality during online adaptive re-planning	2020	10.1186/s13014-020-01641-0	Include	Not Excluded
**304**	An MR-only deep learning inference model-based dose estimation algorithm for MR-guided adaptive radiation therapy	2025	10.1002/mp.17759	Include	Not Excluded
**305**	Dosimetric feasibility of hippocampal avoidance whole brain radiotherapy with an MRI-guided linear accelerator	2022	10.1002/acm2.13587	Include	Not Excluded
**306**	Deep learning-based 3D in vivo dose reconstruction with an electronic portal imaging device for magnetic resonance-linear accelerators: a proof of concept study	2021	10.1088/1361-6560/ac3b66	Include	Not Excluded
**307**	In Silico Single-Fraction Stereotactic Ablative Radiation Therapy for the Treatment of Thoracic and Abdominal Oligometastatic Disease With Online Adaptive Magnetic Resonance Guidance	2021	10.1016/j.adro.2021.100652	Include	Not Excluded

**308**	Dosimetric feasibility of brain stereotactic radiosurgery with a 0.35 T MRI-guided linac and comparison vs a C-arm-mounted linac	2020	10.1002/mp.14503	Include	Not Excluded
**309**	Measurement and Incorporation of Laryngeal Motion Using cine-MRI on an MR-Linear Accelerator to Generate Radiation Therapy Plans for Early-stage Squamous Cell Cancers of the Glottis	2024	10.1016/j.adro.2024.101490	Include	Not Excluded
**310**	Evaluation of therapeutic radiographer contouring for magnetic resonance image guided online adaptive prostate radiotherapy	2023	10.1016/j.radonc.2022.109457	Include	Not Excluded
**311**	Evaluating the Hounsfield unit assignment and dose differences between CT-based standard and deep learning-based synthetic CT images for MRI-only radiation therapy of the head and neck	2024	10.1002/acm2.14239	Include	Not Excluded
**312**	Impact Assessment of Systemic Geometric Distortion in 1.5T Magnetic Resonance Imaging Simulation through Three‚Äëdimensional Geometric Distortion Phantom on Dosimetric Accuracy for Magnetic Resonance Imaging‚Äëonly Prostate Treatment Planning	2024	10.4103/jmp.jmp_62_24	Include	Not Excluded
**313**	Dosimetric evaluation of synthetic CT generated with GANs for MRI-only proton therapy treatment planning of brain tumors	2020	10.1002/acm2.12856	Include	Not Excluded
**314**	A novel anthropomorphic multimodality phantom for MRI-based radiotherapy quality assurance testing	2020	10.1002/mp.14027	Include	Not Excluded
**315**	Feasibility of Monte Carlo dropout-based uncertainty maps to evaluate deep learning-based synthetic CTs for adaptive proton therapy	2024	10.1002/mp.16838	Include	Not Excluded
**316**	Clinical feasibility of deep learning-based synthetic CT images from T2-weighted MR images for cervical cancer patients compared to MRCAT	2024	10.1038/s41598-024-59014-6	Include	Not Excluded
**317**	Prospective Clinical Feasibility Study for MRI-Only Brain Radiotherapy	2022	10.3389/fonc.2021.812643	Include	Not Excluded
**318**	Toward MR-only proton therapy planning for pediatric brain tumors: Synthesis of relative proton stopping power images with multiple sequence MRI and development of an online quality assurance tool	2022	10.1002/mp.15479	Include	Not Excluded
**319**	Determination of acceptable Hounsfield units uncertainties via a sensitivity analysis for an accurate dose calculation in the context of prostate MRI-only radiotherapy	2023	10.1007/s13246-023-01333-5	Include	Not Excluded
**320**	An end-to-end test for MR-guided online adaptive radiotherapy	2020	10.1088/1361-6560/ab8955	Include	Not Excluded

TEXT S2. Data Extraction Forms and Procedures

Data were extracted from the included studies by two independent reviewers to minimize errors. A third reviewer resolved any discrepancies. Two separate data extraction forms were developed: one for the qualitative synthesis and one for the quantitative meta-analysis as shown in Table S4 below.

Table S5. Study Extraction Forms

Column	Qualitative Data Extraction Form	Quantitative Data Extraction Form
Study ID (First Author, Year)	Yes	Yes
DOI	Yes	Yes
Research Question / Primary Objective	Yes	No
MRgRT System(s) Studied (Model, Field Strength, Orientation)	Yes	No
Detector(s) / Dosimetry System(s) Studied	Yes	No
Methodology Summary (Experimental, MC, Clinical)	Yes	No
Detector Type (e.g., Ion Chamber, Diode)	No	Yes
Detector Model (e.g., PTW 60019 microDiamond)	No	Yes
MR-Linac System (e.g., Elekta Unity)	No	Yes
B-Field Strength (T)	No	Yes
Detector Orientation (Parallel, Perpendicular, etc.)	No	Yes
Field Size (cm x cm)	No	Yes
Outcome Measure (e.g., $k_{B,Q_{msr}}$, kB,Qcli; OF)	No	Yes
Mean Value of Outcome	No	Yes
Uncertainty Type (SD, SE, 95% CI)	No	Yes
Uncertainty Value(s)	No	Yes
Source (Table #, Figure #, Text)	No	Yes

A quality assessment tool was developed based on key methodological aspects relevant to the research question. Each of the 86 included studies was assessed across five domains. A study was rated as "Low Risk," "Some Concerns," or "High Risk" for each domain. The overall risk of bias was determined by the performance across all domains.

Quality Assessment Domains:

The quality assessment of the studies focused on five key domains. The first domain, Clarity of Objective, evaluated whether the primary research question or objective was explicitly and clearly stated. Secondly, Methodological Rigor assessed if the experimental or computational setup was described with enough detail to ensure reproducibility, including specifics on detector models, phantom materials, and Monte Carlo code parameters. The third domain, Outcome Measurement, examined whether the main outcome—such as a correction factor—was defined and measured appropriately. Fourthly, the Uncertainty Analysis domain looked for a comprehensive report on both statistical and systematic uncertainties. Finally, Reporting Clarity evaluated how clearly and unambiguously the results were presented across all tables, figures, and the main text.

Summary of Quality Assessment Results:

As stated in the Table below; the overall quality of the included studies was high. Of the 86 studies, 76 (88.4%) were rated as having a "Low Risk" of bias overall. Ten studies (11.6%) were rated as having "Some Concerns," primarily due to a lack of a detailed uncertainty analysis or ambiguity in the description of the experimental setup. No studies were rated as having a "High Risk" of bias. The studies included in the meta-analysis were all rated as having a "Low Risk" of bias, as the inclusion criteria required clear reporting of outcomes and uncertaintiy.

Table S6. Overal Quality Assesments of the Included Studies

Study ID	Domain 1: Clarity of Objective	Domain 2: Methodological Rigor	Domain 3: Outcome Measurement	Domain 4: Uncertainty Analysis	Domain 5: Reporting Clarity	Overall Risk
1: Tai_2025	Low Risk	Low Risk	Low Risk	Low Risk	Low Risk	Low Risk
2: Tai_2022	Low Risk	Low Risk	Low Risk	Low Risk	Low Risk	Low Risk

3: Patterson_2022	Low Risk	Low Risk	Low Risk	Low Risk	Low Risk	Low Risk
4: Oliver_2024	Low Risk	Low Risk	Low Risk	Low Risk	Low Risk	Low Risk
5: Delfs_2021	Low Risk	Low Risk	Low Risk	Low Risk	Low Risk	Low Risk
6: Shukla_2020	Low Risk	Low Risk	Low Risk	Low Risk	Low Risk	Low Risk
7: Alissa_2023	Low Risk	Low Risk	Low Risk	Low Risk	Low Risk	Low Risk
8: Mao_2021	Low Risk	Low Risk	Low Risk	Low Risk	Low Risk	Low Risk
9: Margaroni_2023	Low Risk	Low Risk	Low Risk	Low Risk	Low Risk	Low Risk
10: Cervantes_2021	Low Risk	Low Risk	Low Risk	Low Risk	Low Risk	Low Risk
11: Cervantes_2020	Low Risk	Low Risk	Low Risk	Low Risk	Low Risk	Low Risk
12: Cervantes_2022	Low Risk	Low Risk	Low Risk	Low Risk	Low Risk	Low Risk
13: Blum_2021	Low Risk	Low Risk	Low Risk	Low Risk	Low Risk	Low Risk
14: Tekin_2020	Low Risk	Low Risk	Low Risk	Low Risk	Low Risk	Low Risk
15: Muir_2022	Low Risk	Low Risk	Low Risk	Low Risk	Low Risk	Low Risk
16: Frick_2025	Low Risk	Low Risk	Low Risk	Low Risk	Low Risk	Low Risk
17: Margaroni_2025	Low Risk	Low Risk	Low Risk	Low Risk	Low Risk	Low Risk
18: Episkopakis_2023	Low Risk	Low Risk	Low Risk	Low Risk	Low Risk	Low Risk
19: Khan_2024	Low Risk	Low Risk	Low Risk	Low Risk	Low Risk	Low Risk
20: Renaud_2020	Low Risk	Low Risk	Low Risk	Low Risk	Low Risk	Low Risk
21: Oolbekkink_N/A	Low Risk	Low Risk	Low Risk	Low Risk	Low Risk	Low Risk
22: Orlando_2025	Low Risk	Low Risk	Low Risk	Low Risk	Low Risk	Low Risk
23: Andreozzi_2020	Low Risk	Low Risk	Low Risk	Low Risk	Low Risk	Low Risk
24: Orlando_N/A	Low Risk	Low Risk	Low Risk	Low Risk	Low Risk	Low Risk

25: Steciw_2024	Low Risk	Low Risk	Low Risk	Low Risk	Low Risk	Low Risk
26: Billas_N/A	Low Risk	Low Risk	Low Risk	Low Risk	Low Risk	Low Risk
27: Das_2025	Low Risk	Low Risk	Low Risk	Low Risk	Low Risk	Low Risk
28: Kim_2020	Low Risk	Low Risk	Low Risk	Low Risk	Low Risk	Low Risk
29: Patterson_2023	Low Risk	Low Risk	Low Risk	Low Risk	Low Risk	Low Risk
30: Tyagi_2022	Low Risk	Low Risk	Low Risk	Low Risk	Low Risk	Low Risk
31: Klavsen_2024	Low Risk	Low Risk	Low Risk	Low Risk	Low Risk	Low Risk
32: Lim-Reinders_2020	Low Risk	Low Risk	Low Risk	Low Risk	Low Risk	Low Risk
33: Gayol_2025	Low Risk	Low Risk	Low Risk	Low Risk	Low Risk	Low Risk
34: Zhang_2021	Low Risk	Low Risk	Low Risk	Low Risk	Low Risk	Low Risk
35: Chea_2024	Low Risk	Low Risk	Low Risk	Low Risk	Low Risk	Low Risk
36: Baines_2021	Low Risk	Low Risk	Low Risk	Low Risk	Low Risk	Low Risk
37: Xhaferllari_2021	Low Risk	Low Risk	Low Risk	Low Risk	Low Risk	Low Risk
38: Yano_N/A	Low Risk	Low Risk	Low Risk	Low Risk	Low Risk	Low Risk
39: Etienne_2025	Low Risk	Low Risk	Low Risk	Low Risk	Low Risk	Low Risk
40: Shortall_2020 (MP)	Low Risk	Low Risk	Low Risk	Low Risk	Low Risk	Low Risk
41: Boh Lim_2022	Low Risk	Low Risk	Low Risk	Low Risk	Low Risk	Low Risk
42: Crosby_2024	Low Risk	Low Risk	Low Risk	Low Risk	Low Risk	Low Risk
43: van den Dobbelsteen_2022	Low Risk	Low Risk	Low Risk	Low Risk	Low Risk	Low Risk
44: Sung_2024	Low Risk	Low Risk	Low Risk	Low Risk	Low Risk	Low Risk
45: Ito_2023	Low Risk	Low Risk	Low Risk	Low Risk	Low Risk	Low Risk
46: Conrad_2023	Low Risk	Low Risk	Low Risk	Some Concerns	Low Risk	Some Concerns

47: Rojas-López_2024	Low Risk	Low Risk	Low Risk	Some Concerns	Low Risk	Some Concerns
48: Cusumano_N/A	Low Risk	Low Risk	Low Risk	Low Risk	Low Risk	Low Risk
49: Khan_N/A	Low Risk	Low Risk	Low Risk	Low Risk	Low Risk	Low Risk
50: Frick_N/A	Low Risk	Low Risk	Low Risk	Low Risk	Low Risk	Low Risk
51: Wang_2022 (JACMP)	Low Risk	Low Risk	Low Risk	Low Risk	Low Risk	Low Risk
52: Oolbekkink_2025	Low Risk	Low Risk	Low Risk	Low Risk	Low Risk	Low Risk
53: McDonald_2021	Low Risk	Low Risk	Low Risk	Some Concerns	Low Risk	Some Concerns
54: Mittauer_2024	Low Risk	Low Risk	Low Risk	Low Risk	Low Risk	Low Risk
55: van den Dobbelsteen_2023	Low Risk	Low Risk	Low Risk	Low Risk	Low Risk	Low Risk
56: Lakomy_2022	Low Risk	Low Risk	Low Risk	Some Concerns	Low Risk	Some Concerns
57: Khan_N/A	Low Risk	Low Risk	Low Risk	Low Risk	Low Risk	Low Risk
58: Yang_N/A	Low Risk	Low Risk	Low Risk	Low Risk	Low Risk	Low Risk
59: Ruggieri_N/A	Low Risk	Low Risk	Low Risk	Low Risk	Low Risk	Low Risk
60: Shortall_2020 (PMB)	Low Risk	Low Risk	Low Risk	Low Risk	Low Risk	Low Risk
61: Iijima_2021	Low Risk	Low Risk	Low Risk	Low Risk	Low Risk	Low Risk
62: Jelen_2020	Low Risk	Low Risk	Low Risk	Low Risk	Low Risk	Low Risk
63: Persson_2024	Low Risk	Low Risk	Low Risk	Low Risk	Low Risk	Low Risk
64: Charters_2022	Low Risk	Some Concerns	Low Risk	Some Concerns	Low Risk	Some Concerns
65: Lin_2024	Low Risk	Low Risk	Low Risk	Low Risk	Low Risk	Low Risk
66: van den Dobbelsteen_2024	Low Risk	Low Risk	Low Risk	Low Risk	Low Risk	Low Risk
67: Placidi_2020	Low Risk	Low Risk	Low Risk	Some Concerns	Low Risk	Some Concerns
68: Rusu_2023	Low Risk	Low Risk	Low Risk	Some Concerns	Low Risk	Some Concerns

69: van Timmeren_2020	Low Risk	Low Risk	Low Risk	Low Risk	Low Risk	Low Risk
70: Liu_2025	Low Risk	Low Risk	Low Risk	Low Risk	Low Risk	Low Risk
71: Graham_2022	Low Risk	Low Risk	Low Risk	Some Concerns	Low Risk	Some Concerns
72: Li_2021	Low Risk	Low Risk	Low Risk	Low Risk	Low Risk	Low Risk
73: Lee_2021	Low Risk	Low Risk	Low Risk	Low Risk	Low Risk	Low Risk
74: Slagowski_2020	Low Risk	Low Risk	Low Risk	Low Risk	Low Risk	Low Risk
75: Gupta_2024	Low Risk	Low Risk	Low Risk	Low Risk	Low Risk	Low Risk
76: Adair-Smith_2023	Low Risk	Low Risk	Low Risk	Some Concerns	Low Risk	Some Concerns
77: Singhrao_2024	Low Risk	Low Risk	Low Risk	Low Risk	Low Risk	Low Risk
78: Chaknam_2024	Low Risk	Low Risk	Low Risk	Low Risk	Low Risk	Low Risk
79: Kazemifar_2020	Low Risk	Low Risk	Low Risk	Low Risk	Low Risk	Low Risk
80: Singhrao_2020	Low Risk	Low Risk	Low Risk	Low Risk	Low Risk	Low Risk
81: Galapon_2024	Low Risk	Low Risk	Low Risk	Low Risk	Low Risk	Low Risk
82: Kim_2024	Low Risk	Low Risk	Low Risk	Low Risk	Low Risk	Low Risk
83: Lerner_2022	Low Risk	Low Risk	Low Risk	Some Concerns	Low Risk	Some Concerns
84: Wang_2022 (FRO)	Low Risk	Low Risk	Low Risk	Low Risk	Low Risk	Low Risk
85: Chourak_2023	Low Risk	Low Risk	Low Risk	Low Risk	Low Risk	Low Risk
86: Hoffmans_2020	Low Risk	Low Risk	Low Risk	Low Risk	Low Risk	Low Risk

Table S7. Comprehensive Screening Log for All 86 Included Articles

Study ID	Final Decision	Reason for Decision / Notes
1: Tai_2025	Group 2: For Qualitative Synthesis (Discussion)	This study investigates methods for skin dose reduction. It does not report a core quantitative outcome like a detector-specific correction factor or output factor with uncertainty (Criterion 1.1 & 1.2 not met). It provides valuable methodological guidance (Criterion 2.2).
2: Tai_2022	Group 2: For Qualitative Synthesis (Discussion)	This paper focuses on modeling and measuring skin dose. It does not report detector-specific correction factors required for the meta-analysis (Criterion 1.1 not met). It offers important methodological guidance (Criterion 2.2).
3: Patterson_2022	Group 2: For Qualitative Synthesis (Discussion)	This study characterizes contaminant electrons. It does not provide the core quantitative outcomes with uncertainties required for the meta-analysis (Criterion 1.1 & 1.2 not met). Its value lies in explaining foundational physics (Criterion 2.4).
4: Oliver_2024	Group 2: For Qualitative Synthesis (Discussion)	This is a skin dose investigation that compares Monte Carlo simulations with measurements. It does not report specific, uncertainty-quantified detector correction factors (Criterion 1.1 & 1.2 not met). It provides a valuable comparative analysis (Criterion 2.1).
5: Delfs_2021	Group 1: For Quantitative Synthesis (Meta-Analysis)	Criterion 1.1-1.3 Met: The study reports magnetic field-dependent dose response for a 2.2 x 2.2 cm² field, which meets the small-field criterion. Results are presented numerically with uncertainties, and all methodological context is provided.
6: Shukla_2020	Group 2: For Qualitative Synthesis (Discussion)	This study provides valuable correction factors for a 10x10 cm² reference field. As it does not report data meeting the small-field criterion (≤ 4x4 cm²) for the meta-analysis, it is included for qualitative synthesis due to its important methodological guidance on reference dosimetry (Criterion 2.2).
7: Alissa_2023	Group 2: For Qualitative Synthesis (Discussion)	The paper determines magnetic field correction factors for a 10x10 cm² reference field. Since it does not contain small-field data, it is excluded from the meta-analysis but included for its valuable contribution to the qualitative discussion on reference dosimetry.
8: Mao_2021	Group 2: For Qualitative Synthesis (Discussion)	This Monte Carlo study calculates correction factors for a 10x10 cm² reference field. It does not meet the small-field criterion for the meta-analysis but provides important methodological guidance on MC simulation techniques (Criterion 2.2).
9: Margaroni_2023	Group 1: For Quantitative Synthesis (Meta-Analysis)	Criterion 1.1-1.3 Met: The study provides Monte Carlo calculated correction factors for fields down to 3x3 cm² with uncertainties, meeting all criteria for the small-field meta-analysis.
10: Cervantes_2021	Group 1: For Quantitative Synthesis (Meta-Analysis)	Criterion 1.1-1.3 Met: This study reports quality correction factors for field widths down to 0.25 cm. The data is presented with uncertainties, and all methodological context is provided, making it ideal for the meta-analysis.
11: Cervantes_2020	Group 1: For Quantitative Synthesis (Meta-Analysis)	Criterion 1.1-1.3 Met: The study characterizes chamber response in small fields (e.g., equivalent to 1x1 cm²) and provides numerical data with uncertainties, meeting all criteria.
12: Cervantes_2022	Group 2: For Qualitative Synthesis (Discussion)	This is a Monte Carlo investigation of electron fluence perturbation. It explains foundational physics (Criterion 2.4) but does not report specific correction factors or output factors with uncertainties (Criterion 1.1 & 1.2 not met).
13: Blum_2021	Group 1: For Quantitative Synthesis (Meta-Analysis)	Criterion 1.1-1.3 Met: The study reports the dose response of two solid-state detectors in a 0.59 x 0.59 cm² field. All necessary quantitative data and methodological context are provided.
14: Tekin_2020	Group 1: For Quantitative Synthesis (Meta-Analysis)	Criterion 1.1-1.3 Met: This study investigates detector dose response in a 2.2 x 2.2 cm² field, meeting the small-field criterion. The results are presented numerically with associated uncertainties.
15: Muir_2022	Group 2: For Qualitative Synthesis (Discussion)	This work focuses on a prototype ionization chamber for QA. It does not report standard correction factors for commercial detectors (Criterion 1.1 not met). It provides valuable methodological guidance (Criterion 2.2).
16: Frick_2025	Group 1: For Quantitative Synthesis (Meta-Analysis)	Criterion 1.1-1.3 Met: The paper explicitly determines output correction factors for fields ≤ 3x3 cm². The data is presented with uncertainties in Table 3, meeting all criteria for the meta-analysis.
17: Margaroni_2025	Group 1: For Quantitative Synthesis (Meta-Analysis)	Criterion 1.1-1.3 Met: This Monte Carlo study provides correction factors for fields down to 1x1 cm². Data is presented with uncertainties, and all methodological context is included.
18: Episkopakis_2023	Group 2: For Qualitative Synthesis (Discussion)	This study reports the dose-response of OSLDs in a 10x10 cm² reference field. It does not meet the small-field criterion for the meta-analysis, but provides valuable comparative data on OSLD performance (Criterion 2.1).

19: Khan_2024	Group 2: For Qualitative Synthesis (Discussion)	The paper calculates beam quality correction factors for a 10.5x10.5 cm² field. It is excluded from the meta-analysis due to the large field size but included in the qualitative review for its methodological approach.
20: Renaud_2020	Group 2: For Qualitative Synthesis (Discussion)	This study describes a methodology for absolute dosimetry. It does not report general detector correction factors or small-field output factors with uncertainties (Criterion 1.1 & 1.2 not met).
21: Oolbekkink_N/A	Group 2: For Qualitative Synthesis (Discussion)	This is a performance validation of a novel scintillation dosimeter (Criterion 2.3). It does not provide the standardized quantitative outcomes required for the meta-analysis.
22: Orlando_2025	Group 2: For Qualitative Synthesis (Discussion)	This study reports experimentally measured quality conversion factors for a 10x10 cm² reference field. It does not meet the small-field criterion but is included in the qualitative review for its important experimental data (Criterion 2.1).
23: Andreozzi_2020	Group 2: For Qualitative Synthesis (Discussion)	This paper describes a novel optical imaging method to quantify the ERE. It does not report detector-specific correction factors (Criterion 1.1 not met). It provides excellent methodological guidance (Criterion 2.2).
24: Orlando_N/A	Group 2: For Qualitative Synthesis (Discussion)	This study reports quality conversion factors for 10x10 cm² and 10.4x10.4 cm² fields. It is excluded from the meta-analysis due to the large field sizes but is valuable for qualitative synthesis due to its extensive experimental data.
25: Steciw_2024	Group 2: For Qualitative Synthesis (Discussion)	This article provides foundational physics insights into the LS-ERE phenomenon (Criterion 2.4) but does not report the core quantitative data required for the meta-analysis.
26: Billas_N/A	Group 2: For Qualitative Synthesis (Discussion)	This study reports correction factors for a 5x5 cm² field. As this is outside our pre-defined small-field criterion of ≤ 4x4 cm², it is included for qualitative synthesis to discuss dosimetry just outside the small-field regime.
27: Das_2025	Group 1: For Quantitative Synthesis (Meta-Analysis)	Criterion 1.1-1.3 Met: This study directly reports small-field output factors for a 1.1 x 1.1 cm² field. The data is presented numerically with associated uncertainties, and all methodological context is provided.
28: Kim_2020	Group 2: For Qualitative Synthesis (Discussion)	This is a surface dosimetry application study. It provides quantitative dose data but does not report detector-specific correction factors or output factors suitable for the meta-analysis (Criterion 1.1 not met).
29: Patterson_2023	Group 2: For Qualitative Synthesis (Discussion)	This work details high-resolution surface dosimetry. While providing important quantitative measurements, it does not report the core correction factors as defined by the protocol (Criterion 1.1 not met).
30: Tyagi_2022	Group 2: For Qualitative Synthesis (Discussion)	This study evaluates the impact of air gaps in QA phantoms. It provides methodological guidance (Criterion 2.2) but does not report detector correction factors.
31: Klavsen_2024	Group 2: For Qualitative Synthesis (Discussion)	The paper focuses on quantifying the uncertainty of a measurement technique itself, which is valuable methodological guidance (Criterion 2.2), but does not report the primary outcome data for meta-analysis.
32: Lim-Reinders_2020	Group 2: For Qualitative Synthesis (Discussion)	This is a surface dose measurement study for IMRT plans. It does not report the core quantitative outcomes (correction factors, output factors) required for the meta-analysis (Criterion 1.1 not met).
33: Gayol_2025	Group 2: For Qualitative Synthesis (Discussion)	This is a methods development paper on a novel Monte Carlo subroutine. It does not report standardized correction factors for commercial detectors (Criterion 1.1 not met). It is a key paper for qualitative synthesis on advanced modeling (Criterion 2.2).
34: Zhang_2021	Group 2: For Qualitative Synthesis (Discussion)	The study assesses out-of-field dose, which is outside the scope of the in-field correction factor meta-analysis. It provides valuable methodological guidance (Criterion 2.2).
35: Chea_2024	Group 2: For Qualitative Synthesis (Discussion)	This is a detector assessment study for commissioning. It provides a comparative analysis of detectors (Criterion 2.1) but does not tabulate specific correction factors with uncertainties.
36: Baines_2021	Group 2: For Qualitative Synthesis (Discussion)	This paper investigates sources of out-of-field dose. It does not report the core in-field quantitative outcomes needed for meta-analysis but is valuable for its comparative analysis (Criterion 2.1).
37: Xhaferllari_2021	Group 2: For Qualitative Synthesis (Discussion)	The focus is on the clinical utility of Gafchromic film, validated with gamma analysis (Criterion 2.3). It does not report detector correction factors with uncertainties.
38: Yano_N/A	Group 1: For Quantitative Synthesis (Meta-Analysis)	Criterion 1.1-1.3 Met: This Monte Carlo study reports calculated output factors for various field sizes, including a 1x1 cm² field. The results are presented numerically with associated statistical uncertainties.
39: Etienne_2025	Group 2: For Qualitative Synthesis (Discussion)	This article details the development of an EGSnrc MC model. It provides methodological guidance on MC modeling (Criterion 2.2) but does not report a series of detector-specific correction factors.

40: Shortall_2020	Group 2: For Qualitative Synthesis (Discussion)	This study provides experimental verification of the ERE. It explains foundational physics (Criterion 2.4) but does not report detector-specific correction factors.
41: Boh Lim_2022	Group 2: For Qualitative Synthesis (Discussion)	This paper is an evaluation of EBT-XD film for commissioning using gamma analysis (Criterion 2.3). It does not report specific output factors with associated uncertainties.
42: Crosby_2024	Group 2: For Qualitative Synthesis (Discussion)	This technical note characterizes a novel research platform. It provides methodological guidance (Criterion 2.2) but does not report standardized factors for meta-analysis.
43: van den Dobbelsteen_2022	Group 2: For Qualitative Synthesis (Discussion)	This study provides a dosimetric evaluation of off-axis fields. It provides methodological guidance (Criterion 2.2) but does not provide the core quantitative outcomes required.
44: Sung_2024	Group 2: For Qualitative Synthesis (Discussion)	This paper validates a secondary check software using dose differences and gamma pass rates (Criterion 2.3), not detector correction factors.
45: Ito_2023	Group 2: For Qualitative Synthesis (Discussion)	This article details the development of a dose calculation algorithm. It is a key paper for qualitative synthesis on TPS algorithms (Criterion 2.2) but does not report experimental detector factors.
46: Conrad_2023	Group 2: For Qualitative Synthesis (Discussion)	This study investigates the effect of different magnetic field strengths on superficial dose. It provides a valuable comparative analysis (Criterion 2.1) but does not report the core outcomes for the meta-analysis.
47: Rojas-López_2024	Group 2: For Qualitative Synthesis (Discussion)	This is a commissioning report. While it includes output factors, the data presented in the tables do not include associated uncertainties (SD, SE, CI), thereby failing Criterion 1.2 for meta-analysis.
48: Cusumano_N/A	Group 2: For Qualitative Synthesis (Discussion)	This study characterizes a novel scintillator. It provides a comparative analysis (Criterion 2.1) but does not report factors in a format suitable for meta-analysis.
49: Khan_N/A	Group 1: For Quantitative Synthesis (Meta-Analysis)	Criterion 1.1-1.3 Met: The study reports both computational and experimental small-field output factors for a 1.1 x 1.1 cm^2 field. The results are presented numerically with associated uncertainties.
50: Frick_N/A	Group 2: For Qualitative Synthesis (Discussion)	This study provides a detailed experimental characterization of ionization chambers in a 10x10 cm^2 reference field. It does not meet the small-field criterion for the meta-analysis but offers valuable insights for qualitative synthesis.
51: Wang_2022	Group 2: For Qualitative Synthesis (Discussion)	This is a clinical study comparing treatment plans. It is valuable for its comparative clinical analysis (Criterion 2.1) but does not report detector-specific correction factors.
52: Oolbekkink_2025	Group 2: For Qualitative Synthesis (Discussion)	This paper describes the validation of a specific QA phantom (Criterion 2.3). It does not generate general detector correction factors for meta-analysis.
53: McDonald_2021	Group 2: For Qualitative Synthesis (Discussion)	This is a clinical feasibility study on workflow. It provides guidance on clinical implementation (Criterion 2.2) but not on detector dosimetry.
54: Mittauer_2024	Group 2: For Qualitative Synthesis (Discussion)	This paper details an SRS commissioning process, reporting results as gamma pass rates and positional accuracy, not the specific detector factors required for meta-analysis (Criterion 1.1 & 1.2 not met).
55: van den Dobbelsteen_2023	Group 2: For Qualitative Synthesis (Discussion)	This study is an experimental validation of a clinical process (multi-fraction adaptation). It meets Criterion 2.3 (Performance Validation) but does not report the core quantitative detector factors.
56: Lakomy_2022	Group 2: For Qualitative Synthesis (Discussion)	This R-IDEAL paper reports on clinical implementation and workflow. It does not report detector correction factors, making it suitable for qualitative review.
57: Khan_N/A	Group 1: For Quantitative Synthesis (Meta-Analysis)	Criterion 1.1-1.3 Met: This study reports calculated output factors for a range of field sizes, including a 1.7 x 1.7 cm^2 field. The results are presented with uncertainties implied by comparison to measurement.
58: Yang_N/A	Group 2: For Qualitative Synthesis (Discussion)	This paper describes an independent monitor unit (MU) check. It provides methodological guidance on an essential QA tool (Criterion 2.2) but not on fundamental detector physics.
59: Ruggieri_N/A	Group 2: For Qualitative Synthesis (Discussion)	This study validates a Monte Carlo-based dose check engine. It validates a software tool (Criterion 2.3) but does not report the detector-specific factors required.
60: Shortall_2020	Group 2: For Qualitative Synthesis (Discussion)	This study characterizes dose perturbations around gas cavities, explaining foundational physics (Criterion 2.4) and providing methodological guidance (Criterion 2.2).
61: Iijima_2021	Group 2: For Qualitative Synthesis (Discussion)	This paper describes a novel end-to-end QA phantom. It is valuable for its methodological guidance on QA (Criterion 2.2) but does not report specific detector correction factors.

62: Jelen_2020	Group 1: For Quantitative Synthesis (Meta-Analysis)	Criterion 1.1-1.3 Met: This commissioning paper reports total scatter factors (equivalent to output factors) for a 2.6 x 2.6 cm^2 field. The data points in the relevant figure have error bars, representing the associated uncertainty.
63: Persson_2024	Group 2: For Qualitative Synthesis (Discussion)	This paper validates a dose reconstruction methodology (Criterion 2.3) but does not report the core quantitative detector factors needed for the meta-analysis.
64: Charters_2022	Group 2: For Qualitative Synthesis (Discussion)	This is a dosimetric evaluation of a respiratory gating system, providing a comparative analysis of different techniques (Criterion 2.1) but not the core data for meta-analysis.
65: Lin_2024	Group 2: For Qualitative Synthesis (Discussion)	This paper describes a QA software platform, falling under Criterion 2.3 (Performance Validation) and 2.2 (Methodological Guidance). It does not report the core data for meta-analysis.
66: van den Dobbelsteen_2024	Group 2: For Qualitative Synthesis (Discussion)	This is a performance validation study (Criterion 2.3) of an intrafraction motion correction technique, valuable for the systematic review but not the meta-analysis.
67: Placidi_2020	Group 2: For Qualitative Synthesis (Discussion)	This is a clinical study on an adaptive workflow for pancreatic cancer. It reports on DVH parameters, not detector correction factors, making it suitable for qualitative synthesis (Criterion 2.2).
68: Rusu_2023	Group 2: For Qualitative Synthesis (Discussion)	This clinical study investigates the impact of intrafraction motion, highlighting a key clinical challenge (Criterion 2.2) but not reporting the core quantitative outcomes for the meta-analysis.
69: van Timmeren_2020	Group 2: For Qualitative Synthesis (Discussion)	This study compares the quality of online-adapted treatment plans, providing a valuable comparative analysis for the systematic review (Criterion 2.1).
70: Liu_2025	Group 2: For Qualitative Synthesis (Discussion)	This is a methods-development paper on a DL-based dose calculation engine. It is valuable for its novel methodology (Criterion 2.2) and discussion of future directions (Criterion 2.5).
71: Graham_2022	Group 2: For Qualitative Synthesis (Discussion)	This is a treatment planning study providing a direct comparative analysis of two technologies (Criterion 2.1) but does not report the detector factors required for meta-analysis.
72: Li_2021	Group 2: For Qualitative Synthesis (Discussion)	This study presents a DL-based method for 3D in vivo dose reconstruction. It offers methodological guidance on a novel QA technique (Criterion 2.2) but does not report reference dosimetry data.
73: Lee_2021	Group 2: For Qualitative Synthesis (Discussion)	This is an "in silico" treatment planning study. It provides methodological guidance on an advanced treatment technique (Criterion 2.2) but does not report measured detector data.
74: Slagowski_2020	Group 2: For Qualitative Synthesis (Discussion)	This study compares the dosimetric feasibility of brain SRS between an MR-linac and a conventional linac. It is an excellent example of a comparative analysis (Criterion 2.1).
75: Gupta_2024	Group 2: For Qualitative Synthesis (Discussion)	The focus is on measuring laryngeal motion to inform treatment planning. It provides methodological guidance on motion management (Criterion 2.2) but does not report detector physics data.
76: Adair-Smith_2023	Group 2: For Qualitative Synthesis (Discussion)	This paper evaluates a clinical workflow (radiographer contouring). It is a study on clinical practice (Criterion 2.2) and does not report detector correction factors.
77: Singhrao_2024	Group 2: For Qualitative Synthesis (Discussion)	This is a performance validation of an sCT algorithm (Criterion 2.3) and does not report detector correction factors.
78: Chaknam_2024	Group 2: For Qualitative Synthesis (Discussion)	This study assesses the dosimetric impact of MRI geometric distortion. It provides methodological guidance on an important QA aspect (Criterion 2.2) but not detector correction factors.
79: Kazemifar_2020	Group 2: For Qualitative Synthesis (Discussion)	This is a dosimetric evaluation of sCT for MRI-only proton therapy. It is included in Group 2 for its general methodological relevance to sCT (Criterion 2.3), but the modality difference will be noted.
80: Singhrao_2020	Group 2: For Qualitative Synthesis (Discussion)	This paper describes a novel anthropomorphic phantom for QA. It provides methodological guidance (Criterion 2.2) but does not report general detector correction factors.
81: Galapon_2024	Group 2: For Qualitative Synthesis (Discussion)	This study evaluates uncertainty maps for sCTs for adaptive proton therapy. Included in Group 2 for its general methodological relevance to sCT QA (Criterion 2.2).
82: Kim_2024	Group 2: For Qualitative Synthesis (Discussion)	This is a comparative analysis (Criterion 2.1) and performance validation (Criterion 2.3) of sCT generation methods, not a report on detector correction factors.
83: Lerner_2022	Group 2: For Qualitative Synthesis (Discussion)	This is a prospective clinical feasibility study of an MRI-only workflow. It provides methodological guidance on clinical implementation (Criterion 2.2) but not the core quantitative detector data.

84: Wang_2022	Group 2: For Qualitative Synthesis (Discussion)	This study aims to generate sRPSP images for proton therapy. Included in Group 2 for its methodological guidance on sCT generation and QA (Criterion 2.2).
85: Chourak_2023	Group 2: For Qualitative Synthesis (Discussion)	This paper performs a sensitivity analysis for sCTs, providing methodological guidance (Criterion 2.2), but does not report measured detector correction factors.
86: Hoffmans_2020	Group 2: For Qualitative Synthesis (Discussion)	This is a performance validation study (Criterion 2.3) of an end-to-end test using a deformable phantom and film dosimetry, valuable for the systematic review.

TEXT S3. Statistical Calculations and Mathematical Framework

Random-Effects Meta-Analysis Model:

A random-effects meta-analysis model was used to synthesize the detector-specific correction factors ($k_{B,Q}$) and output factors. This model assumes that the true effect size varies between studies and estimates the mean of this distribution of effects.

Effect Size and Standard Error:

For each study *i*, the effect size is the reported correction factor, k_i. The standard error, se_i, was calculated from the reported uncertainty. If standard deviation (sd_i) was reported, $se_i = sd_i$.

Heterogeneity Assessment:

Heterogeneity was assessed using Cochran's *Q* statistic:

$$Q = \sum_{i=1}^{N} w_i(k_i - \bar{k}_{FE})^2$$

where *N* is the number of data points, $w_i = 1/se_i^2$ is the within-study weight, and $\bar{k}_{FE}$ is the pooled estimate from a fixed-effect model. The between-study variance, τ^2, was estimated using the DerSimonian and Laird method:

$$\tau^2 = Q - (N - 1) / \sum w_i - \sum w_i^2/\sum w_i$$

The I^2 statistic, which describes the percentage of total variation across studies due to heterogeneity rather than chance, was calculated as:

$$I^2 = (Q - (N - 1))/Q \times 100\%$$

Pooled Estimate (Random-Effects):

The pooled mean correction factor, $\bar{k}_{RE}$, was calculated using random-effects weights, w_i^*, which incorporate both within-study and between-study variance:

$$w_i^* = 1/(se_i^2 + \tau^2)$$

$$\bar{k}_{RE} = \sum(w_i^* \times k_i)/\sum w_i^*$$

Confidence Interval:

The standard error of the pooled mean was calculated as:

$$SE(\bar{k}_{RE}) = \sqrt{(1/\sum w_i^*)}$$

The 95% confidence interval (CI) was then calculated as:

$$95\%\ CI = \bar{k}_{RE} \pm 1.96 \times SE(\bar{k}_{RE})$$

Table S8. Detailed Summary of Qualitatively Synthesized Studies

Study	Main Theme	Key Finding / Argument (Detailed)
1: Tai_2025	Skin Dose Mitigation	This study proposes a method to reduce the significant entrance skin dose caused by magnetically focused electron contamination in high-field inline MRI-linacs. Using Monte Carlo simulations and experimental measurements, it demonstrates that a bespoke permanent magnet electron deflector combined with a helium-filled volume can reduce the peak surface dose by approximately 60%. This combined approach is shown to be superior to using a simple water-equivalent bolus, as it mitigates the dose hotspot while preserving a degree of skin-sparing effect.
2: Tai_2022	Skin Dose Modeling	The study develops and validates a detailed Geant4 Monte Carlo model to accurately simulate the entrance skin dose in the 1 T Australian inline MRI-Linac. The model confirms that focused electron contamination is the primary source, leading to surface dose increases of up to 320% for a 10x10 cm^2 field. The simulation results showed good agreement (±10%) with experimental MOSkin™ measurements, providing a robust framework for future treatment planning and skin dose estimation.
3: Patterson_2022	Contaminant Electron Characterization	This work characterizes the magnetically focused contaminant electrons in an inline MRI-Linac by deliberately irradiating off-axis. Experimental measurements and Monte Carlo simulations showed that by offsetting the field, the electron contamination (focal spot size ~2.5 mm FWHM) can be spatially separated from the primary photon beam. This significantly reduces the in-field surface dose and restores the skin-sparing effect, an outcome not achievable with other methods like bolus at isocenter.
4: Oliver_2024	Skin Dose in Parallel MR-Linac	This study investigates skin dose in a 0.5 T parallel-orientation linac-MR using Monte Carlo simulations and film measurements. It finds that the magnetic field enhances both entrance dose (due to electron spiraling impeding lateral scatter) and exit dose (due to a form of ERE). The dose increase is field-size dependent and can be significant (e.g., D_0.2cc increased from 34% to 72% for a 20x20 cm^2 field), highlighting the need to account for these effects in treatment planning, for instance by avoiding oblique beams.
5: Shukla_2020	Reference Dosimetry in Magnetic Fields	This study investigates the feasibility of using dedicated MR-compatible Exradin ionization chambers for reference dosimetry. Through Monte Carlo simulations (EGSnrc) and experimental validation up to 1.1 T, it reports magnetic field correction factors. It concludes that for a transverse field, chamber response can increase by up to 8.6%, while for a parallel field, the effect is negligible. The study also confirms that polarity and recombination effects are not altered by the magnetic field, providing critical data for establishing dosimetry protocols.
76 Alissa_2023	Reference Dosimetry Correction Factors	This paper provides experimentally and Monte Carlo-determined correction factors (k_B,Q) for two Sun Nuclear ionization chambers (SNC125c and SNC600c) in magnetic fields up to 1.5 T. The study confirms that the magnitude of the correction strongly depends on chamber volume and orientation relative to the field. Good agreement between measurements and simulations was achieved, and the results provide applicable correction factors for clinical reference dosimetry in MR-Linacs.
7: Mao_2021	MC Calculation of Correction Factors	Using the TOPAS Monte Carlo code, this study calculates magnetic field correction factors (k_B) for four common cylindrical ionization chambers in a 1.5 T transverse field. It systematically investigates the effect of chamber orientation, finding that corrections are small (<2.1%) in the parallel orientation but can be significant (up to 5.5% at 1.0 T) in the perpendicular orientation. The study validates its simulation method and provides reference data for various field strengths.
8: Cervantes_2022	Detector Perturbation	This Monte Carlo study provides a deep physical insight into detector response by evaluating how a 1.5 T magnetic field perturbs the electron fluence spectra within six different commercial detectors. It concludes that the magnetic field significantly alters the fluence, especially for low-energy electrons. Detectors with higher density and high-Z extracameral components exhibit more significant perturbations, explaining the varying responses observed in different detector types.
9: Muir_2022	Novel Detector Design for QA	To address measurement inaccuracies in solid phantoms caused by air gaps, this study optimizes and validates a

		prototype ionization chamber with a thick brass wall (1.1 mm) for daily output constancy checks. Monte Carlo simulations and experimental tests show the prototype's response is independent of phantom material and rotational position (<0.3% variation), mitigating the air gap effect and allowing for more accurate and streamlined QA procedures.
10: Episkopakis_2023	OSLD Characterization	This work characterizes the dose-response of nanoDot OSLDs in a 1.5 T MR-Linac. It finds that while dose-response linearity and signal fading are largely unaffected by the magnetic field, the angular (orientation) dependence is significantly more pronounced. It determines the necessary k_B,Q factors (up to 6.4%) for cardinal orientations but suggests that calibrating and measuring in the axial orientation (perpendicular to B-field) makes the correction negligible.
11: Khan_2024	Correction Factors in Low-Field MR-Linac	This Monte Carlo study directly calculates beam quality correction factors (k_Q) for four Exradin cylindrical ionization chambers in a 0.35 T MR-linac. The study finds that the magnetic field dependence is <1% and the overall correction is <2% when the chamber is oriented parallel to the magnetic field, strongly recommending this orientation for reference dosimetry to minimize uncertainties. An asymmetry in response of up to 8.3% was noted for some chambers in the orthogonal orientation.
12: Renaud_2020	Absolute Dosimetry Methodology	This study introduces and validates the "Aerrow," a probe-format graphite calorimeter, for absolute dosimetry in a 1.5 T MR-linac. The results show that the calorimeter's measurements agree with reference ionization chambers in the absence of a B-field or in a parallel orientation. However, it highlights statistically significant differences (2-4%) with ICs in the perpendicular orientation, suggesting calorimetry provides a more accurate and direct method for absolute dose measurement in MR-Linacs.
13: Oolbekkink_N/A	Scintillation Dosimetry	This study investigates a novel scintillation dosimeter's performance in a 1.5 T MR-linac. It finds that the magnetic field has a significant but correctable influence on the detector's light yield and that the Cherenkov-to-scintillation ratio is also affected. After applying corrections, the scintillator is shown to be a suitable tool for relative dosimetry, including output factor measurements, in an MRgRT environment.
14: Orlando_2025	Experimental Conversion Factors	This study provides a critical dataset of experimentally measured magnetic field quality conversion factors (k_B,Q) for seven different MR-compatible ionization chamber models in both 1.5 T Elekta Unity and 0.35 T ViewRay MRIdian systems. The results provide an important reference for upcoming dosimetry protocols and demonstrate nearly equivalent response between MR-compatible chambers and their conventional counterparts, validating their clinical use.
15: Andreozzi_2020	ERE Quantification	This paper demonstrates a novel optical imaging method using Cherenkov emission to visualize and quantify the Electron Return Effect (ERE) at tissue-air interfaces in a 0.35 T MR-linac. The high-resolution optical data revealed appreciable differences in both the magnitude (up to 20%) and spatial distribution of the ERE compared to the dose predicted by the clinical treatment planning system, highlighting the need for advanced methods to validate Monte Carlo algorithms in these complex regions.
16: Orlando_N/A	Experimental Conversion Factors (Multi-System)	Expanding on previous work, this study experimentally determines magnetic field quality conversion factors for eleven different ionization chamber models in both 1.5 T and 0.35 T MR-linac systems. The comprehensive dataset serves as a crucial reference for clinical physicists, validating the performance of a wide range of detectors and supporting the establishment of robust, multi-platform reference dosimetry protocols for MRgRT.
17: Steciw_2024	Novel ERE Phenomenon	This study describes a previously unreported dosimetric perturbation, the "Lateral Scatter Electron Return Effect" (LS-ERE), present in parallel-orientation MR-Linacs. It demonstrates through Monte Carlo simulations that laterally scattered electrons can return to the surface at tissue-air interfaces, causing asymmetric dose elevations outside the primary field. This effect is most pronounced at lower magnetic field strengths and highlights a unique physics challenge for TPS modeling.
18: Billas_N/A	Traceable Reference Dosimetry	This study establishes alanine as a suitable transfer standard for traceable reference dosimetry in MR-guided radiotherapy, as its response in a magnetic field is well-characterized and requires only a small correction. It then

		uses this standard to determine magnetic field correction factors for various ionization chambers in a 5x5 cm² field, providing a robust, traceable dataset for clinical physics.
19: Kim_2020	Surface Dosimetry Application	This study demonstrates the utility of optically stimulated luminescence dosimeters (OSLDs) for measuring surface and near-surface dose at both beam entry and exit in a 1.5 T MR-Linac. The measurements successfully quantified the reduced entry dose (skin-sparing) and the significant exit dose enhancement from the Electron Return Effect, showing close agreement with the Monaco TPS and validating OSLDs for in-vivo surface dosimetry.
20: Patterson_2023	High-Resolution Surface Dosimetry	Using a high-resolution MOSkin™ detector, this study accurately measures the steep dose gradients at beam entry and exit surfaces on a 1.5 T MR-linac. The results are compared with Geant4 simulations, the Monaco TPS, and EBT-3 film, validating the MOSkin™ as a suitable tool for characterizing these complex regions and providing benchmark data for the electron return effect and entry skin dose across various field sizes.
21: Tyagi_2022	QA & Phantom Dosimetry	This study investigated the dosimetric impact of potential air gaps around detectors within an acrylic miniphantom used for external dose audits on a 1.5 T MR-Linac. Both OSLD measurements and Monte Carlo simulations confirmed that air gaps as small as 1-2 mm cause significant dose over-response (simulated at +13.3% and +27.9%, respectively) due to the electron return effect. The study concludes that extreme care must be taken to eliminate air around dosimeters in solid phantoms to avoid substantial errors during QA procedures in high magnetic fields.
22: Klavsen_2024	Uncertainty in Scintillator Dosimetry	This work systematically analyzes and quantifies the sources of uncertainty in plastic scintillator-based field-output factor measurements in MR-Linacs. It validates a two-channel chromatic stem removal technique and develops a robust measurement protocol, demonstrating that with proper corrections, scintillators can provide highly accurate and traceable measurements in a clinical MRgRT environment.
23: Lim-Reinders_2020	Surface Dose in IMRT	This study uses OSLDs on an anthropomorphic phantom to perform the first experimental measurements of surface dose for clinical IMRT plans delivered on a 1.5 T MR-Linac. The results show that surface doses are approximately half the prescription dose for multi-beam plans and that increasing the number of beams generally lowers the skin dose, providing crucial validation data for a potentially dose-limiting factor in MRgRT.
24: Gayol_2025	Advanced MC Modeling	This paper presents a novel geometry subroutine, voxgeom, for the PENELOPE Monte Carlo code. This development allows for detailed, patient-specific dosimetry simulations in complex voxelized geometries while fully accounting for the influence of external electromagnetic fields, overcoming a key limitation of previous tools and improving the accuracy of research in MRgRT.
25: Zhang_2021	Out-of-Field Dose	This study provides an assessment of out-of-field dose for a 1.5 T MR-Linac using OSLDs. It finds that the MR-Linac delivers a relatively higher out-of-field dose compared to a conventional linac on both the surface and at internal points. Furthermore, it shows the Monaco TPS has variable accuracy in this region, overestimating at low doses and underestimating at intermediate doses, suggesting caution is needed for OARs near the field edge.
26: Chea_2024	Detector Assessment for Commissioning	This study presents a comprehensive assessment of ten different MR-compatible detectors for commissioning a 0.35 T MR-linac. It concludes that while the low magnetic field reduces perturbations, detector choice remains critical. The PTW 31010 was found reliable for absolute dose, while the PTW 60019 microDiamond showed the best agreement for relative dosimetry across the full range of field sizes.
27: Baines_2021	Out-of-Field Dose Sources	By comparing measurements with TPS calculations for the Elekta Unity, this study identifies MLC transmission and patient scatter as the dominant sources of out-of-field dose. It also quantifies the dose from the Electron Streaming Effect (ESE) caused by the anterior imaging coil and notes that the Monaco TPS underestimates this effect, highlighting a potential area for model improvement.
28: Xhaferllari_2021	Film Dosimetry Utility	This study comprehensively evaluates Gafchromic EBT3 and EBT-XD film for QA in a 0.35 T MR-linac. It finds that the magnetic field has a negligible effect on the film's response and crystalline structure. High gamma pass rates for both open fields and patient-specific QA demonstrate that radiochromic film is a reliable and accurate high-resolution dosimeter for MRgRT.
29: Etienne_2025	MC Model Development	This paper details the development and validation of a new EGSnrc/BEAMnrc component module for the double-

		focused MLC of a low-field (0.35 T) MRI-linac. The resulting validated treatment head model provides a crucial, vendor-independent tool for researchers to perform advanced Monte Carlo simulations and investigate the physics of this specific MRgRT system.
30: Shortall_2020	ERE Verification	This work provides an important experimental verification of the Electron Return Effect (ERE) around spherical air cavities, which is a critical issue for patient safety in MRgRT. Using Gafchromic film measurements inside custom phantoms, the study demonstrates that the Monaco treatment planning system accurately calculates the dose perturbations caused by ERE, with gamma pass rates >95% (3%/3mm), validating its use for clinical planning.
31: Boh Lim_2022	Film for SRS/SBRT Commissioning	This study evaluates the use of EBT-XD film for the commissioning of stereotactic treatments on a 1.5 T MR-Linac. It demonstrates that with proper protocols, including adding water to minimize air gaps and the ERE, film provides accurate output factor and profile measurements. An end-to-end test confirmed its utility, validating film as a valuable tool for commissioning complex, small-field deliveries on an MR-Linac.
32: Crosby_2024	Novel Dosimetry Platform	This technical note characterizes a commercial multi-point plastic scintillation dosimetry platform for use in a low-field (0.35 T) MR-Linac. The system demonstrated high repeatability, linearity, and minimal angular dependence. Its time-resolved, multi-point capability showed considerable promise for advanced applications like verifying gated treatments and tracking delivery efficiency, addressing a key QA need in MRgRT.
33: van den Dobbelsteen_2022	Off-Axis Dosimetry	This study provides a comprehensive dosimetric validation of the GPUMCD dose calculation algorithm used in the Elekta Unity TPS, specifically for off-axis fields and angular transmission through the gantry and couch. It confirms that the algorithm is highly accurate (point doses within 1.7%, penumbra DTA < 0.7 mm) for fields 2x2 cm^2 and larger, providing essential commissioning data and confidence in the TPS for clinical use.
34: Sung_2024	Secondary Dose Calculation Validation	This work validates the accuracy of RadCalc, an independent dose verification software, for use with a 1.5 T MR-Linac. It demonstrates that with proper beam modeling, RadCalc provides clinically acceptable dose calculation accuracy for online adaptive plans, making it a reliable and effective tool for secondary checks and patient-specific QA in an adaptive workflow.
35: Ito_2023	Dose Calculation Algorithm Development	This paper describes the development of a novel collapsed cone convolution/superposition (CCC) dose calculation algorithm specifically for MRgRT. By using a mass density-specific water kernel, the CCC_MR algorithm improves dose calculation accuracy in heterogeneous regions (like bone and lung) compared to standard density scaling methods, showing good agreement with Monte Carlo simulations.
36: Conrad_2023	Superficial Dose Comparison	This study compares the superficial dose for laryngeal cancer plans on 0.35 T and 1.5 T MR-Linacs. It demonstrates that the Electron Return Effect on both systems provides sufficient dose coverage for superficially located targets, effectively acting like a bolus. Notably, the higher 1.5 T field results in a more pronounced dose reduction at the skin surface, indicating better skin sparing compared to the 0.35 T system.
37: Rojas-López_2024	Commissioning Experience	This paper details the commissioning process of the first MR-linac in Latin America, providing a valuable workflow and reference dataset. A key finding for this review is that while output factors were measured, they were reported without associated uncertainties, which highlights a common issue in published data that prevents its inclusion in a formal meta-analysis and underscores the need for standardized uncertainty reporting.
38: Cusumano_N/A	Scintillator Characterization	This study characterizes an inorganic scintillator (GAGG:Ce) for small-field dosimetry in MRgRT. It finds the detector has favorable properties such as dose linearity and minimal energy dependence. However, it requires a stem signal correction (for Cherenkov light) and exhibits a small magnetic field dependence, providing important characterization data for this promising detector type.
39: Frick_N/A	Experimental Detector Characterization	This work provides a detailed experimental characterization of four types of ionization chambers in magnetic fields under reference conditions (10x10 cm^2). A key contribution is the investigation of intra-type variations (differences between detectors of the same model), providing valuable data for understanding the overall uncertainty budget in MR-Linac reference dosimetry.
40: Wang_2022	Clinical Plan Comparison	In a prospective study of glioma patients, this work compares treatment plans on an MR-Linac versus a

		conventional linac. It finds that while MR-Linac plans had higher mean skin doses, these doses were more accurately predicted by the Monte Carlo-based TPS compared to the convolution-based algorithm for the conventional linac. This validates the accuracy of the MR-Linac TPS in predicting complex surface dose effects.
41: Oolbekkink_2025	Diode Phantom Validation	This study validates the Delta4 Phantom+ MR, a diode array phantom, for time-resolved dosimetry in a 1.5 T MR-linac. It demonstrates the system's dose reproducibility and dose rate independence, confirming its suitability for QA of dynamic deliveries such as beam gating and sliding window IMRT, fulfilling a need for advanced QA tools in MRgRT.
42: McDonald_2021	Clinical Workflow (H&N)	This paper reports on the initial clinical implementation of daily MR-guided adaptive radiotherapy for head and neck cancer. Following the R-IDEAL framework, it demonstrates that an online adapt-to-position (ATP) workflow is safe and clinically feasible. The study details the entire process, providing crucial insights into the workflow, time requirements, and dosimetric consistency for this complex treatment site.
43: Mittauer_2024	SRS Commissioning	This study provides a comprehensive methodology for commissioning an intracranial stereotactic radiosurgery (SRS) program on the ViewRay MRIdian system. It benchmarks the end-to-end localization accuracy (<1 mm) and dosimetric accuracy (<1% absolute dose difference) using a custom phantom, confirming the system meets the stringent requirements for SRS delivery.
44: van den Dobbelsteen_2023	Online Adaptation Validation	This study experimentally validates the entire multi-fraction online adaptive workflow of an MR-linac. Using a deformable phantom with film, it confirms that the system can accurately account for inter-fractional translations, rotations, and body shape modifications, and correctly deliver the accumulated dose over a five-fraction course, demonstrating the dosimetric integrity of the adaptive process.
45: Lakomy_2022	Clinical Workflow (Gyn)	Reporting on the first clinical experience using a 1.5 T MR-Linac for gynecologic cancer, this study demonstrates the feasibility of both "adapt to position" (ATP) and the more complex "adapt to shape" (ATS) workflows. It provides data on treatment times and QA pass rates, confirming the system is a reliable tool for this patient population.
46: Yang_N/A	MU Check Integration	This paper describes the development, integration, and validation of an independent monitor unit (MU) check for a high-field MRgRT system. This tool is a critical safety component for online adaptive workflows where plans are created and delivered within a short timeframe, and the study confirms its accuracy and importance.
47: Ruggieri_N/A	MC Dose Check Validation	This study validates a Monte Carlo-based dose calculation engine (SciMoCa) for use as an independent dose check in a 1.5 T MRgRT system. The results, showing high gamma pass rates against the clinical TPS, confirm its suitability for verifying complex adaptive plans, providing an essential layer of quality assurance.
48: Shortall_2020	Dose Perturbations by Gas	This study characterizes the dose perturbations around gas cavities in MRgRT and proposes a novel analytical model to predict them. It finds that both the Electron Return Effect (ERE) and attenuation contribute to the dose changes. The proposed model allows for a rapid evaluation of the dosimetric impact of unplanned gas, which is crucial for real-time adaptive decision-making.
49: Iijima_2021	Novel E2E Phantom	This paper describes a newly designed, hexagonal, MR-compatible end-to-end (E2E) phantom. The design simplifies the QA process by allowing for image-matching, irradiation field, and composite E2E tests to be performed with a single phantom, which was previously unfeasible and required multiple devices.
50: Persson_2024	Real-Time Dose Estimation	This work presents a proof-of-concept software workflow for real-time, motion-inclusive dose estimation for MLC-tracked MRgRT. By combining motion management software with a fast Monte Carlo dose engine, it demonstrates the feasibility of reconstructing the delivered dose during treatment, a key step towards enabling real-time intra-fraction adaptation.
51: Charters_2022	Respiratory Gating Evaluation	This study evaluates the dosimetric accuracy of respiratory gating on a 0.35 T MR-linac, specifically comparing different tracking algorithms available after a system upgrade. It finds that the choice of algorithm significantly impacts gating performance, indicating that it must be carefully selected based on whether the target is rigid or deforming to ensure accuracy.
52: Lin_2024	Online ART QA Platform	This paper introduces ART2Dose, a comprehensive and automated software platform designed for secondary dose

		verification in online adaptive radiotherapy. The platform significantly improves efficiency by reducing the QA check time by 3–4 minutes per plan compared to commercial software, addressing a major bottleneck in the time-sensitive online ART workflow.
53: van den Dobbelsteen_2024	Intrafraction Motion Correction	This study validates the Intrafraction Drift Correction (IDC) technique, a fast method to adapt plans for prostate cancer by repositioning segments in response to motion. The planning evaluation and experimental validation show that this simpler, faster method provides dosimetric results consistent with more complex and time-consuming re-optimization strategies.
54: Placidi_2020	Clinical ART for Pancreatic Cancer	This study demonstrates the feasibility and significant dosimetric benefit of an online adaptive workflow for locally advanced pancreatic cancer. It found that online adaptation was necessary and advantageous in nearly 70% of fractions, significantly improving target coverage (mean PTV V95% increased by 10.8%) and OAR sparing compared to applying the reference plan to the daily anatomy.
55: Rusu_2023	Intrafraction Motion in Pancreatic Cancer	This study highlights a key challenge in MR-guided ART: significant intrafraction motion of gastrointestinal organs occurs during the long workflow. This motion can lead to violations of OAR dose constraints that were met in the freshly adapted plan. However, the study concludes that despite this degradation, adaptation still provides a net dosimetric benefit compared to using a non-adapted plan.
56: van Timmeren_2020	Plan Quality in Online ART	This study compares the quality of online-adapted plans to their reference plans across five different treatment sites. It finds that the dosimetric quality is generally comparable, with OAR doses often decreasing (e.g., rectum dose in prostate plans). However, it notes that dose spillage outside the target (Ring Dmean) can be slightly increased in the adapted plans, a factor to consider during plan evaluation.
57: Liu_2025	AI-based Dose Calculation	This study develops a novel deep learning model that functions as an MR-only dose calculation engine. The model directly maps beam fluence to dose distribution, completely bypassing the need for a synthetic CT or ray-tracing. The high accuracy against Monte Carlo demonstrates its potential to significantly speed up the online adaptive workflow.
58: Graham_2022	HA-WBRT Feasibility	This treatment planning study demonstrates the dosimetric feasibility of delivering hippocampal-avoidance whole-brain radiotherapy (HA-WBRT) on a 0.35 T MRI-guided linac. The MRgRT plans were able to meet all clinical goals from the RTOG 0933 trial and were comparable in quality to plans from a conventional C-Arm linac.
59: Li_2021	AI-based In-Vivo Dosimetry	This study develops a novel deep learning framework to reconstruct the 3D in-vivo dose from 2D EPID portal dose images acquired on an MR-Linac. The method is fast (<3 seconds per beam) and accurate (gamma >97%), and importantly, the model implicitly learns to account for the electron return effect, offering a promising tool for online dosimetric verification.
60: Lee_2021	In-Silico Feasibility of SBRT	This computational ("in silico") study demonstrates that single-fraction, online adaptive MR-guided SABR is dosimetrically feasible for challenging thoracic and abdominal oligometastases. It shows that on-table re-optimization is crucial, as it significantly improves target coverage and eliminates OAR dose violations that would have occurred due to daily anatomical changes.
61: Slagowski_2020	Brain SRS Feasibility	This study compares the feasibility of brain SRS between a 0.35 T MR-linac and a conventional C-arm linac. It concludes that the MRL can produce clinically acceptable SRS plans for smaller lesions (≤2.25 cm). However, for larger targets, the non-coplanar beam arrangements possible on a C-arm linac are superior for sparing normal brain tissue.
62: Gupta_2024	Motion Management (Larynx)	Using cine-MRI, this study quantifies laryngeal motion and finds that infrequent, short-duration swallows are the largest source of motion. It concludes that accounting only for resting motion in an ITV is sufficient for planning, which allows for smaller, more conformal treatment volumes that can significantly spare the carotid arteries when using IMRT/VMAT techniques.
63: Adair-Smith_2023	Clinical Workflow (Contouring)	This study validates the practice of role extension where trained therapeutic radiographers perform online contouring for adaptive prostate radiotherapy. An interobserver variability study showed that radiographer contours

		were comparable to clinician contours (median DSC of 0.92) and produced clinically acceptable plans, supporting this practice to improve clinical efficiency.
64: Singhrao_2024	sCT Evaluation (H&N)	This study evaluates a deep learning synthetic CT (sCT) generation method for head and neck cancer. It finds statistically significant HU differences in bone and air regions compared to real CT, but these differences did not translate into clinically significant dosimetric errors, with gamma pass rates >95% (3%/3mm) and average DVH metric differences <3%.
65: Chaknam_2024	MRI Geometric Distortion	This study assesses the dosimetric impact of MRI systemic geometric distortion (SGD) for prostate MRI-only planning. It finds that while SGD exists and increases with distance from isocenter, its impact on dosimetric accuracy for the PTV was mostly under 2%. However, larger errors were seen in deformable OARs, underscoring the necessity of including SGD assessment in QA programs.
66: Kazemifar_2020	sCT for Proton Therapy (Brain)	This work demonstrates the feasibility of using a GAN-based deep learning method to generate sCT from MRI for MRI-only proton therapy planning. The sCT-based proton plans showed excellent dosimetric accuracy, with mean DVH differences <0.5% for the CTV compared to plans based on real CT, enabling an MRI-only proton workflow.
67: Singhrao_2020	Novel Multimodality Phantom	This paper describes the development of a novel, 3D-printed, anthropomorphic pelvis phantom with materials that mimic human tissue contrast on both CT and MRI. This allows for a comprehensive end-to-end validation of an MRI-only workflow, including sCT generation and dose delivery, by comparing it directly to the CT-based standard using the same phantom.
68: Galapon_2024	sCT Uncertainty Maps for Proton Therapy	This study demonstrates that Monte Carlo dropout-based uncertainty maps are a feasible QA tool to evaluate the accuracy of deep learning-based sCTs for adaptive proton therapy. The generated uncertainty showed a strong correlation with HU, range, and dose errors, indicating its potential as a built-in, patient-specific QA check for sCTs without needing a reference CT.
69: Kim_2024	sCT Comparison (Cervix)	This study shows that a deep learning-based sCT generation method for cervical cancer is clinically feasible and superior to a commercial segmentation-based method (MRCAT). The DL-based sCT demonstrated better image similarity (lower MAE) and higher dosimetric accuracy (higher gamma passing rate), supporting its use in an MRI-only workflow.
70: Lerner_2022	Clinical Feasibility of MRI-Only (Brain)	This paper reports on a prospective clinical feasibility study that successfully implemented an MRI-only workflow for brain radiotherapy using a commercial deep learning sCT software. 20 of 21 patients were treated successfully, with dosimetric and patient positioning endpoints showing high agreement with the CT-based standard, demonstrating the clinical robustness of the workflow.
71: Wang_2022	sCT for Proton Therapy (Pediatric)	This study develops and validates a deep learning model to synthesize relative proton stopping power (sRPSP) images from MRI for pediatric brain tumor proton therapy. It also creates an online QA tool that uses a reference template to flag anatomical regions with potential inaccuracies in the generated sRPSP, facilitating its safe clinical integration.
72: Chourak_2023	Sensitivity Analysis for sCT	This paper performs a sensitivity analysis to link HU errors in sCT to dose errors for prostate radiotherapy. It concludes that sCT quality assessment should not only focus on organs immediately surrounding the target, as errors in other soft tissues along the beam path can significantly impact the dose in the target volume, providing a new perspective for defining sCT quality thresholds.
73: Hoffmans_2020	End-to-End QA Test	This study demonstrates a comprehensive end-to-end test for an online adaptive MRgRT workflow using a deformable anthropomorphic phantom and film dosimetry. The test successfully validated the entire chain from imaging and plan adaptation to dose delivery, showing good agreement between measured and calculated dose (gamma >96.4% for 3%/2mm) and confirming the suitability of the tools for complex workflow QA.

Table S9. (Group 1) Detailed Data Extracted for the Quantitative Meta-Analysis.

Data Points	Author, Year	Detector Type	Detector_Model	Generation Method	MR-Linac System	B-Field (T)	Detector Axis Relative to Beam	Detector Axis Relative to Magnetic Field	Lorentz Force Effect	Field_Size (cm²)	Output Formalism	Output Value	Output Uncertainty	Uncertainty Type	Reference
**1**	Blum_2021	Diamond	PTW 60019 microDiamond	MC simulations	Elekta Precise linear accelerator	1.50	Parallel	Perpendicular	N/A	1.99 x 1.99	(k_B,Q)	0.9926	0.0010	SD (est.)	estimated from Figures 6 using visual scaling method (phyton)
**2**	Blum_2021	Diamond	PTW 60019 microDiamond	MC simulations	Elekta Precise linear accelerator	1.50	Parallel	Perpendicular	N/A	1.12 x 1.12	(k_B,Q)	0.9676	0.0010	SD (est.)	estimated from Figures 6 using visual scaling method (phyton)
**3**	Blum_2021	Diamond	PTW 60019 microDiamond	MC simulations	Elekta Precise linear accelerator	1.50	Parallel	Perpendicular	N/A	0.63 x 0.63	(k_B,Q)	0.9636	0.0010	SD (est.)	Text, p8
**4**	Blum_2021	Diode	PTW 60023 microSilicon	MC simulations	Elekta Precise linear accelerator	1.50	Parallel	Perpendicular	N/A	1.99 x 1.99	(k_B,Q)	0.9926	0.0010	SD (est.)	estimated from Figures 6 using visual scaling method (phyton)
**5**	Blum_2021	Diode	PTW 60023 microSilicon	MC simulations	Elekta Precise linear accelerator	1.50	Parallel	Perpendicular	N/A	1.12 x 1.12	(k_B,Q)	0.9616	0.0010	SD (est.)	estimated from Figures 6 using visual scaling method (phyton)
**6**	Blum_2021	Diode	PTW 60023 microSilicon	MC simulations	Elekta Precise linear accelerator	1.50	Parallel	Perpendicular	N/A	0.63 x 0.63	(k_B,Q)	0.9377	0.0010	SD (est.)	Text, p8
**7**	Blum_2021	Diamond	PTW 60019 microDiamond	MC simulations	Elekta Precise linear accelerator	0.35	Parallel	Perpendicular	N/A	1.99 x 1.99	(k_B,Q)	0.9976	0.0010	SD (est.)	estimated from Figures 6 using visual scaling method (phyton)
**8**	Blum_2021	Diamond	PTW 60019 microDiamond	MC simulations	Elekta Precise linear accelerator	0.35	Parallel	Perpendicular	N/A	1.12 x 1.12	(k_B,Q)	0.9826	0.0010	SD (est.)	estimated from Figures 6 using visual scaling method (phyton)
**9**	Blum_2021	Diamond	PTW 60019 microDiamond	MC simulations	Elekta Precise linear accelerator	0.35	Parallel	Perpendicular	N/A	0.63 x 0.63	(k_B,Q)	0.9701	0.0010	SD (est.)	estimated from Figures 6 using visual scaling method (phyton)
**10**	Blum_2021	Diode	PTW 60023 microSilicon	MC simulations	Elekta Precise linear accelerator	0.35	Parallel	Perpendicular	N/A	1.99 x 1.99	(k_B,Q)	0.9976	0.0010	SD (est.)	estimated from Figures 6 using visual scaling method (phyton)
**11**	Blum_2021	Diode	PTW 60023 microSilicon	MC simulations	Elekta Precise linear accelerator	0.35	Parallel	Perpendicular	N/A	1.12 x 1.12	(k_B,Q)	0.9876	0.0010	SD (est.)	estimated from Figures 6 using visual scaling method (phyton)
**12**	Blum_2021	Diode	PTW 60023 microSilicon	MC simulations	Elekta Precise linear accelerator	0.35	Parallel	Perpendicular	N/A	0.63 x 0.63	(k_B,Q)	0.9196	0.0010	SD (est.)	estimated from Figures 6 using visual scaling method (phyton)

**13**	Cervantes_2021	Diode	PTW 60012	MC simulations	Elekta Unity	1.50	Parallel	Perpendicular	N/A	3 x 3	(k_B,Q)	1.1070	0.0060	SD	Table 1
**14**	Cervantes_2021	Diode	PTW 60012	MC simulations	Elekta Unity	1.50	Perpendicular	Perpendicular	Force to Stem	3 x 3	(k_B,Q)	0.9840	0.0110	SD	Table 1
**15**	Cervantes_2021	Diode	PTW 60012	MC simulations	Elekta Unity	1.50	Perpendicular	Perpendicular	Force to Tip	3 x 3	(k_B,Q)	0.8750	0.0090	SD	Table 1
**16**	Cervantes_2021	Diode	PTW 60012	MC simulations	Elekta Unity	1.50	Perpendicular	parallel	N/A	3 x 3	(k_B,Q)	1.0020	0.0110	SD	Table 1
**17**	Cervantes_2021	Diode	PTW 60012	MC simulations	Elekta Unity	1.50	parallel	Perpendicular	N/A	2 x 2	(k_B,Q)	1.0970	0.0060	SD	Table 1
**18**	Cervantes_2021	Diode	PTW 60012	MC simulations	Elekta Unity	1.50	Perpendicular	Perpendicular	Force to Stem	2 x 2	(k_B,Q)	0.9840	0.0080	SD	Table 1
**19**	Cervantes_2021	Diode	PTW 60012	MC simulations	Elekta Unity	1.50	Perpendicular	Perpendicular	Force to Tip	2 x 2	(k_B,Q)	0.8680	0.0080	SD	Table 1
**20**	Cervantes_2021	Diode	PTW 60012	MC simulations	Elekta Unity	1.50	Perpendicular	parallel	N/A	2 x 2	(k_B,Q)	0.9940	0.0090	SD	Table 1
**21**	Cervantes_2021	Diode	PTW 60012	MC simulations	Elekta Unity	1.50	parallel	Perpendicular	N/A	1 x 1	(k_B,Q)	1.0760	0.0040	SD	Table 1
**22**	Cervantes_2021	Diode	PTW 60012	MC simulations	Elekta Unity	1.50	Perpendicular	Perpendicular	Force to Stem	1 x 1	(k_B,Q)	1.0020	0.0080	SD	Table 1
**23**	Cervantes_2021	Diode	PTW 60012	MC simulations	Elekta Unity	1.50	Perpendicular	Perpendicular	Force to Tip	1 x 1	(k_B,Q)	0.8570	0.0070	SD	Table 1
**24**	Cervantes_2021	Diode	PTW 60012	MC simulations	Elekta Unity	1.50	Perpendicular	parallel	N/A	1 x 1	(k_B,Q)	0.9840	0.0080	SD	Table 1
**25**	Cervantes_2021	Diode	PTW 60012	MC simulations	Elekta Unity	1.50	parallel	Perpendicular	N/A	0.75 x 0.75	(k_B,Q)	1.0560	0.0030	SD	Table 1
**26**	Cervantes_2021	Diode	PTW 60012	MC simulations	Elekta Unity	1.50	Perpendicular	Perpendicular	Force to Stem	0.75 x 0.75	(k_B,Q)	1.0110	0.0050	SD	Table 1
**27**	Cervantes_20	Diode	PTW 60012	MC simulation	Elekta Unity	1.50	Perpendicular	Perpendicular	Force to Tip	0.75 x	(k_B,Q)	0.8530	0.0050	SD	Table 1

	21			s						0.75					
**28**	Cervantes_2021	Diode	PTW 60012	MC simulations	Elekta Unity	1.50	Perpendicular	parallel	N/A	0.75 x 0.75	(k_B,Q)	0.9760	0.0050	SD	Table 1
**29**	Cervantes_2021	Diode	PTW 60012	MC simulations	Elekta Unity	1.50	parallel	Perpendicular	N/A	0.5 x 0.5	(k_B,Q)	1.0290	0.0030	SD	Table 1
**30**	Cervantes_2021	Diode	PTW 60012	MC simulations	Elekta Unity	1.50	Perpendicular	Perpendicular	Force to Stem	0.5 x 0.5	(k_B,Q)	0.9940	0.0050	SD	Table 1
**31**	Cervantes_2021	Diode	PTW 60012	MC simulations	Elekta Unity	1.50	Perpendicular	Perpendicular	Force to Tip	0.5 x 0.5	(k_B,Q)	0.8550	0.0050	SD	Table 1
**32**	Cervantes_2021	Diode	PTW 60012	MC simulations	Elekta Unity	1.50	Perpendicular	parallel	N/A	0.5 x 0.5	(k_B,Q)	0.9590	0.0050	SD	Table 1
**33**	Cervantes_2021	Diode	PTW 60012	MC simulations	Elekta Unity	1.50	parallel	Perpendicular	N/A	0.25 x 0.25	(k_B,Q)	1.0060	0.0030	SD	Table 1
**34**	Cervantes_2021	Diode	PTW 60012	MC simulations	Elekta Unity	1.50	Perpendicular	Perpendicular	Force to Stem	0.25 x 0.25	(k_B,Q)	0.9840	0.0050	SD	Table 1
**35**	Cervantes_2021	Diode	PTW 60012	MC simulations	Elekta Unity	1.50	Perpendicular	Perpendicular	Force to Tip	0.25 x 0.25	(k_B,Q)	0.8750	0.0040	SD	Table 1
**36**	Cervantes_2021	Diode	PTW 60012	MC simulations	Elekta Unity	1.50	Perpendicular	parallel	N/A	0.25 x 0.25	(k_B,Q)	0.9540	0.0050	SD	Table 1
**37**	Cervantes_2021	Diamond	PTW 60019 microDiamond	MC simulations	Elekta Unity	1.50	parallel	Perpendicular	N/A	3 x 3	(k_B,Q)	1.1810	0.0050	SD	Table 2
**38**	Cervantes_2021	Diamond	PTW 60019 microDiamond	MC simulations	Elekta Unity	1.50	Perpendicular	Perpendicular	Force to Stem	3 x 3	(k_B,Q)	1.2310	0.0080	SD	Table 2
**39**	Cervantes_2021	Diamond	PTW 60019 microDiamond	MC simulations	Elekta Unity	1.50	Perpendicular	Perpendicular	Force to Tip	3 x 3	(k_B,Q)	0.8450	0.0080	SD	Table 2
**40**	Cervantes_2021	Diamond	PTW 60019 microDiamond	MC simulations	Elekta Unity	1.50	Perpendicular	parallel	N/A	3 x 3	(k_B,Q)	0.9940	0.0080	SD	Table 2
**41**	Cervantes_2021	Diamond	PTW 60019 microDiamond	MC simulations	Elekta Unity	1.50	parallel	Perpendicular	N/A	2 x 2	(k_B,Q)	1.1650	0.0040	SD	Table 2

**42**	Cervantes_2021	Diamond	PTW 60019 microDiamond	MC simulations	Elekta Unity	1.50	Perpendicular	Perpendicular	Force to Stem	2 x 2	(k_B,Q)	1.2150	0.0070	SD	Table 2
**43**	Cervantes_2021	Diamond	PTW 60019 microDiamond	MC simulations	Elekta Unity	1.50	Perpendicular	Perpendicular	Force to Tip	2 x 2	(k_B,Q)	0.8360	0.0060	SD	Table 2
**44**	Cervantes_2021	Diamond	PTW 60019 microDiamond	MC simulations	Elekta Unity	1.50	Perpendicular	parallel	N/A	2 x 2	(k_B,Q)	0.9890	0.0070	SD	Table 2
**45**	Cervantes_2021	Diamond	PTW 60019 microDiamond	MC simulations	Elekta Unity	1.50	parallel	Perpendicular	N/A	1 x 1	(k_B,Q)	1.0900	0.0020	SD	Table 2
**46**	Cervantes_2021	Diamond	PTW 60019 microDiamond	MC simulations	Elekta Unity	1.50	Perpendicular	Perpendicular	Force to Stem	1 x 1	(k_B,Q)	1.1380	0.0060	SD	Table 2
**47**	Cervantes_2021	Diamond	PTW 60019 microDiamond	MC simulations	Elekta Unity	1.50	Perpendicular	Perpendicular	Force to Tip	1 x1	(k_B,Q)	0.8280	0.0050	SD	Table 2
**48**	Cervantes_2021	Diamond	PTW 60019 microDiamond	MC simulations	Elekta Unity	1.50	Perpendicular	parallel	N/A	1x 1	(k_B,Q)	0.9620	0.0050	SD	Table 2
**49**	Cervantes_2021	Diamond	PTW 60019 microDiamond	MC simulations	Elekta Unity	1.50	parallel	Perpendicular	N/A	0.75 x 0.75	(k_B,Q)	1.0500	0.0030	SD	Table 2
**50**	Cervantes_2021	Diamond	PTW 60019 microDiamond	MC simulations	Elekta Unity	1.50	Perpendicular	Perpendicular	Force to Stem	0.75 x 0.75	(k_B,Q)	1.0990	0.0030	SD	Table 2
**51**	Cervantes_2021	Diamond	PTW 60019 microDiamond	MC simulations	Elekta Unity	1.50	Perpendicular	Perpendicular	Force to Tip	0.75 x 0.75	(k_B,Q)	0.8250	0.0030	SD	Table 2
**52**	Cervantes_2021	Diamond	PTW 60019 microDiamond	MC simulations	Elekta Unity	1.50	Perpendicular	parallel	N/A	0.75 x 0.75	(k_B,Q)	0.9500	0.0030	SD	Table 2
**53**	Cervantes_2021	Diamond	PTW 60019 microDiamond	MC simulations	Elekta Unity	1.50	parallel	Perpendicular	N/A	0.5 x 0.5	(k_B,Q)	1.0250	0.0020	SD	Table 2
**54**	Cervantes_2021	Diamond	PTW 60019 microDiamond	MC simulations	Elekta Unity	1.50	Perpendicular	Perpendicular	Force to Stem	0.5 x 0.5	(k_B,Q)	1.0430	0.0030	SD	Table 2
**55**	Cervantes_2021	Diamond	PTW 60019 microDiamond	MC simulations	Elekta Unity	1.50	Perpendicular	Perpendicular	Force to Tip	0.5 x 0.5	(k_B,Q)	0.8350	0.0030	SD	Table 2
**56**	Cervantes_20	Diamond	PTW 60019 microDiam	MC simulation	Elekta Unity	1.50	Perpendicular	parallel	N/A	0.5 x 0.5	(k_B,Q)	0.9390	0.0030	SD	Table 2

	21		ond	s											
**57**	Cervantes_2021	Diamond	PTW 60019 microDiamond	MC simulations	Elekta Unity	1.50	parallel	Perpendicular	N/A	0.25 x 0.25	(k_B,Q)	1.0040	0.0030	SD	Table 2
**58**	Cervantes_2021	Diamond	PTW 60019 microDiamond	MC simulations	Elekta Unity	1.50	Perpendicular	Perpendicular	Force to Stem	0.25 x 0.25	(k_B,Q)	1.0130	0.0030	SD	Table 2
**59**	Cervantes_2021	Diamond	PTW 60019 microDiamond	MC simulations	Elekta Unity	1.50	Perpendicular	Perpendicular	Force to Tip	0.25 x 0.25	(k_B,Q)	0.8600	0.0030	SD	Table 2
**60**	Cervantes_2021	Diamond	PTW 60019 microDiamond	MC simulations	Elekta Unity	1.50	Perpendicular	parallel	N/A	0.25 x 0.25	(k_B,Q)	0.9410	0.0030	SD	Table 2
**61**	Cervantes_2021	Ion Chamber	PTW 31010 Semiflex	MC simulations	Elekta Unity	1.50	parallel	Perpendicular	N/A	3 x 3	(k_B,Q)	0.9930	0.0050	SD	Table 3
**62**	Cervantes_2021	Ion Chamber	PTW 31010 Semiflex	MC simulations	Elekta Unity	1.50	Perpendicular	Perpendicular	Force to Stem	3 x 3	(k_B,Q)	1.0120	0.0050	SD	Table 3
**63**	Cervantes_2021	Ion Chamber	PTW 31010 Semiflex	MC simulations	Elekta Unity	1.50	Perpendicular	Perpendicular	Force to Tip	3 x 3	(k_B,Q)	0.9920	0.0050	SD	Table 3
**64**	Cervantes_2021	Ion Chamber	PTW 31010 Semiflex	MC simulations	Elekta Unity	1.50	Perpendicular	parallel	N/A	3 x 3	(k_B,Q)	0.9930	0.0050	SD	Table 3
**65**	Cervantes_2021	Ion Chamber	PTW 31010 Semiflex	MC simulations	Elekta Unity	1.50	parallel	Perpendicular	N/A	2 x 2	(k_B,Q)	0.9940	0.0050	SD	Table 3
**66**	Cervantes_2021	Ion Chamber	PTW 31010 Semiflex	MC simulations	Elekta Unity	1.50	Perpendicular	Perpendicular	Force to Stem	2 x 2	(k_B,Q)	1.0110	0.0050	SD	Table 3
**67**	Cervantes_2021	Ion Chamber	PTW 31010 Semiflex	MC simulations	Elekta Unity	1.50	Perpendicular	Perpendicular	Force to Tip	2 x 2	(k_B,Q)	0.9950	0.0050	SD	Table 3
**68**	Cervantes_2021	Ion Chamber	PTW 31010 Semiflex	MC simulations	Elekta Unity	1.50	Perpendicular	parallel	N/A	2 x 2	(k_B,Q)	0.9980	0.0050	SD	Table 3
**69**	Cervantes_2021	Ion Chamber	PTW 31010 Semiflex	MC simulations	Elekta Unity	1.50	parallel	Perpendicular	N/A	1 x 1	(k_B,Q)	1.0100	0.0050	SD	Table 3
**70**	Cervantes_2021	Ion Chamber	PTW 31010 Semiflex	MC simulations	Elekta Unity	1.50	Perpendicular	Perpendicular	Force to Stem	1 x 1	(k_B,Q)	1.0000	0.0050	SD	Table 3

**71**	Cervantes_2021	Ion Chamber	PTW 31010 Semiflex	MC simulations	Elekta Unity	1.50	Perpendicular	Perpendicular	Force to Tip	1 x1	(k_B,Q)	1.0060	0.0050	SD	Table 3
**72**	Cervantes_2021	Ion Chamber	PTW 31010 Semiflex	MC simulations	Elekta Unity	1.50	Perpendicular	parallel	N/A	1x 1	(k_B,Q)	1.0170	0.0050	SD	Table 3
**73**	Cervantes_2021	Ion Chamber	PTW 31010 Semiflex	MC simulations	Elekta Unity	1.50	parallel	Perpendicular	N/A	0.75 x 0.75	(k_B,Q)	1.0230	0.0040	SD	Table 3
**74**	Cervantes_2021	Ion Chamber	PTW 31010 Semiflex	MC simulations	Elekta Unity	1.50	Perpendicular	Perpendicular	Force to Stem	0.75 x 0.75	(k_B,Q)	0.9900	0.0040	SD	Table 3
**75**	Cervantes_2021	Ion Chamber	PTW 31010 Semiflex	MC simulations	Elekta Unity	1.50	Perpendicular	Perpendicular	Force to Tip	0.75 x 0.75	(k_B,Q)	1.0170	0.0040	SD	Table 3
**76**	Cervantes_2021	Ion Chamber	PTW 31010 Semiflex	MC simulations	Elekta Unity	1.50	Perpendicular	parallel	N/A	0.75 x 0.75	(k_B,Q)	1.0400	0.0040	SD	Table 3
**77**	Cervantes_2021	Ion Chamber	PTW 31010 Semiflex	MC simulations	Elekta Unity	1.50	parallel	Perpendicular	N/A	0.5 x 0.5	(k_B,Q)	1.1520	0.0040	SD	Table 3
**78**	Cervantes_2021	Ion Chamber	PTW 31010 Semiflex	MC simulations	Elekta Unity	1.50	Perpendicular	Perpendicular	Force to Stem	0.5 x 0.5	(k_B,Q)	1.0600	0.0040	SD	Table 3
**79**	Cervantes_2021	Ion Chamber	PTW 31010 Semiflex	MC simulations	Elekta Unity	1.50	Perpendicular	Perpendicular	Force to Tip	0.5 x 0.5	(k_B,Q)	1.0870	0.0040	SD	Table 3
**80**	Cervantes_2021	Ion Chamber	PTW 31010 Semiflex	MC simulations	Elekta Unity	1.50	Perpendicular	parallel	N/A	0.5 x 0.5	(k_B,Q)	1.1690	0.0040	SD	Table 3
**81**	Cervantes_2021	Ion Chamber	PTW 31010 Semiflex	MC simulations	Elekta Unity	1.50	parallel	Perpendicular	N/A	0.25 x 0.25	(k_B,Q)	1.1360	0.0040	SD	Table 3
**82**	Cervantes_2021	Ion Chamber	PTW 31010 Semiflex	MC simulations	Elekta Unity	1.50	Perpendicular	Perpendicular	Force to Stem	0.25 x 0.25	(k_B,Q)	1.0880	0.0040	SD	Table 3
**83**	Cervantes_2021	Ion Chamber	PTW 31010 Semiflex	MC simulations	Elekta Unity	1.50	Perpendicular	Perpendicular	Force to Tip	0.25 x 0.25	(k_B,Q)	1.1160	0.0040	SD	Table 3
**84**	Cervantes_2021	Ion Chamber	PTW 31010 Semiflex	MC simulations	Elekta Unity	1.50	Perpendicular	parallel	N/A	0.25 x 0.25	(k_B,Q)	1.1790	0.0040	SD	Table 3
**85**	Cervantes_20	Ion Chamb	PTW 31021 Semiflex	MC simulation	Elekta Unity	1.50	parallel	Perpendicular	N/A	3 x 3	(k_B,Q)	0.9750	0.0040	SD	Table 4

	21	er	3D	s											
**86**	Cervantes_2021	Ion Chamber	PTW 31021 Semiflex 3D	MC simulations	Elekta Unity	1.50	Perpendicular	Perpendicular	Force to Stem	3 x 3	(k_B,Q)	1.0250	0.0040	SD	Table 4
**87**	Cervantes_2021	Ion Chamber	PTW 31021 Semiflex 3D	MC simulations	Elekta Unity	1.50	Perpendicular	Perpendicular	Force to Tip	3 x 3	(k_B,Q)	0.9760	0.0040	SD	Table 4
**88**	Cervantes_2021	Ion Chamber	PTW 31021 Semiflex 3D	MC simulations	Elekta Unity	1.50	Perpendicular	parallel	N/A	3 x 3	(k_B,Q)	1.0150	0.0050	SD	Table 4
**89**	Cervantes_2021	Ion Chamber	PTW 31021 Semiflex 3D	MC simulations	Elekta Unity	1.50	parallel	Perpendicular	N/A	2 x 2	(k_B,Q)	0.9780	0.0050	SD	Table 4
**90**	Cervantes_2021	Ion Chamber	PTW 31021 Semiflex 3D	MC simulations	Elekta Unity	1.50	Perpendicular	Perpendicular	Force to Stem	2 x 2	(k_B,Q)	1.0220	0.0050	SD	Table 4
**91**	Cervantes_2021	Ion Chamber	PTW 31021 Semiflex 3D	MC simulations	Elekta Unity	1.50	Perpendicular	Perpendicular	Force to Tip	2 x 2	(k_B,Q)	0.9880	0.0040	SD	Table 4
**92**	Cervantes_2021	Ion Chamber	PTW 31021 Semiflex 3D	MC simulations	Elekta Unity	1.50	Perpendicular	parallel	N/A	2 x 2	(k_B,Q)	1.0180	0.0050	SD	Table 4
**93**	Cervantes_2021	Ion Chamber	PTW 31021 Semiflex 3D	MC simulations	Elekta Unity	1.50	parallel	Perpendicular	N/A	1 x 1	(k_B,Q)	0.9860	0.0100	SD	Table 4
**94**	Cervantes_2021	Ion Chamber	PTW 31021 Semiflex 3D	MC simulations	Elekta Unity	1.50	Perpendicular	Perpendicular	Force to Stem	1 x 1	(k_B,Q)	0.9950	0.0050	SD	Table 4
**95**	Cervantes_2021	Ion Chamber	PTW 31021 Semiflex 3D	MC simulations	Elekta Unity	1.50	Perpendicular	Perpendicular	Force to Tip	1 x1	(k_B,Q)	1.0200	0.0050	SD	Table 4
**96**	Cervantes_2021	Ion Chamber	PTW 31021 Semiflex 3D	MC simulations	Elekta Unity	1.50	Perpendicular	parallel	N/A	1x 1	(k_B,Q)	1.0230	0.0050	SD	Table 4
**97**	Cervantes_2021	Ion Chamber	PTW 31021 Semiflex 3D	MC simulations	Elekta Unity	1.50	parallel	Perpendicular	N/A	0.75 x 0.75	(k_B,Q)	0.9800	0.0040	SD	Table 4
**98**	Cervantes_2021	Ion Chamber	PTW 31021 Semiflex 3D	MC simulations	Elekta Unity	1.50	Perpendicular	Perpendicular	Force to Stem	0.75 x 0.75	(k_B,Q)	0.9730	0.0050	SD	Table 4
**99**	Cervantes_2021	Ion Chamber	PTW 31021 Semiflex 3D	MC simulations	Elekta Unity	1.50	Perpendicular	Perpendicular	Force to Tip	0.75 x 0.75	(k_B,Q)	1.0460	0.0040	SD	Table 4

**100**	Cervantes_2021	Ion Chamber	PTW 31021 Semiflex 3D	MC simulations	Elekta Unity	1.50	Perpendicular	parallel	N/A	0.75 x 0.75	(k_B,Q)	1.0370	0.0040	SD	Table 4
**101**	Cervantes_2021	Ion Chamber	PTW 31021 Semiflex 3D	MC simulations	Elekta Unity	1.50	parallel	Perpendicular	N/A	0.5 x 0.5	(k_B,Q)	1.0260	0.0040	SD	Table 4
**102**	Cervantes_2021	Ion Chamber	PTW 31021 Semiflex 3D	MC simulations	Elekta Unity	1.50	Perpendicular	Perpendicular	Force to Stem	0.5 x 0.5	(k_B,Q)	0.9670	0.0040	SD	Table 4
**103**	Cervantes_2021	Ion Chamber	PTW 31021 Semiflex 3D	MC simulations	Elekta Unity	1.50	Perpendicular	Perpendicular	Force to Tip	0.5 x 0.5	(k_B,Q)	1.1300	0.0060	SD	Table 4
**104**	Cervantes_2021	Ion Chamber	PTW 31021 Semiflex 3D	MC simulations	Elekta Unity	1.50	Perpendicular	parallel	N/A	0.5 x 0.5	(k_B,Q)	1.0950	0.0040	SD	Table 4
**105**	Cervantes_2021	Ion Chamber	PTW 31021 Semiflex 3D	MC simulations	Elekta Unity	1.50	parallel	Perpendicular	N/A	0.25 x 0.25	(k_B,Q)	1.2010	0.0040	SD	Table 4
**106**	Cervantes_2021	Ion Chamber	PTW 31021 Semiflex 3D	MC simulations	Elekta Unity	1.50	Perpendicular	Perpendicular	Force to Stem	0.25 x 0.25	(k_B,Q)	1.0660	0.0040	SD	Table 4
**107**	Cervantes_2021	Ion Chamber	PTW 31021 Semiflex 3D	MC simulations	Elekta Unity	1.50	Perpendicular	Perpendicular	Force to Tip	0.25 x 0.25	(k_B,Q)	1.3500	0.0040	SD	Table 4
**108**	Cervantes_2021	Ion Chamber	PTW 31021 Semiflex 3D	MC simulations	Elekta Unity	1.50	Perpendicular	parallel	N/A	0.25 x 0.25	(k_B,Q)	1.2270	0.0040	SD	Table 4
**109**	Cervantes_2021	Ion Chamber	PTW 31022 PinPoint 3D	MC simulations	Elekta Unity	1.50	parallel	Perpendicular	N/A	3 x 3	(k_B,Q)	1.0010	0.0040	SD	Table 5
**110**	Cervantes_2021	Ion Chamber	PTW 31022 PinPoint 3D	MC simulations	Elekta Unity	1.50	Perpendicular	Perpendicular	Force to Stem	3 x 3	(k_B,Q)	1.0290	0.0050	SD	Table 5
**111**	Cervantes_2021	Ion Chamber	PTW 31022 PinPoint 3D	MC simulations	Elekta Unity	1.50	Perpendicular	Perpendicular	Force to Tip	3 x 3	(k_B,Q)	0.9850	0.0050	SD	Table 5
**112**	Cervantes_2021	Ion Chamber	PTW 31022 PinPoint 3D	MC simulations	Elekta Unity	1.50	Perpendicular	parallel	N/A	3 x 3	(k_B,Q)	0.9960	0.0050	SD	Table 5
**113**	Cervantes_2021	Ion Chamber	PTW 31022 PinPoint 3D	MC simulations	Elekta Unity	1.50	parallel	Perpendicular	N/A	2 x 2	(k_B,Q)	1.0050	0.0040	SD	Table 5
**114**	Cervantes_20	Ion Chamb	PTW 31022 PinPoint	MC simulation	Elekta Unity	1.50	Perpendicular	Perpendicular	Force to Stem	2 x 2	(k_B,Q)	1.0260	0.0040	SD	Table 5

	21	er	3D	s											
**115**	Cervantes_2021	Ion Chamber	PTW 31022 PinPoint 3D	MC simulations	Elekta Unity	1.50	Perpendicular	Perpendicular	Force to Tip	2 x 2	(k_B,Q)	0.9970	0.0040	SD	Table 5
**116**	Cervantes_2021	Ion Chamber	PTW 31022 PinPoint 3D	MC simulations	Elekta Unity	1.50	Perpendicular	parallel	N/A	2 x 2	(k_B,Q)	0.9980	0.0040	SD	Table 5
**117**	Cervantes_2021	Ion Chamber	PTW 31022 PinPoint 3D	MC simulations	Elekta Unity	1.50	parallel	Perpendicular	N/A	1 x 1	(k_B,Q)	0.9980	0.0040	SD	Table 5
**118**	Cervantes_2021	Ion Chamber	PTW 31022 PinPoint 3D	MC simulations	Elekta Unity	1.50	Perpendicular	Perpendicular	Force to Stem	1 x 1	(k_B,Q)	1.0120	0.0080	SD	Table 5
**119**	Cervantes_2021	Ion Chamber	PTW 31022 PinPoint 3D	MC simulations	Elekta Unity	1.50	Perpendicular	Perpendicular	Force to Tip	1 x1	(k_B,Q)	1.0120	0.0040	SD	Table 5
**120**	Cervantes_2021	Ion Chamber	PTW 31022 PinPoint 3D	MC simulations	Elekta Unity	1.50	Perpendicular	parallel	N/A	1x 1	(k_B,Q)	0.9980	0.0040	SD	Table 5
**121**	Cervantes_2021	Ion Chamber	PTW 31022 PinPoint 3D	MC simulations	Elekta Unity	1.50	parallel	Perpendicular	N/A	0.75 x 0.75	(k_B,Q)	0.9980	0.0040	SD	Table 5
**122**	Cervantes_2021	Ion Chamber	PTW 31022 PinPoint 3D	MC simulations	Elekta Unity	1.50	Perpendicular	Perpendicular	Force to Stem	0.75 x 0.75	(k_B,Q)	0.9900	0.0040	SD	Table 5
**123**	Cervantes_2021	Ion Chamber	PTW 31022 PinPoint 3D	MC simulations	Elekta Unity	1.50	Perpendicular	Perpendicular	Force to Tip	0.75 x 0.75	(k_B,Q)	1.0230	0.0040	SD	Table 5
**124**	Cervantes_2021	Ion Chamber	PTW 31022 PinPoint 3D	MC simulations	Elekta Unity	1.50	Perpendicular	parallel	N/A	0.75 x 0.75	(k_B,Q)	1.0020	0.0040	SD	Table 5
**125**	Cervantes_2021	Ion Chamber	PTW 31022 PinPoint 3D	MC simulations	Elekta Unity	1.50	parallel	Perpendicular	N/A	0.5 x 0.5	(k_B,Q)	1.0030	0.0040	SD	Table 5
**126**	Cervantes_2021	Ion Chamber	PTW 31022 PinPoint 3D	MC simulations	Elekta Unity	1.50	Perpendicular	Perpendicular	Force to Stem	0.5 x 0.5	(k_B,Q)	0.9720	0.0040	SD	Table 5
**127**	Cervantes_2021	Ion Chamber	PTW 31022 PinPoint 3D	MC simulations	Elekta Unity	1.50	Perpendicular	Perpendicular	Force to Tip	0.5 x 0.5	(k_B,Q)	1.0480	0.0040	SD	Table 5
**128**	Cervantes_2021	Ion Chamber	PTW 31022 PinPoint 3D	MC simulations	Elekta Unity	1.50	Perpendicular	parallel	N/A	0.5 x 0.5	(k_B,Q)	1.0110	0.0040	SD	Table 5

**129**	Cervantes_2021	Ion Chamber	PTW 31022 PinPoint 3D	MC simulations	Elekta Unity	1.50	parallel	Perpendicular	N/A	0.25 x 0.25	(k_B,Q)	1.0800	0.0030	SD	Table 5
**130**	Cervantes_2021	Ion Chamber	PTW 31022 PinPoint 3D	MC simulations	Elekta Unity	1.50	Perpendicular	Perpendicular	Force to Stem	0.25 x 0.25	(k_B,Q)	0.9910	0.0030	SD	Table 5
**131**	Cervantes_2021	Ion Chamber	PTW 31022 PinPoint 3D	MC simulations	Elekta Unity	1.50	Perpendicular	Perpendicular	Force to Tip	0.25 x 0.25	(k_B,Q)	1.1870	0.0030	SD	Table 5
**132**	Cervantes_2021	Ion Chamber	PTW 31022 PinPoint 3D	MC simulations	Elekta Unity	1.50	Perpendicular	parallel	N/A	0.25 x 0.25	(k_B,Q)	1.0910	0.0030	SD	Table 5
**133**	Cervantes_2020	Ion Chamber	PTW 31010 Semiflex	MC simulations	Elekta Synergy	1.50	parallel	Perpendicular	N/A	1.9 x 4.4	(k_B,Q)	1.0015	0.0055	SD	Table V
**134**	Cervantes_2020	Ion Chamber	PTW 31010 Semiflex	MC simulations	Elekta Synergy	1.50	Perpendicular	parallel	N/A	1.9 x 4.4	(k_B,Q)	0.9802	0.0060	SD	Table V
**135**	Cervantes_2020	Ion Chamber	PTW 31016 PinPoint 3D	MC simulations	Elekta Synergy	1.50	parallel	Perpendicular	N/A	1.9 x 4.4	(k_B,Q)	0.9459	0.0064	SD	Table V
**136**	Cervantes_2020	Ion Chamber	PTW 31016 PinPoint 3D	MC simulations	Elekta Synergy	1.50	Perpendicular	parallel	N/A	1.9 x 4.4	(k_B,Q)	0.9466	0.0060	SD	Table V
**137**	Cervantes_2020	Ion Chamber	PTW 31021 Semiflex 3D	MC simulations	Elekta Synergy	1.50	parallel	Perpendicular	N/A	1.9 x 4.4	(k_B,Q)	1.0399	0.0036	SD	Table V
**138**	Cervantes_2020	Ion Chamber	PTW 31021 Semiflex 3D	MC simulations	Elekta Synergy	1.50	Perpendicular	parallel	N/A	1.9 x 4.4	(k_B,Q)	0.9891	0.0041	SD	Table V
**139**	Cervantes_2020	Ion Chamber	PTW 31022 PinPoint 3D	MC simulations	Elekta Synergy	1.50	parallel	Perpendicular	N/A	1.9 x 4.4	(k_B,Q)	1.0243	0.0058	SD	Table V
**140**	Cervantes_2020	Ion Chamber	PTW 31022 PinPoint 3D	MC simulations	Elekta Synergy	1.50	parallel	Perpendicular	N/A	1.9 x 4.4	(k_B,Q)	0.9957	0.0056	SD	Table V
**141**	Cervantes_2020	Ion Chamber	PTW 31010 Semiflex	MC simulations	Elekta Synergy	1.50	parallel	Perpendicular	N/A	1.9 x 4.4	(k_B,Q)	0.9930	0.0055	SD	Table V
**142**	Cervantes_2020	Ion Chamber	PTW 31010 Semiflex	MC simulations	Elekta Synergy	1.50	Perpendicular	parallel	N/A	1.9 x 4.4	(k_B,Q)	0.9981	0.0057	SD	Table V
**143**	Cervantes_20	Ion Chamb	PTW 31016 PinPoint	MC simulation	Elekta Synergy	1.50	parallel	Perpendicular	N/A	1.9 x 4.4	(k_B,Q)	0.9802	0.0065	SD	Table V

	20	er	3D	s											
**144**	Cervantes_2020	Ion Chamber	PTW 31016 PinPoint 3D	MC simulations	Elekta Synergy	1.50	Perpendicular	parallel	N/A	1.9 x 4.4	(k_B,Q)	0.9961	0.0060	SD	Table V
**145**	Cervantes_2020	Ion Chamber	PTW 31021 Semiflex 3D	MC simulations	Elekta Synergy	1.50	parallel	Perpendicular	N/A	1.9 x 4.4	(k_B,Q)	1.0062	0.0036	SD	Table V
**146**	Cervantes_2020	Ion Chamber	PTW 31021 Semiflex 3D	MC simulations	Elekta Synergy	1.50	Perpendicular	parallel	N/A	1.9 x 4.4	(k_B,Q)	1.0003	0.0036	SD	Table V
**147**	Cervantes_2020	Ion Chamber	PTW 31022 PinPoint 3D	MC simulations	Elekta Synergy	1.50	parallel	Perpendicular	N/A	1.9 x 4.4	(k_B,Q)	1.0107	0.0056	SD	Table V
**148**	Cervantes_2020	Ion Chamber	PTW 31022 PinPoint 3D	MC simulations	Elekta Synergy	1.50	parallel	Perpendicular	N/A	1.9 x 4.4	(k_B,Q)	0.9997	0.0056	SD	Table V
**149**	Margaroni_2023	Ion Chamber	PTW 31021 Semiflex 3D	MC simulations	Elekta Unity	1.50	Perpendicular	Perpendicular	Force Parallel to Lorentz Force	3 x 3	(k_B,Q)	1.0195	0.0021	SD (est.)	estimated from Figure 3 using visual scaling method (phyton)
**150**	Margaroni_2023	Ion Chamber	IBA CC04	MC simulations	Elekta Unity	1.50	Perpendicular	Perpendicular	Force Parallel to Lorentz Force	3 x 3	(k_B,Q)	1.0255	0.0032	SD (est.)	estimated from Figure 3 using visual scaling method (phyton)
**151**	Margaroni_2023	Ion Chamber	Exradin A1SL MR	MC simulations	Elekta Unity	1.50	Perpendicular	Perpendicular	Force Parallel to Lorentz Force	3 x 3	(k_B,Q)	1.0055	0.0018	SD (est.)	estimated from Figure 3 using visual scaling method (phyton)
**152**	Margaroni_2023	Ion Chamber	PTW 31022 PinPoint 3D	MC simulations	Elekta Unity	1.50	Perpendicular	Perpendicular	Force Parallel to Lorentz Force	3 x 3	(k_B,Q)	1.0135	0.0026	SD (est.)	estimated from Figure 3 using visual scaling method (phyton)
**153**	Margaroni_2023	Ion Chamber	IBA Razor Chamber	MC simulations	Elekta Unity	1.50	Perpendicular	Perpendicular	Force Parallel to Lorentz Force	3 x 3	(k_B,Q)	1.0005	0.0035	SD (est.)	estimated from Figure 3 using visual scaling method (phyton)
**154**	Margaroni_2023	Ion Chamber	Exradin A26 MR	MC simulations	Elekta Unity	1.50	Perpendicular	Perpendicular	Force Parallel to Lorentz Force	3 x 3	(k_B,Q)	1.0205	0.0028	SD (est.)	estimated from Figure 3 using visual scaling method (phyton)
**155**	Margaroni_2025	Ion Chamber	PTW 31024	MC simulations	Elekta Unity	1.50	Perpendicular	Antiparallel	N/A	3 x 3	(k_B,Q)	0.9940	0.0040	SD (MC)	Tables 4 and 5
**156**	Margaroni_2025	Ion Chamber	PTW 31024	MC simulations	Elekta Unity	1.50	Perpendicular	Antiparallel	N/A	2 x 2	(k_B,Q)	0.9920	0.0040	SD	Tables 4 and 5
**157**	Margaroni_2025	Ion Chamber	PTW 31024	MC simulations	Elekta Unity	1.50	Perpendicular	Antiparallel	N/A	1 x 1	(k_B,Q)	0.9990	0.0040	SD	Tables 4 and 5

**158**	Margaroni_2025	Ion Chamber	PTW 31024	MC simulations	Elekta Unity	1.50	Perpendicular	Parallel	N/A	3 x 3	(k_B,Q)	1.0030	0.0040	SD	Tables 4 and 5
**159**	Margaroni_2025	Ion Chamber	PTW 31024	MC simulations	Elekta Unity	1.50	Perpendicular	Parallel	N/A	2 x 2	(k_B,Q)	0.9990	0.0040	SD	Tables 4 and 5
**160**	Margaroni_2025	Ion Chamber	PTW 31024	MC simulations	Elekta Unity	1.50	Perpendicular	Parallel	N/A	1 x 1	(k_B,Q)	0.9950	0.0040	SD	Tables 4 and 5
**161**	Margaroni_2025	Ion Chamber	PTW 31025	MC simulations	Elekta Unity	1.50	Perpendicular	Antiparallel	N/A	3 x 3	(k_B,Q)	0.9940	0.0040	SD	Tables 4 and 5
**162**	Margaroni_2025	Ion Chamber	PTW 31025	MC simulations	Elekta Unity	1.50	Perpendicular	Antiparallel	N/A	2 x 2	(k_B,Q)	0.9920	0.0040	SD	Tables 4 and 5
**163**	Margaroni_2025	Ion Chamber	PTW 31025	MC simulations	Elekta Unity	1.50	Perpendicular	Antiparallel	N/A	1 x 1	(k_B,Q)	1.0010	0.0040	SD	Tables 4 and 5
**164**	Margaroni_2025	Ion Chamber	PTW 31025	MC simulations	Elekta Unity	1.50	Perpendicular	Parallel	N/A	3 x 3	(k_B,Q)	0.9990	0.0040	SD	Tables 4 and 5
**165**	Margaroni_2025	Ion Chamber	PTW 31025	MC simulations	Elekta Unity	1.50	Perpendicular	Parallel	N/A	2 x 2	(k_B,Q)	0.9900	0.0040	SD	Tables 4 and 5
**166**	Margaroni_2025	Ion Chamber	PTW 31025	MC simulations	Elekta Unity	1.50	Perpendicular	Parallel	N/A	1 x 1	(k_B,Q)	0.9920	0.0040	SD	Tables 4 and 5
**167**	Margaroni_2025	Ion Chamber	IBA Razor Chamber	MC simulations	Elekta Unity	1.50	Perpendicular	Antiparallel	N/A	3 x 3	(k_B,Q)	1.0010	0.0040	SD	Tables 4 and 5
**168**	Margaroni_2025	Ion Chamber	IBA Razor Chamber	MC simulations	Elekta Unity	1.50	Perpendicular	Antiparallel	N/A	2 x 2	(k_B,Q)	1.0030	0.0040	SD	Tables 4 and 5
**169**	Margaroni_2025	Ion Chamber	IBA Razor Chamber	MC simulations	Elekta Unity	1.50	Perpendicular	Antiparallel	N/A	1 x 1	(k_B,Q)	1.0030	0.0040	SD	Tables 4 and 5
**170**	Margaroni_2025	Ion Chamber	IBA Razor Chamber	MC simulations	Elekta Unity	1.50	Perpendicular	Parallel	N/A	3 x 3	(k_B,Q)	1.0030	0.0040	SD	Tables 4 and 5
**171**	Margaroni_2025	Ion Chamber	IBA Razor Chamber	MC simulations	Elekta Unity	1.50	Perpendicular	Parallel	N/A	2 x 2	(k_B,Q)	0.9970	0.0040	SD	Tables 4 and 5
**172**	Margaroni_20	Ion Chamb	IBA Razor Chamber	MC simulation	Elekta Unity	1.50	Perpendicular	Parallel	N/A	1 x 1	(k_B,Q)	0.9940	0.0040	SD	Tables 4 and 5

	25	er		s												
**173**	Margaroni_2025	Ion Chamber	IBA Razor Nano	MC simulations	Elekta Unity	1.50	Perpendicular	Antiparallel	N/A		3 x 3	(k_B,Q)	0.9960	0.0040	SD	Tables 4 and 5
**174**	Margaroni_2025	Ion Chamber	IBA Razor Nano	MC simulations	Elekta Unity	1.50	Perpendicular	Antiparallel	N/A		2 x 2	(k_B,Q)	0.9960	0.0040	SD	Tables 4 and 5
**175**	Margaroni_2025	Ion Chamber	IBA Razor Nano	MC simulations	Elekta Unity	1.50	Perpendicular	Antiparallel	N/A		1 x 1	(k_B,Q)	0.9950	0.0040	SD	Tables 4 and 5
**176**	Margaroni_2025	Ion Chamber	IBA Razor Nano	MC simulations	Elekta Unity	1.50	Perpendicular	Parallel	N/A		3 x 3	(k_B,Q)	0.9910	0.0040	SD	Tables 4 and 5
**177**	Margaroni_2025	Ion Chamber	IBA Razor Nano	MC simulations	Elekta Unity	1.50	Perpendicular	Parallel	N/A		2 x 2	(k_B,Q)	0.9890	0.0040	SD	Tables 4 and 5
**178**	Margaroni_2025	Ion Chamber	IBA Razor Nano	MC simulations	Elekta Unity	1.50	Perpendicular	Parallel	N/A		1 x 1	(k_B,Q)	0.9860	0.0040	SD	Tables 4 and 5
**179**	Margaroni_2025	Ion Chamber	Exradin A1SL MR	MC simulations	Elekta Unity	1.50	Perpendicular	Antiparallel	N/A		3 x 3	(k_B,Q)	1.0040	0.0040	SD	Tables 4 and 5
**180**	Margaroni_2025	Ion Chamber	Exradin A1SL MR	MC simulations	Elekta Unity	1.50	Perpendicular	Antiparallel	N/A		2 x 2	(k_B,Q)	1.0020	0.0040	SD	Tables 4 and 5
**181**	Margaroni_2025	Ion Chamber	Exradin A1SL MR	MC simulations	Elekta Unity	1.50	Perpendicular	Antiparallel	N/A		1 x 1	(k_B,Q)	1.0030	0.0040	SD	Tables 4 and 5
**182**	Margaroni_2025	Ion Chamber	Exradin A1SL MR	MC simulations	Elekta Unity	1.50	Perpendicular	Parallel	N/A		3 x 3	(k_B,Q)	0.9900	0.0040	SD	Tables 4 and 5
**183**	Margaroni_2025	Ion Chamber	Exradin A1SL MR	MC simulations	Elekta Unity	1.50	Perpendicular	Parallel	N/A		2 x 2	(k_B,Q)	0.9930	0.0040	SD	Tables 4 and 5
**184**	Margaroni_2025	Ion Chamber	Exradin A1SL MR	MC simulations	Elekta Unity	1.50	Perpendicular	Parallel	N/A		1 x 1	(k_B,Q)	1.0000	0.0040	SD	Tables 4 and 5
**185**	Margaroni_2025	Ion Chamber	Exradin A26 MR	MC simulations	Elekta Unity	1.50	Perpendicular	Antiparallel	N/A		3 x 3	(k_B,Q)	1.0040	0.0040	SD	Tables 4 and 5
**186**	Margaroni_2025	Ion Chamber	Exradin A26 MR	MC simulations	Elekta Unity	1.50	Perpendicular	Antiparallel	N/A		2 x 2	(k_B,Q)	1.0020	0.0040	SD	Tables 4 and 5

**187**	Margaroni_2025	Ion Chamber	Exradin A26 MR	MC simulations	Elekta Unity	1.50	Perpendicular	Antiparallel	N/A	1 x 1	(k_B,Q)	1.0000	0.0040	SD	Tables 4 and 5
**188**	Margaroni_2025	Ion Chamber	Exradin A26 MR	MC simulations	Elekta Unity	1.50	Perpendicular	Parallel	N/A	3 x 3	(k_B,Q)	0.9990	0.0040	SD	Tables 4 and 5
**189**	Margaroni_2025	Ion Chamber	Exradin A26 MR	MC simulations	Elekta Unity	1.50	Perpendicular	Parallel	N/A	2 x 2	(k_B,Q)	0.9920	0.0040	SD	Tables 4 and 5
**190**	Margaroni_2025	Ion Chamber	Exradin A26 MR	MC simulations	Elekta Unity	1.50	Perpendicular	Parallel	N/A	1 x 1	(k_B,Q)	0.9850	0.0040	SD	Tables 4 and 5
**191**	Margaroni_2025	Diamond	PTW 60019 microDiamond	MC simulations	Elekta Unity	1.50	Perpendicular	Parallel	N/A	3 x 3	(k_B,Q)	1.1060	0.0040	SD	Tables 4 and 5
**192**	Margaroni_2025	Diamond	PTW 60019 microDiamond	MC simulations	Elekta Unity	1.50	Perpendicular	Parallel	N/A	2 x 2	(k_B,Q)	1.0920	0.0040	SD	Tables 4 and 5
**193**	Margaroni_2025	Diamond	PTW 60019 microDiamond	MC simulations	Elekta Unity	1.50	Perpendicular	Parallel	N/A	1 x 1	(k_B,Q)	1.0590	0.0040	SD	Tables 4 and 5
**194**	Margaroni_2025	Diode	PTW 60023 microSilicon	MC simulations	Elekta Unity	1.50	Perpendicular	Parallel	N/A	3 x 3	(k_B,Q)	1.1060	0.0050	SD	Tables 4 and 5
**195**	Margaroni_2025	Diode	PTW 60023 microSilicon	MC simulations	Elekta Unity	1.50	Perpendicular	Parallel	N/A	2 x 2	(k_B,Q)	1.0870	0.0050	SD	Tables 4 and 5
**196**	Margaroni_2025	Diode	PTW 60023 microSilicon	MC simulations	Elekta Unity	1.50	Perpendicular	Parallel	N/A	1 x 1	(k_B,Q)	1.0660	0.0050	SD	Tables 4 and 5
**197**	Margaroni_2025	Diode	IBA Razor Diode	MC simulations	Elekta Unity	1.50	Perpendicular	Parallel	N/A	3 x 3	(k_B,Q)	1.0170	0.0050	SD	Tables 4 and 5
**198**	Margaroni_2025	Diode	IBA Razor Diode	MC simulations	Elekta Unity	1.50	Perpendicular	Parallel	N/A	2 x 2	(k_B,Q)	1.0220	0.0040	SD	Tables 4 and 5
**199**	Margaroni_2025	Diode	IBA Razor Diode	MC simulations	Elekta Unity	1.50	Perpendicular	Parallel	N/A	1 x 1	(k_B,Q)	1.0160	0.0050	SD	Tables 4 and 5
**200**	Margaroni_2025	Plastic Scintillator	Exradin W2	MC simulations	Elekta Unity	1.50	Perpendicular	Parallel	N/A	3 x 3	(k_B,Q)	1.0090	0.0040	SD	Tables 4 and 5
**201**	Margaroni_20	Plastic Scintill	Exradin W2	MC simulation	Elekta Unity	1.50	Perpendicular	Parallel	N/A	2 x 2	(k_B,Q)	1.0020	0.0040	SD	Tables 4 and 5

	25	ator		s											
**202**	Margaroni_2025	Plastic Scintillator	Exradin W2	MC simulations	Elekta Unity	1.50	Perpendicular	Parallel	N/A	1 x 1	(k_B,Q)	1.0150	0.0040	SD	Tables 4 and 5
**203**	Margaroni_2025	TLD	TLD MTS-100M	MC simulations	Elekta Unity	1.50	Both orientations	Both orientations	Both orientations	3 x 3	(k_B,Q)	1.0020	0.0040	SD	Tables 4 and 5
**204**	Margaroni_2025	TLD	TLD MTS-100M	MC simulations	Elekta Unity	1.50	Both orientations	Both orientations	Both orientations	2 x 2	(k_B,Q)	1.0040	0.0040	SD	Tables 4 and 5
**205**	Margaroni_2025	TLD	TLD MTS-100M	MC simulations	Elekta Unity	1.50	Both orientations	Both orientations	Both orientations	1 x 1	(k_B,Q)	0.9960	0.0040	SD	Tables 4 and 5
**206**	Margaroni_2025	Ion Chamber	PTW 31024	MC simulations	Elekta Unity	1.50	Perpendicular	Antiparallel	N/A	3 x 3	(k_clin, msr)	0.9950	0.0040	SD	Table 5
**207**	Margaroni_2025	Ion Chamber	PTW 31024	MC simulations	Elekta Unity	1.50	Perpendicular	Antiparallel	N/A	2 x 2	(k_clin, msr)	0.9990	0.0040	SD	Table 5
**208**	Margaroni_2025	Ion Chamber	PTW 31024	MC simulations	Elekta Unity	1.50	Perpendicular	Antiparallel	N/A	1 x 1	(k_clin, msr)	1.0700	0.0050	SD	Table 5
**209**	Margaroni_2025	Ion Chamber	PTW 31024	MC simulations	Elekta Unity	1.50	Perpendicular	Parallel	N/A	3 x 3	(k_clin, msr)	0.9920	0.0040	SD	Table 5
**210**	Margaroni_2025	Ion Chamber	PTW 31024	MC simulations	Elekta Unity	1.50	Perpendicular	Parallel	N/A	2 x 2	(k_clin, msr)	1.0020	0.0040	SD	Table 5
**211**	Margaroni_2025	Ion Chamber	PTW 31024	MC simulations	Elekta Unity	1.50	Perpendicular	Parallel	N/A	1 x 1	(k_clin, msr)	1.0630	0.0050	SD	Table 5
**212**	Margaroni_2025	Ion Chamber	PTW 31025	MC simulations	Elekta Unity	1.50	Perpendicular	Antiparallel	N/A	3 x 3	(k_clin, msr)	0.9980	0.0040	SD	Table 5
**213**	Margaroni_2025	Ion Chamber	PTW 31025	MC simulations	Elekta Unity	1.50	Perpendicular	Antiparallel	N/A	2 x 2	(k_clin, msr)	0.9980	0.0040	SD	Table 5
**214**	Margaroni_2025	Ion Chamber	PTW 31025	MC simulations	Elekta Unity	1.50	Perpendicular	Antiparallel	N/A	1 x 1	(k_clin, msr)	1.0330	0.0050	SD	Table 5
**215**	Margaroni_2025	Ion Chamber	PTW 31025	MC simulations	Elekta Unity	1.50	Perpendicular	Parallel	N/A	3 x 3	(k_clin, msr)	0.9980	0.0040	SD	Table 5

**216**	Margaroni_2025	Ion Chamber	PTW 31025	MC simulations	Elekta Unity	1.50	Perpendicular	Parallel	N/A	2 x 2	(k_clin, msr)	0.9940	0.0040	SD	Table 5
**217**	Margaroni_2025	Ion Chamber	PTW 31025	MC simulations	Elekta Unity	1.50	Perpendicular	Parallel	N/A	1 x 1	(k_clin, msr)	1.0190	0.0050	SD	Table 5
**218**	Margaroni_2025	Ion Chamber	IBA Razor Chamber	MC simulations	Elekta Unity	1.50	Perpendicular	Antiparallel	N/A	3 x 3	(k_clin, msr)	0.9920	0.0040	SD	Table 5
**219**	Margaroni_2025	Ion Chamber	IBA Razor Chamber	MC simulations	Elekta Unity	1.50	Perpendicular	Antiparallel	N/A	2 x 2	(k_clin, msr)	0.9910	0.0040	SD	Table 5
**220**	Margaroni_2025	Ion Chamber	IBA Razor Chamber	MC simulations	Elekta Unity	1.50	Perpendicular	Antiparallel	N/A	1 x 1	(k_clin, msr)	1.0160	0.0050	SD	Table 5
**221**	Margaroni_2025	Ion Chamber	IBA Razor Chamber	MC simulations	Elekta Unity	1.50	Perpendicular	Parallel	N/A	3 x 3	(k_clin, msr)	0.9970	0.0040	SD	Table 5
**222**	Margaroni_2025	Ion Chamber	IBA Razor Chamber	MC simulations	Elekta Unity	1.50	Perpendicular	Parallel	N/A	2 x 2	(k_clin, msr)	1.0010	0.0040	SD	Table 5
**223**	Margaroni_2025	Ion Chamber	IBA Razor Chamber	MC simulations	Elekta Unity	1.50	Perpendicular	Parallel	N/A	1 x 1	(k_clin, msr)	1.0320	0.0050	SD	Table 5
**224**	Margaroni_2025	Ion Chamber	IBA Razor Nano	MC simulations	Elekta Unity	1.50	Perpendicular	Antiparallel	N/A	3 x 3	(k_clin, msr)	0.9940	0.0040	SD	Table 5
**225**	Margaroni_2025	Ion Chamber	IBA Razor Nano	MC simulations	Elekta Unity	1.50	Perpendicular	Antiparallel	N/A	2 x 2	(k_clin, msr)	0.9910	0.0040	SD	Table 5
**226**	Margaroni_2025	Ion Chamber	IBA Razor Nano	MC simulations	Elekta Unity	1.50	Perpendicular	Antiparallel	N/A	1 x 1	(k_clin, msr)	1.0120	0.0050	SD	Table 5
**227**	Margaroni_2025	Ion Chamber	IBA Razor Nano	MC simulations	Elekta Unity	1.50	Perpendicular	Parallel	N/A	3 x 3	(k_clin, msr)	0.9960	0.0040	SD	Table 5
**228**	Margaroni_2025	Ion Chamber	IBA Razor Nano	MC simulations	Elekta Unity	1.50	Perpendicular	Parallel	N/A	2 x 2	(k_clin, msr)	0.9960	0.0040	SD	Table 5
**229**	Margaroni_2025	Ion Chamber	IBA Razor Nano	MC simulations	Elekta Unity	1.50	Perpendicular	Parallel	N/A	1 x 1	(k_clin, msr)	0.9990	0.0050	SD	Table 5
**230**	Margaroni_20	Ion Chamb	Exradin A1SL MR	MC simulation	Elekta Unity	1.50	Perpendicular	Antiparallel	N/A	3 x 3	(k_clin, msr)	0.9960	0.0040	SD	Table 5

	25	er		s											
**231**	Margaroni_2025	Ion Chamber	Exradin A1SL MR	MC simulations	Elekta Unity	1.50	Perpendicular	Antiparallel	N/A	2 x 2	(k_clin, msr)	0.9950	0.0040	SD	Table 5
**232**	Margaroni_2025	Ion Chamber	Exradin A1SL MR	MC simulations	Elekta Unity	1.50	Perpendicular	Antiparallel	N/A	1 x 1	(k_clin, msr)	1.0510	0.0050	SD	Table 5
**233**	Margaroni_2025	Ion Chamber	Exradin A1SL MR	MC simulations	Elekta Unity	1.50	Perpendicular	Parallel	N/A	3 x 3	(k_clin, msr)	0.9950	0.0040	SD	Table 5
**234**	Margaroni_2025	Ion Chamber	Exradin A1SL MR	MC simulations	Elekta Unity	1.50	Perpendicular	Parallel	N/A	2 x 2	(k_clin, msr)	1.0000	0.0040	SD	Table 5
**235**	Margaroni_2025	Ion Chamber	Exradin A1SL MR	MC simulations	Elekta Unity	1.50	Perpendicular	Parallel	N/A	1 x 1	(k_clin, msr)	1.0370	0.0050	SD	Table 5
**236**	Margaroni_2025	Ion Chamber	Exradin A26 MR	MC simulations	Elekta Unity	1.50	Perpendicular	Antiparallel	N/A	3 x 3	(k_clin, msr)	0.9960	0.0040	SD	Table 5
**237**	Margaroni_2025	Ion Chamber	Exradin A26 MR	MC simulations	Elekta Unity	1.50	Perpendicular	Antiparallel	N/A	2 x 2	(k_clin, msr)	0.9930	0.0040	SD	Table 5
**238**	Margaroni_2025	Ion Chamber	Exradin A26 MR	MC simulations	Elekta Unity	1.50	Perpendicular	Antiparallel	N/A	1 x 1	(k_clin, msr)	1.0260	0.0050	SD	Table 5
**239**	Margaroni_2025	Ion Chamber	Exradin A26 MR	MC simulations	Elekta Unity	1.50	Perpendicular	Parallel	N/A	3 x 3	(k_clin, msr)	1.0000	0.0040	SD	Table 5
**240**	Margaroni_2025	Ion Chamber	Exradin A26 MR	MC simulations	Elekta Unity	1.50	Perpendicular	Parallel	N/A	2 x 2	(k_clin, msr)	0.9940	0.0040	SD	Table 5
**241**	Margaroni_2025	Ion Chamber	Exradin A26 MR	MC simulations	Elekta Unity	1.50	Perpendicular	Parallel	N/A	1 x 1	(k_clin, msr)	1.0220	0.0050	SD	Table 5
**242**	Margaroni_2025	Diamond	PTW 60019 microDiamond	MC simulations	Elekta Unity	1.50	Perpendicular	Parallel	N/A	3 x 3	(k_clin, msr)	0.9940	0.0040	SD	Table 5
**243**	Margaroni_2025	Diamond	PTW 60019 microDiamond	MC simulations	Elekta Unity	1.50	Perpendicular	Parallel	N/A	2 x 2	(k_clin, msr)	0.9890	0.0040	SD	Table 5
**244**	Margaroni_2025	Diamond	PTW 60019 microDiamond	MC simulations	Elekta Unity	1.50	Perpendicular	Parallel	N/A	1 x 1	(k_clin, msr)	0.9560	0.0040	SD	Table 5

**245**	Margaroni_2025	Diode	PTW 60023 microSilicon	MC simulations	Elekta Unity	1.50	Perpendicular	Parallel	N/A	3 x 3	(k_clin, msr)	1.0100	0.0050	SD	Table 5
**246**	Margaroni_2025	Diode	PTW 60023 microSilicon	MC simulations	Elekta Unity	1.50	Perpendicular	Parallel	N/A	2 x 2	(k_clin, msr)	0.9930	0.0050	SD	Table 5
**247**	Margaroni_2025	Diode	PTW 60023 microSilicon	MC simulations	Elekta Unity	1.50	Perpendicular	Parallel	N/A	1 x 1	(k_clin, msr)	0.9590	0.0050	SD	Table 5
**248**	Margaroni_2025	Diode	IBA Razor Diode	MC simulations	Elekta Unity	1.50	Perpendicular	Parallel	N/A	3 x 3	(k_clin, msr)	1.0010	0.0050	SD	Table 5
**249**	Margaroni_2025	Diode	IBA Razor Diode	MC simulations	Elekta Unity	1.50	Perpendicular	Parallel	N/A	2 x 2	(k_clin, msr)	1.0040	0.0050	SD	Table 5
**250**	Margaroni_2025	Diode	IBA Razor Diode	MC simulations	Elekta Unity	1.50	Perpendicular	Parallel	N/A	1 x 1	(k_clin, msr)	1.0070	0.0050	SD	Table 5
**251**	Margaroni_2025	Plastic Scintillator	Exradin W2	MC simulations	Elekta Unity	1.50	Perpendicular	Parallel	N/A	3 x 3	(k_clin, msr)	0.9940	0.0040	SD	Table 5
**252**	Margaroni_2025	Plastic Scintillator	Exradin W2	MC simulations	Elekta Unity	1.50	Perpendicular	Parallel	N/A	2 x 2	(k_clin, msr)	0.9890	0.0050	SD	Table 5
**253**	Margaroni_2025	Plastic Scintillator	Exradin W2	MC simulations	Elekta Unity	1.50	Perpendicular	Parallel	N/A	1 x 1	(k_clin, msr)	1.0040	0.0050	SD	Table 5
**254**	Margaroni_2025	TLD	TLD MTS-100M	MC simulations	Elekta Unity	1.50	Both orientations	Both orientations	Both orientations	3 x 3	(k_clin, msr)	1.0070	0.0040	SD	Table 5
**255**	Margaroni_2025	TLD	TLD MTS-100M	MC simulations	Elekta Unity	1.50	Both orientations	Both orientations	Both orientations	2 x 2	(k_clin, msr)	0.9920	0.0040	SD	Table 5
**256**	Margaroni_2025	TLD	TLD MTS-100M	MC simulations	Elekta Unity	1.50	Both orientations	Both orientations	Both orientations	1 x 1	(k_clin, msr)	1.0230	0.0050	SD	Table 5
**257**	Delfs, 2021	Ion Chamber	PTW 31021 Semiflex 3D	Experiments	Elekta Precise linear accelerator	0.35	Parallel	Perpendicular	N/A	4 x 4	(k_B,Q)	0.9940	0.0045	SD (Table III)	estimated from Figure 5 using visual scaling method (phyton)
**258**	Delfs, 2021	Ion Chamber	PTW 31021 Semiflex 3D	Experiments	Elekta Precise linear accelerator	1.00	Parallel	Perpendicular	N/A	4 x 4	(k_B,Q)	0.9820	0.0045	SD (Table III)	estimated from Figure 5 using visual scaling method (phyton)
**259**	Delfs, 2021	Ion Chamb	PTW 31021 Semiflex	Experiments	Elekta Precise linear	1.40	Parallel	Perpendicular	N/A	4 x 4	(k_B,Q)	0.9800	0.0044	SD (Table III)	estimated from Figure 5 using visual scaling method (phyton)

		er	3D		accelerator										
**260**	Delfs, 2021	Ion Chamber	PTW 31021 Semiflex 3D	MC simulations (eff Vol)	Elekta Precise linear accelerator	0.35	Parallel	Perpendicular	N/A	4 x 4	(k_B,Q)	0.9970	0.0031	SD (Table III)	estimated from Figure 5 using visual scaling method (phyton)
**261**	Delfs, 2021	Ion Chamber	PTW 31021 Semiflex 3D	MC simulations (eff Vol)	Elekta Precise linear accelerator	1.00	Parallel	Perpendicular	N/A	4 x 4	(k_B,Q)	0.9880	0.0030	SD (Table III)	estimated from Figure 5 using visual scaling method (phyton)
**262**	Delfs, 2021	Ion Chamber	PTW 31021 Semiflex 3D	MC simulations (eff Vol)	Elekta Precise linear accelerator	1.40	Parallel	Perpendicular	N/A	4 x 4	(k_B,Q)	0.9760	0.0030	SD (Table III)	estimated from Figure 5 using visual scaling method (phyton)
**263**	Delfs, 2021	Ion Chamber	PTW 31021 Semiflex 3D	MC simulations (full Vol)	Elekta Precise linear accelerator	0.35	Parallel	Perpendicular	N/A	4 x 4	(k_B,Q)	1.0060	0.0031	SD (Table III)	estimated from Figure 5 using visual scaling method (phyton)
**264**	Delfs, 2021	Ion Chamber	PTW 31021 Semiflex 3D	MC simulations (full Vol)	Elekta Precise linear accelerator	1.00	Parallel	Perpendicular	N/A	4 x 4	(k_B,Q)	1.0330	0.0032	SD (Table III)	estimated from Figure 5 using visual scaling method (phyton)
**265**	Delfs, 2021	Ion Chamber	PTW 31021 Semiflex 3D	MC simulations (full Vol)	Elekta Precise linear accelerator	1.40	Parallel	Perpendicular	N/A	4 x 4	(k_B,Q)	1.0460	0.0032	SD (Table III)	estimated from Figure 5 using visual scaling method (phyton)
**266**	Delfs, 2021	Ion Chamber	PTW 31021 Semiflex 3D	Experiments	Elekta Precise linear accelerator	0.35	Perpendicular	Perpendicular	Force to tip	4 x 4	(k_B,Q)	0.9980	0.0045	SD (Table III)	estimated from Figure 5 using visual scaling method (phyton)
**267**	Delfs, 2021	Ion Chamber	PTW 31021 Semiflex 3D	Experiments	Elekta Precise linear accelerator	1.00	Perpendicular	Perpendicular	Force to tip	4 x 4	(k_B,Q)	1.0150	0.0046	SD (Table III)	estimated from Figure 5 using visual scaling method (phyton)
**268**	Delfs, 2021	Ion Chamber	PTW 31021 Semiflex 3D	Experiments	Elekta Precise linear accelerator	1.40	Perpendicular	Perpendicular	Force to tip	4 x 4	(k_B,Q)	1.0300	0.0047	SD (Table III)	estimated from Figure 5 using visual scaling method (phyton)
**269**	Delfs, 2021	Ion Chamber	PTW 31021 Semiflex 3D	Experiments	Elekta Precise linear accelerator	0.35	Perpendicular	Perpendicular	Force to Stem	4 x 4	(k_B,Q)	1.0060	0.0046	SD (Table III)	estimated from Figure 5 using visual scaling method (phyton)
**270**	Delfs, 2021	Ion Chamber	PTW 31021 Semiflex 3D	Experiments	Elekta Precise linear accelerator	1.00	Perpendicular	Perpendicular	Force to Stem	4 x 4	(k_B,Q)	1.0110	0.0046	SD (Table III)	estimated from Figure 5 using visual scaling method (phyton)
**271**	Delfs, 2021	Ion Chamber	PTW 31021 Semiflex 3D	Experiments	Elekta Precise linear accelerator	1.40	Perpendicular	Perpendicular	Force to Stem	4 x 4	(k_B,Q)	1.0080	0.0046	SD (Table III)	estimated from Figure 5 using visual scaling method (phyton)
**272**	Delfs, 2021	Ion Chamber	PTW 31021 Semiflex 3D	MC simulations (eff Vol)	Elekta Precise linear accelerator	0.35	Perpendicular	Perpendicular	Force to tip	4 x 4	(k_B,Q)	0.9980	0.0031	SD (Table III)	estimated from Figure 5 using visual scaling method (phyton)
**273**	Delfs, 2021	Ion Chamber	PTW 31021 Semiflex 3D	MC simulations (eff Vol)	Elekta Precise linear accelerator	1.00	Perpendicular	Perpendicular	Force to tip	4 x 4	(k_B,Q)	1.0130	0.0031	SD (Table III)	estimated from Figure 5 using visual scaling method (phyton)

**274**	Delfs, 2021	Ion Chamber	PTW 31021 Semiflex 3D	MC simulations (eff Vol)	Elekta Precise linear accelerator	1.40	Perpendicular	Perpendicular	Force to tip	4 x 4	(k_B,Q)	1.0340	0.0032	SD (Table III)	estimated from Figure 5 using visual scaling method (phyton)
**275**	Delfs, 2021	Ion Chamber	PTW 31021 Semiflex 3D	MC simulations (eff Vol)	Elekta Precise linear accelerator	1.50	Perpendicular	Perpendicular	Force to tip	4 x 4	(k_B,Q)	1.0390	0.0032	SD (Table III)	estimated from Figure 5 using visual scaling method (phyton)
**276**	Delfs, 2021	Ion Chamber	PTW 31021 Semiflex 3D	MC simulations (eff Vol)	Elekta Precise linear accelerator	0.35	Perpendicular	Perpendicular	Force to stem	4 x 4	(k_B,Q)	1.0060	0.0031	SD (Table III)	estimated from Figure 5 using visual scaling method (phyton)
**277**	Delfs, 2021	Ion Chamber	PTW 31021 Semiflex 3D	MC simulations (eff Vol)	Elekta Precise linear accelerator	1.00	Perpendicular	Perpendicular	Force to stem	4 x 4	(k_B,Q)	1.0160	0.0031	SD (Table III)	estimated from Figure 5 using visual scaling method (phyton)
**278**	Delfs, 2021	Ion Chamber	PTW 31021 Semiflex 3D	MC simulations (eff Vol)	Elekta Precise linear accelerator	1.40	Perpendicular	Perpendicular	Force to stem	4 x 4	(k_B,Q)	1.0150	0.0031	SD (Table III)	estimated from Figure 5 using visual scaling method (phyton)
**279**	Delfs, 2021	Ion Chamber	PTW 31021 Semiflex 3D	MC simulations (eff Vol)	Elekta Precise linear accelerator	1.50	Perpendicular	Perpendicular	Force to stem	4 x 4	(k_B,Q)	1.0140	0.0031	SD (Table III)	estimated from Figure 5 using visual scaling method (phyton)
**280**	Delfs, 2021	Ion Chamber	PTW 31021 Semiflex 3D	MC simulations (full Vol)	Elekta Precise linear accelerator	0.35	Perpendicular	Perpendicular	Force to tip	4 x 4	(k_B,Q)	1.0220	0.0031	SD (Table III)	estimated from Figure 5 using visual scaling method (phyton)
**281**	Delfs, 2021	Ion Chamber	PTW 31021 Semiflex 3D	MC simulations (full Vol)	Elekta Precise linear accelerator	1.00	Perpendicular	Perpendicular	Force to tip	4 x 4	(k_B,Q)	1.0560	0.0033	SD (Table III)	estimated from Figure 5 using visual scaling method (phyton)
**282**	Delfs, 2021	Ion Chamber	PTW 31021 Semiflex 3D	MC simulations (full Vol)	Elekta Precise linear accelerator	1.40	Perpendicular	Perpendicular	Force to tip	4 x 4	(k_B,Q)	1.0620	0.0033	SD (Table III)	estimated from Figure 5 using visual scaling method (phyton)
**283**	Delfs, 2021	Ion Chamber	PTW 31021 Semiflex 3D	MC simulations (full Vol)	Elekta Precise linear accelerator	1.50	Perpendicular	Perpendicular	Force to tip	4 x 4	(k_B,Q)	1.0640	0.0033	SD (Table III)	estimated from Figure 5 using visual scaling method (phyton)
**284**	Delfs, 2021	Ion Chamber	PTW 31021 Semiflex 3D	MC simulations (full Vol)	Elekta Precise linear accelerator	0.35	Perpendicular	Perpendicular	Force to stem	4 x 4	(k_B,Q)	0.9790	0.0030	SD (Table III)	estimated from Figure 5 using visual scaling method (phyton)
**285**	Delfs, 2021	Ion Chamber	PTW 31021 Semiflex 3D	MC simulations (full Vol)	Elekta Precise linear accelerator	1.00	Perpendicular	Perpendicular	Force to stem	4 x 4	(k_B,Q)	0.9690	0.0030	SD (Table III)	estimated from Figure 5 using visual scaling method (phyton)
**286**	Delfs, 2021	Ion Chamber	PTW 31021 Semiflex 3D	MC simulations (full Vol)	Elekta Precise linear accelerator	1.40	Perpendicular	Perpendicular	Force to stem	4 x 4	(k_B,Q)	0.9790	0.0030	SD (Table III)	estimated from Figure 5 using visual scaling method (phyton)
**287**	Delfs, 2021	Ion Chamber	PTW 31021 Semiflex 3D	MC simulations (full Vol)	Elekta Precise linear accelerator	1.50	Perpendicular	Perpendicular	Force to stem	4 x 4	(k_B,Q)	0.9820	0.0030	SD (Table III)	estimated from Figure 5 using visual scaling method (phyton)
**288**	Delfs, 2021	Ion Chamb	PTW 31022 PinPoint	Experiments	Elekta Precise linear	0.35	Parallel	Perpendicular	N/A	4 x 4	(k_B,Q)	0.9930	0.0045	SD (Table III)	estimated from Figure 5 using visual scaling method (phyton)

		er	3D		accelerator										
**289**	Delfs, 2021	Ion Chamber	PTW 31022 PinPoint 3D	Experiments	Elekta Precise linear accelerator	1.00	Parallel	Perpendicular	N/A	4 x 4	(k_B,Q)	0.9880	0.0045	SD (Table III)	estimated from Figure 5 using visual scaling method (phyton)
**290**	Delfs, 2021	Ion Chamber	PTW 31022 PinPoint 3D	Experiments	Elekta Precise linear accelerator	1.40	Parallel	Perpendicular	N/A	4 x 4	(k_B,Q)	0.9960	0.0045	SD (Table III)	estimated from Figure 5 using visual scaling method (phyton)
**291**	Delfs, 2021	Ion Chamber	PTW 31022 PinPoint 3D	MC simulations (eff Vol)	Elekta Precise linear accelerator	0.35	Parallel	Perpendicular	N/A	4 x 4	(k_B,Q)	1.0000	0.0031	SD (Table III)	estimated from Figure 5 using visual scaling method (phyton)
**292**	Delfs, 2021	Ion Chamber	PTW 31022 PinPoint 3D	MC simulations (eff Vol)	Elekta Precise linear accelerator	1.00	Parallel	Perpendicular	N/A	4 x 4	(k_B,Q)	1.0020	0.0031	SD (Table III)	estimated from Figure 5 using visual scaling method (phyton)
**293**	Delfs, 2021	Ion Chamber	PTW 31022 PinPoint 3D	MC simulations (eff Vol)	Elekta Precise linear accelerator	1.40	Parallel	Perpendicular	N/A	4 x 4	(k_B,Q)	1.0030	0.0031	SD (Table III)	estimated from Figure 5 using visual scaling method (phyton)
**294**	Delfs, 2021	Ion Chamber	PTW 31022 PinPoint 3D	MC simulations (eff Vol)	Elekta Precise linear accelerator	1.50	Parallel	Perpendicular	N/A	4 x 4	(k_B,Q)	1.0020	0.0031	SD (Table III)	estimated from Figure 5 using visual scaling method (phyton)
**295**	Delfs, 2021	Ion Chamber	PTW 31022 PinPoint 3D	MC simulations (full Vol)	Elekta Precise linear accelerator	0.35	Parallel	Perpendicular	N/A	4 x 4	(k_B,Q)	1.0030	0.0031	SD (Table III)	estimated from Figure 5 using visual scaling method (phyton)
**296**	Delfs, 2021	Ion Chamber	PTW 31022 PinPoint 3D	MC simulations (full Vol)	Elekta Precise linear accelerator	1.00	Parallel	Perpendicular	N/A	4 x 4	(k_B,Q)	1.0180	0.0031	SD (Table III)	estimated from Figure 5 using visual scaling method (phyton)
**297**	Delfs, 2021	Ion Chamber	PTW 31022 PinPoint 3D	MC simulations (full Vol)	Elekta Precise linear accelerator	1.40	Parallel	Perpendicular	N/A	4 x 4	(k_B,Q)	1.0320	0.0032	SD (Table III)	estimated from Figure 5 using visual scaling method (phyton)
**298**	Delfs, 2021	Ion Chamber	PTW 31022 PinPoint 3D	MC simulations (full Vol)	Elekta Precise linear accelerator	1.50	Parallel	Perpendicular	N/A	4x 4	(k_B,Q)	1.0340	0.0032	SD (Table III)	estimated from Figure 5 using visual scaling method (phyton)
**299**	Delfs, 2021	Ion Chamber	PTW 31022 PinPoint 3D	Experiments	Elekta Precise linear accelerator	0.35	Perpendicular	Perpendicular	Force to tip	4 x 4	(k_B,Q)	0.9980	0.0045	SD (Table III)	estimated from Figure 5 using visual scaling method (phyton)
**300**	Delfs, 2021	Ion Chamber	PTW 31022 PinPoint 3D	Experiments	Elekta Precise linear accelerator	1.00	Perpendicular	Perpendicular	Force to tip	4 x 4	(k_B,Q)	1.0160	0.0046	SD (Table III)	estimated from Figure 5 using visual scaling method (phyton)
**301**	Delfs, 2021	Ion Chamber	PTW 31022 PinPoint 3D	Experiments	Elekta Precise linear accelerator	1.40	Perpendicular	Perpendicular	Force to tip	4 x 4	(k_B,Q)	1.0270	0.0047	SD (Table III)	estimated from Figure 5 using visual scaling method (phyton)
**302**	Delfs, 2021	Ion Chamber	PTW 31022 PinPoint 3D	Experiments	Elekta Precise linear accelerator	0.35	Perpendicular	Perpendicular	Force to Stem	4 x 4	(k_B,Q)	1.0050	0.0046	SD (Table III)	estimated from Figure 5 using visual scaling method (phyton)

**303**	Delfs, 2021	Ion Chamber	PTW 31022 PinPoint 3D	Experiments	Elekta Precise linear accelerator	1.00	Perpendicular	Perpendicular	Force to Stem	4 x 4	(k_B,Q)	1.0080	0.0046	SD (Table III)	estimated from Figure 5 using visual scaling method (phyton)
**304**	Delfs, 2021	Ion Chamber	PTW 31022 PinPoint 3D	Experiments	Elekta Precise linear accelerator	1.40	Perpendicular	Perpendicular	Force to Stem	4 x 4	(k_B,Q)	1.0200	0.0046	SD (Table III)	estimated from Figure 5 using visual scaling method (phyton)
**305**	Delfs, 2021	Ion Chamber	PTW 31022 PinPoint 3D	MC simulations (eff Vol)	Elekta Precise linear accelerator	0.35	Perpendicular	Perpendicular	Force to tip	4 x 4	(k_B,Q)	1.0060	0.0031	SD (Table III)	estimated from Figure 5 using visual scaling method (phyton)
**306**	Delfs, 2021	Ion Chamber	PTW 31022 PinPoint 3D	MC simulations (eff Vol)	Elekta Precise linear accelerator	1.00	Perpendicular	Perpendicular	Force to tip	4 x 4	(k_B,Q)	1.0180	0.0031	SD (Table III)	estimated from Figure 5 using visual scaling method (phyton)
**307**	Delfs, 2021	Ion Chamber	PTW 31022 PinPoint 3D	MC simulations (eff Vol)	Elekta Precise linear accelerator	1.40	Perpendicular	Perpendicular	Force to tip	4 x 4	(k_B,Q)	1.0310	0.0032	SD (Table III)	estimated from Figure 5 using visual scaling method (phyton)
**308**	Delfs, 2021	Ion Chamber	PTW 31022 PinPoint 3D	MC simulations (eff Vol)	Elekta Precise linear accelerator	1.50	Perpendicular	Perpendicular	Force to tip	4 x 4	(k_B,Q)	1.0350	0.0032	SD (Table III)	estimated from Figure 5 using visual scaling method (phyton)
**309**	Delfs, 2021	Ion Chamber	PTW 31022 PinPoint 3D	MC simulations (eff Vol)	Elekta Precise linear accelerator	0.35	Perpendicular	Perpendicular	Force to Stem	4 x 4	(k_B,Q)	0.9980	0.0031	SD (Table III)	estimated from Figure 5 using visual scaling method (phyton)
**310**	Delfs, 2021	Ion Chamber	PTW 31022 PinPoint 3D	MC simulations (eff Vol)	Elekta Precise linear accelerator	1.00	Perpendicular	Perpendicular	Force to Stem	4 x 4	(k_B,Q)	1.0030	0.0031	SD (Table III)	estimated from Figure 5 using visual scaling method (phyton)
**311**	Delfs, 2021	Ion Chamber	PTW 31022 PinPoint 3D	MC simulations (eff Vol)	Elekta Precise linear accelerator	1.40	Perpendicular	Perpendicular	Force to Stem	4 x 4	(k_B,Q)	1.0110	0.0031	SD (Table III)	estimated from Figure 5 using visual scaling method (phyton)
**312**	Delfs, 2021	Ion Chamber	PTW 31022 PinPoint 3D	MC simulations (eff Vol)	Elekta Precise linear accelerator	1.50	Perpendicular	Perpendicular	Force to Stem	4 x 4	(k_B,Q)	1.0140	0.0031	SD (Table III)	estimated from Figure 5 using visual scaling method (phyton)
**313**	Delfs, 2021	Ion Chamber	PTW 31022 PinPoint 3D	MC simulations (eff Vol)	Elekta Precise linear accelerator	0.35	Perpendicular	Perpendicular	Force to tip	4 x 4	(k_B,Q)	1.0150	0.0031	SD (Table III)	estimated from Figure 5 using visual scaling method (phyton)
**314**	Delfs, 2021	Ion Chamber	PTW 31022 PinPoint 3D	MC simulations (eff Vol)	Elekta Precise linear accelerator	1.00	Perpendicular	Perpendicular	Force to tip	4 x 4	(k_B,Q)	1.0420	0.0032	SD (Table III)	estimated from Figure 5 using visual scaling method (phyton)
**315**	Delfs, 2021	Ion Chamber	PTW 31022 PinPoint 3D	MC simulations (eff Vol)	Elekta Precise linear accelerator	1.40	Perpendicular	Perpendicular	Force to tip	4 x 4	(k_B,Q)	1.0500	0.0032	SD (Table III)	estimated from Figure 5 using visual scaling method (phyton)
**316**	Delfs, 2021	Ion Chamber	PTW 31022 PinPoint 3D	MC simulations (eff Vol)	Elekta Precise linear accelerator	1.50	Perpendicular	Perpendicular	Force to tip	4 x 4	(k_B,Q)	1.0520	0.0032	SD (Table III)	estimated from Figure 5 using visual scaling method (phyton)
**317**	Delfs, 2021	Ion Chamb	PTW 31022 PinPoint	MC simulation	Elekta Precise linear	0.35	Perpendicular	Perpendicular	Force to Stem	4 x 4	(k_B,Q)	0.9880	0.0030	SD (Table III)	estimated from Figure 5 using visual scaling method (phyton)

		er	3D	s (eff Vol)	accelerator										
**318**	Delfs, 2021	Ion Chamber	PTW 31022 PinPoint 3D	MC simulations (eff Vol)	Elekta Precise linear accelerator	1.00	Perpendicular	Perpendicular	Force to Stem	4 x 4	(k_B,Q)	0.9780	0.0030	SD (Table III)	estimated from Figure 5 using visual scaling method (phyton)
**319**	Delfs, 2021	Ion Chamber	PTW 31022 PinPoint 3D	MC simulations (eff Vol)	Elekta Precise linear accelerator	1.40	Perpendicular	Perpendicular	Force to Stem	4 x 4	(k_B,Q)	0.9810	0.0030	SD (Table III)	estimated from Figure 5 using visual scaling method (phyton)
**320**	Delfs, 2021	Ion Chamber	PTW 31022 PinPoint 3D	MC simulations (eff Vol)	Elekta Precise linear accelerator	1.50	Perpendicular	Perpendicular	Force to Stem	4 x 4	(k_B,Q)	0.9820	0.0030	SD (Table III)	estimated from Figure 5 using visual scaling method (phyton)
**321**	Delfs, 2021	Ion Chamber	SNC125c	Experiments	Elekta Precise linear accelerator	0.35	Parallel	Perpendicular	N/A	4 x 4	(k_B,Q)	1.0070	0.0046	SD (Table III)	estimated from Figure 5 using visual scaling method (phyton)
**322**	Delfs, 2021	Ion Chamber	SNC125c	Experiments	Elekta Precise linear accelerator	1.00	Parallel	Perpendicular	N/A	4 x 4	(k_B,Q)	1.0340	0.0047	SD (Table III)	estimated from Figure 5 using visual scaling method (phyton)
**323**	Delfs, 2021	Ion Chamber	SNC125c	Experiments	Elekta Precise linear accelerator	1.40	Parallel	Perpendicular	N/A	4 x 4	(k_B,Q)	1.0420	0.0047	SD (Table III)	estimated from Figure 5 using visual scaling method (phyton)
**324**	Delfs, 2021	Ion Chamber	SNC125c	MC simulations	Elekta Precise linear accelerator	0.35	Parallel	Perpendicular	N/A	4 x 4	(k_B,Q)	1.0090	0.0031	SD (Table III)	estimated from Figure 5 using visual scaling method (phyton)
**325**	Delfs, 2021	Ion Chamber	SNC125c	MC simulations	Elekta Precise linear accelerator	1.00	Parallel	Perpendicular	N/A	4 x 4	(k_B,Q)	1.0320	0.0032	SD (Table III)	estimated from Figure 5 using visual scaling method (phyton)
**326**	Delfs, 2021	Ion Chamber	SNC125c	MC simulations	Elekta Precise linear accelerator	1.40	Parallel	Perpendicular	N/A	4 x 4	(k_B,Q)	1.0280	0.0032	SD (Table III)	estimated from Figure 5 using visual scaling method (phyton)
**327**	Delfs, 2021	Ion Chamber	SNC125c	MC simulations	Elekta Precise linear accelerator	1.50	Parallel	Perpendicular	N/A	4 x 4	(k_B,Q)	1.0260	0.0032	SD (Table III)	estimated from Figure 5 using visual scaling method (phyton)
**328**	Delfs, 2021	Ion Chamber	SNC125c	Experiments	Elekta Precise linear accelerator	0.35	Perpendicular	Perpendicular	Force to tip	4 x 4	(k_B,Q)	0.9940	0.0045	SD (Table III)	estimated from Figure 5 using visual scaling method (phyton)
**329**	Delfs, 2021	Ion Chamber	SNC125c	Experiments	Elekta Precise linear accelerator	1.00	Perpendicular	Perpendicular	Force to tip	4 x 4	(k_B,Q)	0.9930	0.0045	SD (Table III)	estimated from Figure 5 using visual scaling method (phyton)
**330**	Delfs, 2021	Ion Chamber	SNC125c	Experiments	Elekta Precise linear accelerator	1.40	Perpendicular	Perpendicular	Force to tip	4 x 4	(k_B,Q)	0.9920	0.0045	SD (Table III)	estimated from Figure 5 using visual scaling method (phyton)
**331**	Delfs, 2021	Ion Chamber	SNC125c	Experiments	Elekta Precise linear accelerator	0.35	Perpendicular	Perpendicular	Force to Stem	4 x 4	(k_B,Q)	0.9890	0.0045	SD (Table III)	estimated from Figure 5 using visual scaling method (phyton)

**332**	Delfs, 2021	Ion Chamber	SNC125c	Experiments	Elekta Precise linear accelerator	1.00	Perpendicular	Perpendicular	Force to Stem	4 x 4	(k_B,Q)	0.9750	0.0044	SD (Table III)	estimated from Figure 5 using visual scaling method (phyton)
**333**	Delfs, 2021	Ion Chamber	SNC125c	Experiments	Elekta Precise linear accelerator	1.40	Perpendicular	Perpendicular	Force to Stem	4 x 4	(k_B,Q)	0.9800	0.0044	SD (Table III)	estimated from Figure 5 using visual scaling method (phyton)
**334**	Delfs, 2021	Ion Chamber	SNC125c	MC simulations	Elekta Precise linear accelerator	0.35	Perpendicular	Perpendicular	Force to tip	4 x 4	(k_B,Q)	0.9880	0.0030	SD (Table III)	estimated from Figure 5 using visual scaling method (phyton)
**335**	Delfs, 2021	Ion Chamber	SNC125c	MC simulations	Elekta Precise linear accelerator	1.00	Perpendicular	Perpendicular	Force to tip	4 x 4	(k_B,Q)	0.9850	0.0030	SD (Table III)	estimated from Figure 5 using visual scaling method (phyton)
**336**	Delfs, 2021	Ion Chamber	SNC125c	MC simulations	Elekta Precise linear accelerator	1.40	Perpendicular	Perpendicular	Force to tip	4 x 4	(k_B,Q)	0.9890	0.0030	SD (Table III)	estimated from Figure 5 using visual scaling method (phyton)
**337**	Delfs, 2021	Ion Chamber	SNC125c	MC simulations	Elekta Precise linear accelerator	1.50	Perpendicular	Perpendicular	Force to tip	4 x 4	(k_B,Q)	0.9930	0.0031	SD (Table III)	estimated from Figure 5 using visual scaling method (phyton)
**338**	Delfs, 2021	Ion Chamber	SNC125c	MC simulations	Elekta Precise linear accelerator	0.35	Perpendicular	Perpendicular	Force to Stem	4 x 4	(k_B,Q)	0.9890	0.0030	SD (Table III)	estimated from Figure 5 using visual scaling method (phyton)
**339**	Delfs, 2021	Ion Chamber	SNC125c	MC simulations	Elekta Precise linear accelerator	1.00	Perpendicular	Perpendicular	Force to Stem	4 x 4	(k_B,Q)	0.9740	0.0030	SD (Table III)	estimated from Figure 5 using visual scaling method (phyton)
**340**	Delfs, 2021	Ion Chamber	SNC125c	MC simulations	Elekta Precise linear accelerator	1.40	Perpendicular	Perpendicular	Force to Stem	4 x 4	(k_B,Q)	0.9780	0.0030	SD (Table III)	estimated from Figure 5 using visual scaling method (phyton)
**341**	Delfs, 2021	Ion Chamber	SNC125c	MC simulations	Elekta Precise linear accelerator	1.50	Perpendicular	Perpendicular	Force to Stem	4 x 4	(k_B,Q)	0.9820	0.0030	SD (Table III)	estimated from Figure 5 using visual scaling method (phyton)
**342**	Tekin, 2020	Diamond	PTW 60019 microDiamond	Experiments	Elekta Precise linear accelerator	0.35	Parallel	Perpendicular	N/A	4 x 4	(k_B,Q)	1.0080	0.0030	SD (Type A)	estimated from Figures 5 and 9 using visual scaling method (phyton)
**343**	Tekin, 2020	Diamond	PTW 60019 microDiamond	Experiments	Elekta Precise linear accelerator	1.00	Parallel	Perpendicular	N/A	4 x 4	(k_B,Q)	1.0520	0.0030	SD (Type A)	estimated from Figures 5 and 9 using visual scaling method (phyton)
**344**	Tekin, 2020	Diamond	PTW 60019 microDiamond	Experiments	Elekta Precise linear accelerator	1.40	Parallel	Perpendicular	N/A	4 x 4	(k_B,Q)	1.0950	0.0030	SD (Type A)	estimated from Figures 5 and 9 using visual scaling method (phyton)
**345**	Tekin, 2020	Diamond	PTW 60019 microDiamond	MC simulations	Elekta Precise linear accelerator	0.35	Parallel	Perpendicular	N/A	4 x 4	(k_B,Q)	1.0060	0.0020	SD (Type A)	estimated from Figures 5 and 9 using visual scaling method (phyton)
**346**	Tekin, 2020	Diamond	PTW 60019 microDiam	MC simulation	Elekta Precise linear	1.00	Parallel	Perpendicular	N/A	4 x 4	(k_B,Q)	1.0500	0.0020	SD (Type A)	estimated from Figures 5 and 9 using visual scaling method

			ond	s	accelerator										(phyton)
**347**	Tekin, 2020	Diamond	PTW 60019 microDiamond	MC simulations	Elekta Precise linear accelerator	1.50	Parallel	Perpendicular	N/A	4 x 4	(k_B,Q)	1.0980	0.0020	SD (Type A)	estimated from Figures 5 and 9 using visual scaling method (phyton)
**348**	Tekin, 2020	Diode	PTW 60023 microSilicon	Experiments	Elekta Precise linear accelerator	0.35	Parallel	Perpendicular	N/A	4 x 4	(k_B,Q)	1.0100	0.0030	SD (Type A)	estimated from Figures 5 and 9 using visual scaling method (phyton)
**349**	Tekin, 2020	Diode	PTW 60023 microSilicon	Experiments	Elekta Precise linear accelerator	1.00	Parallel	Perpendicular	N/A	4 x 4	(k_B,Q)	1.0620	0.0030	SD (Type A)	estimated from Figures 5 and 9 using visual scaling method (phyton)
**350**	Tekin, 2020	Diode	PTW 60023 microSilicon	Experiments	Elekta Precise linear accelerator	1.40	Parallel	Perpendicular	N/A	4 x 4	(k_B,Q)	1.1150	0.0030	SD (Type A)	estimated from Figures 5 and 9 using visual scaling method (phyton)
**351**	Tekin, 2020	Diode	PTW 60023 microSilicon	MC simulations	Elekta Precise linear accelerator	0.35	Parallel	Perpendicular	N/A	4 x 4	(k_B,Q)	1.0080	0.0020	SD (Type A)	estimated from Figures 5 and 9 using visual scaling method (phyton)
**352**	Tekin, 2020	Diode	PTW 60023 microSilicon	MC simulations	Elekta Precise linear accelerator	1.00	Parallel	Perpendicular	N/A	4 x 4	(k_B,Q)	1.0600	0.0020	SD (Type A)	estimated from Figures 5 and 9 using visual scaling method (phyton)
**353**	Tekin, 2020	Diode	PTW 60023 microSilicon	MC simulations	Elekta Precise linear accelerator	1.50	Parallel	Perpendicular	N/A	4 x 4	(k_B,Q)	1.1120	0.0020	SD (Type A)	estimated from Figures 5 and 9 using visual scaling method (phyton)
**354**	Tekin, 2020	Diode	IBA Razor diode	Experiments	Elekta Precise linear accelerator	0.35	Parallel	Perpendicular	N/A	4 x 4	(k_B,Q)	1.0050	0.0030	SD (Type A)	estimated from Figures 5 and 9 using visual scaling method (phyton)
**355**	Tekin, 2020	Diode	IBA Razor diode	Experiments	Elekta Precise linear accelerator	1.00	Parallel	Perpendicular	N/A	4 x 4	(k_B,Q)	1.0280	0.0030	SD (Type A)	estimated from Figures 5 and 9 using visual scaling method (phyton)
**356**	Tekin, 2020	Diode	IBA Razor diode	Experiments	Elekta Precise linear accelerator	1.40	Parallel	Perpendicular	N/A	4 x 4	(k_B,Q)	1.0520	0.0030	SD (Type A)	estimated from Figures 5 and 9 using visual scaling method (phyton)
**357**	Tekin, 2020	Diode	IBA Razor diode	MC simulations	Elekta Precise linear accelerator	0.35	Parallel	Perpendicular	N/A	4 x 4	(k_B,Q)	1.0040	0.0020	SD (Type A)	estimated from Figures 5 and 9 using visual scaling method (phyton)
**358**	Tekin, 2020	Diode	IBA Razor diode	MC simulations	Elekta Precise linear accelerator	1.00	Parallel	Perpendicular	N/A	4 x 4	(k_B,Q)	1.0260	0.0020	SD (Type A)	estimated from Figures 5 and 9 using visual scaling method (phyton)
**359**	Tekin, 2020	Diode	IBA Razor diode	MC simulations	Elekta Precise linear accelerator	1.50	Parallel	Perpendicular	N/A	4 x 4	(k_B,Q)	1.0640	0.0020	SD (Type A)	estimated from Figures 5 and 9 using visual scaling method (phyton)
**360**	Tekin, 2020	Diamond	PTW 60019 microDiamond	MC simulations	Elekta Precise linear accelerator	0.35	Perpendicular	Perpendicular	Force to tip	4 x 4	(k_B,Q)	1.0680	0.0020	SD (Type A)	estimated from Figures 5 and 9 using visual scaling method (phyton)

**361**	Tekin, 2020	Diamond	PTW 60019 microDiamond	MC simulations	Elekta Precise linear accelerator	1.00	Perpendicular	Perpendicular	Force to tip	4 x 4	(k_B,Q)	1.0820	0.0020	SD (Type A)	estimated from Figures 5 and 9 using visual scaling method (phyton)
**362**	Tekin, 2020	Diamond	PTW 60019 microDiamond	MC simulations	Elekta Precise linear accelerator	1.50	Perpendicular	Perpendicular	Force to tip	4 x 4	(k_B,Q)	1.0740	0.0020	SD (Type A)	estimated from Figures 5 and 9 using visual scaling method (phyton)
**363**	Tekin, 2020	Diamond	PTW 60019 microDiamond	MC simulations	Elekta Precise linear accelerator	0.35	Perpendicular	Perpendicular	Force to stem	4 x 4	(k_B,Q)	0.9570	0.0020	SD (Type A)	estimated from Figures 5 and 9 using visual scaling method (phyton)
**364**	Tekin, 2020	Diamond	PTW 60019 microDiamond	MC simulations	Elekta Precise linear accelerator	1.00	Perpendicular	Perpendicular	Force to stem	4 x 4	(k_B,Q)	0.9210	0.0020	SD (Type A)	estimated from Figures 5 and 9 using visual scaling method (phyton)
**365**	Tekin, 2020	Diamond	PTW 60019 microDiamond	MC simulations	Elekta Precise linear accelerator	1.50	Perpendicular	Perpendicular	Force to stem	4 x 4	(k_B,Q)	0.9010	0.0020	SD (Type A)	estimated from Figures 5 and 9 using visual scaling method (phyton)
**366**	Tekin, 2020	Diode	PTW 60023 microSilicon	MC simulations	Elekta Precise linear accelerator	0.35	Perpendicular	Perpendicular	Force to tip	4 x 4	(k_B,Q)	1.0860	0.0020	SD (Type A)	estimated from Figures 5 and 9 using visual scaling method (phyton)
**367**	Tekin, 2021	Diode	PTW 60023 microSilicon	MC simulations	Elekta Precise linear accelerator	1.00	Perpendicular	Perpendicular	Force to tip	4 x 4	(k_B,Q)	1.1150	0.0020	SD (Type A)	estimated from Figures 5 and 9 using visual scaling method (phyton)
**368**	Tekin, 2022	Diode	PTW 60023 microSilicon	MC simulations	Elekta Precise linear accelerator	1.50	Perpendicular	Perpendicular	Force to tip	4 x 4	(k_B,Q)	1.1200	0.0020	SD (Type A)	estimated from Figures 5 and 9 using visual scaling method (phyton)
**369**	Tekin, 2020	Diode	PTW 60023 microSilicon	MC simulations	Elekta Precise linear accelerator	0.35	Perpendicular	Perpendicular	Force to stem	4 x 4	(k_B,Q)	0.9410	0.0020	SD (Type A)	estimated from Figures 5 and 9 using visual scaling method (phyton)
**370**	Tekin, 2020	Diode	PTW 60023 microSilicon	MC simulations	Elekta Precise linear accelerator	1.00	Perpendicular	Perpendicular	Force to stem	4 x 4	(k_B,Q)	0.8700	0.0020	SD (Type A)	estimated from Figures 5 and 9 using visual scaling method (phyton)
**371**	Tekin, 2020	Diode	PTW 60023 microSilicon	MC simulations	Elekta Precise linear accelerator	1.50	Perpendicular	Perpendicular	Force to stem	4 x 4	(k_B,Q)	0.8520	0.0020	SD (Type A)	estimated from Figures 5 and 9 using visual scaling method (phyton)
**372**	Tekin, 2020	Diode	IBA Razor diode	MC simulations	Elekta Precise linear accelerator	0.35	Perpendicular	Perpendicular	Force to tip	4 x 4	(k_B,Q)	1.0650	0.0020	SD (Type A)	estimated from Figures 5 and 9 using visual scaling method (phyton)
**373**	Tekin, 2020	Diode	IBA Razor diode	MC simulations	Elekta Precise linear accelerator	1.00	Perpendicular	Perpendicular	Force to tip	4 x 4	(k_B,Q)	1.0700	0.0020	SD (Type A)	estimated from Figures 5 and 9 using visual scaling method (phyton)
**374**	Tekin, 2020	Diode	IBA Razor diode	MC simulations	Elekta Precise linear accelerator	1.50	Perpendicular	Perpendicular	Force to tip	4 x 4	(k_B,Q)	1.0740	0.0020	SD (Type A)	estimated from Figures 5 and 9 using visual scaling method (phyton)
**375**	Tekin, 2020	Diode	IBA Razor diode	MC simulation	Elekta Precise linear	0.35	Perpendicular	Perpendicular	Force to stem	4 x 4	(k_B,Q)	0.9720	0.0020	SD (Type A)	estimated from Figures 5 and 9 using visual scaling method

				s	accelerator										(phyton)
**376**	Tekin, 2020	Diode	IBA Razor diode	MC simulations	Elekta Precise linear accelerator	1.00	Perpendicular	Perpendicular	Force to stem	4 x 4	(k_B,Q)	0.9330	0.0020	SD (Type A)	estimated from Figures 5 and 9 using visual scaling method (phyton)
**377**	Tekin, 2020	Diode	IBA Razor diode	MC simulations	Elekta Precise linear accelerator	1.50	Perpendicular	Perpendicular	Force to stem	4 x 4	(k_B,Q)	0.9310	0.0020	SD (Type A)	estimated from Figures 5 and 9 using visual scaling method (phyton)
**378**	Frick, 2025	Ion Chamber	PTW 31021 Semiflex 3D	Experiments	Elekta Precise linear accelerator	0.35	Perpendicular	Perpendicular	Force to tip	1 x 1	(k_B,Q)	1.0950	0.0150	SD (experimental)	estimated from Figure 5a using visual scaling method (phyton)
**379**	Frick, 2025	Ion Chamber	PTW 31021 Semiflex 3D	Experiments	Elekta Precise linear accelerator	0.35	Perpendicular	Perpendicular	Force to tip	2 x 2	(k_B,Q)	1.0010	0.0090	SD (experimental)	estimated from Figure 5a using visual scaling method (phyton)
**380**	Frick, 2025	Ion Chamber	PTW 31021 Semiflex 3D	Experiments	Elekta Precise linear accelerator	0.35	Perpendicular	Perpendicular	Force to tip	3 x 3	(k_B,Q)	1.0050	0.0080	SD (experimental)	estimated from Figure 5a using visual scaling method (phyton)
**381**	Frick, 2025	Ion Chamber	PTW 31021 Semiflex 3D	Experiments	Elekta Precise linear accelerator	0.35	Perpendicular	Perpendicular	Force to tip	4 x 4	(k_B,Q)	1.0000	0.0070	SD (experimental)	estimated from Figure 5a using visual scaling method (phyton)
**382**	Frick, 2025	Ion Chamber	PTW 31021 Semiflex 3D	Experiments	Elekta Precise linear accelerator	1.50	Perpendicular	Perpendicular	Force to tip	1 x 1	(k_B,Q)	1.0420	0.1500	SD (experimental)	estimated from Figure 5a using visual scaling method (phyton)
**383**	Frick, 2025	Ion Chamber	PTW 31021 Semiflex 3D	Experiments	Elekta Precise linear accelerator	1.50	Perpendicular	Perpendicular	Force to tip	2 x 2	(k_B,Q)	1.0200	0.0090	SD (experimental)	estimated from Figure 5a using visual scaling method (phyton)
**384**	Frick, 2025	Ion Chamber	PTW 31021 Semiflex 3D	Experiments	Elekta Precise linear accelerator	1.50	Perpendicular	Perpendicular	Force to tip	3 x 3	(k_B,Q)	1.0050	0.0080	SD (experimental)	estimated from Figure 5a using visual scaling method (phyton)
**385**	Frick, 2025	Ion Chamber	PTW 31021 Semiflex 3D	Experiments	Elekta Precise linear accelerator	1.50	Perpendicular	Perpendicular	Force to tip	4 x 4	(k_B,Q)	1.0000	0.0070	SD (experimental)	estimated from Figure 5a using visual scaling method (phyton)
**386**	Frick, 2025	Ion Chamber	PTW 31021 Semiflex 3D	Experiments	Elekta Precise linear accelerator	0.35	Perpendicular	Perpendicular	Force to stem	1 x 1	(k_B,Q)	1.0120	0.0120	SD (experimental)	estimated from Figure 5a using visual scaling method (phyton)
**387**	Frick, 2025	Ion Chamber	PTW 31021 Semiflex 3D	Experiments	Elekta Precise linear accelerator	0.35	Perpendicular	Perpendicular	Force to stem	2 x 2	(k_B,Q)	1.0060	0.0090	SD (experimental)	estimated from Figure 5a using visual scaling method (phyton)
**388**	Frick, 2025	Ion Chamber	PTW 31021 Semiflex 3D	Experiments	Elekta Precise linear accelerator	0.35	Perpendicular	Perpendicular	Force to stem	3 x 3	(k_B,Q)	1.0030	0.0080	SD (experimental)	estimated from Figure 5a using visual scaling method (phyton)
**389**	Frick, 2025	Ion Chamber	PTW 31021 Semiflex 3D	Experiments	Elekta Precise linear accelerator	0.35	Perpendicular	Perpendicular	Force to stem	4 x 4	(k_B,Q)	1.0000	0.0070	SD (experimental)	estimated from Figure 5a using visual scaling method (phyton)

**390**	Frick, 2025	Ion Chamber	PTW 31021 Semiflex 3D	Experiments	Elekta Precise linear accelerator	1.50	Perpendicular	Perpendicular	Force to stem	1 x 1	(k_B,Q)	1.1140	0.0140	SD (experimental)	estimated from Figure 5a using visual scaling method (phyton)
**391**	Frick, 2025	Ion Chamber	PTW 31021 Semiflex 3D	Experiments	Elekta Precise linear accelerator	1.50	Perpendicular	Perpendicular	Force to stem	2 x 2	(k_B,Q)	1.0330	0.0110	SD (experimental)	estimated from Figure 5a using visual scaling method (phyton)
**392**	Frick, 2025	Ion Chamber	PTW 31021 Semiflex 3D	Experiments	Elekta Precise linear accelerator	1.50	Perpendicular	Perpendicular	Force to stem	3 x 3	(k_B,Q)	1.0120	0.0090	SD (experimental)	estimated from Figure 5a using visual scaling method (phyton)
**393**	Frick, 2025	Ion Chamber	PTW 31021 Semiflex 3D	Experiments	Elekta Precise linear accelerator	1.50	Perpendicular	Perpendicular	Force to stem	4 x 4	(k_B,Q)	1.0000	0.0070	SD (experimental)	estimated from Figure 5a using visual scaling method (phyton)
**394**	Frick, 2025	Diamond	PTW 60019 microDiamond	Experiments	Elekta Precise linear accelerator	0.35	Parallel	Perpendicular	N/A	1 x 1	(k_B,Q)	0.9620	0.0110	SD (experimental)	estimated from Figure 4b and 4d e using visual scaling method (phyton)
**395**	Frick, 2025	Diamond	PTW 60019 microDiamond	Experiments	Elekta Precise linear accelerator	0.35	Parallel	Perpendicular	N/A	2 x 2	(k_B,Q)	0.9970	0.0080	SD (experimental)	estimated from Figure 4b and 4d e using visual scaling method (phyton)
**396**	Frick, 2025	Diamond	PTW 60019 microDiamond	Experiments	Elekta Precise linear accelerator	0.35	Parallel	Perpendicular	N/A	3 x 3	(k_B,Q)	1.0010	0.0070	SD (experimental)	estimated from Figure 4b and 4d e using visual scaling method (phyton)
**397**	Frick, 2025	Diamond	PTW 60019 microDiamond	Experiments	Elekta Precise linear accelerator	0.35	Parallel	Perpendicular	N/A	4 x 4	(k_B,Q)	1.0020	0.0050	SD (experimental)	estimated from Figure 4d using visual scaling method (phyton)
**398**	Frick, 2025	Diamond	PTW 60019 microDiamond	Experiments	Elekta Precise linear accelerator	1.50	Parallel	Perpendicular	N/A	1 x 1	(k_B,Q)	0.9150	0.0110	SD (experimental)	estimated from Figure 4d using visual scaling method (phyton)
**399**	Frick, 2025	Diamond	PTW 60019 microDiamond	Experiments	Elekta Precise linear accelerator	1.50	Parallel	Perpendicular	N/A	2 x 2	(k_B,Q)	0.9910	0.0080	SD (experimental)	estimated from Figure 4d using visual scaling method (phyton)
**400**	Frick, 2025	Diamond	PTW 60019 microDiamond	Experiments	Elekta Precise linear accelerator	1.50	Parallel	Perpendicular	N/A	3 x 3	(k_B,Q)	1.0020	0.0070	SD (experimental)	estimated from Figure 4d using visual scaling method (phyton)
**401**	Frick, 2025	Diamond	PTW 60019 microDiamond	Experiments	Elekta Precise linear accelerator	1.50	Parallel	Perpendicular	N/A	4 x 4	(k_B,Q)	1.0030	0.0050	SD (experimental)	estimated from Figure 4d using visual scaling method (phyton)
**402**	Yano_2022	Ion Chamber	PTW 31021 Semiflex 3D	MC simulations	Elekta Unity (MC)	1.50	Perpendicular	Parallel	N/A	3 x 3	(FOF)	0.8570	0.0020	SD	Table 4
**403**	Yano_2022	Ion Chamber	PTW 31021 Semiflex 3D	MC simulations	Elekta Unity (MC)	1.50	Perpendicular	Parallel	N/A	2 x 2	(FOF)	0.7870	0.0020	SD	Table 4
**404**	Yano_2022	Ion Chamb	PTW 31021 Semiflex	MC simulation	Elekta Unity (MC)	1.50	Perpendicular	Parallel	N/A	1 x 1	(FOF)	0.5840	0.0010	SD	Table 4

		er	3D	s											
**405**	Yano_2022	Ion Chamber	PTW 31022 PinPoint 3D	MC simulations	Elekta Unity (MC)	1.50	Perpendicular	Parallel	N/A	3 x 3	(FOF)	0.8680	0.0030	SD	Table 4
**406**	Yano_2022	Ion Chamber	PTW 31022 PinPoint 3D	MC simulations	Elekta Unity (MC)	1.50	Perpendicular	Parallel	N/A	2 x 2	(FOF)	0.7940	0.0030	SD	Table 4
**407**	Yano_2022	Ion Chamber	PTW 31022 PinPoint 3D	MC simulations	Elekta Unity (MC)	1.50	Perpendicular	Parallel	N/A	1 x 1	(FOF)	0.6170	0.0030	SD	Table 4
**408**	Yano_2022	Diode	PTW 60018	MC simulations	Elekta Unity (MC)	1.50	Parallel	Perpendicular	force parallel to Photon Fluence	3 x 3	(FOF)	0.8500	0.0020	SD	Table 4
**409**	Yano_2022	Diode	PTW 60018	MC simulations	Elekta Unity (MC)	1.50	Parallel	Perpendicular	force parallel to Photon Fluence	2 x 2	(FOF)	0.7980	0.0020	SD	Table 4
**410**	Yano_2022	Diode	PTW 60018	MC simulations	Elekta Unity (MC)	1.50	Parallel	Perpendicular	force parallel to Photon Fluence	1 x 1	(FOF)	0.6560	0.0010	SD	Table 4
**411**	Das, 2025	Ion Chamber	PTW 31021 Semiflex	Experiments	ViewRay MRIdian	0.35	N/A	Perpendicular	N/A	1 x 1	(FOF)	0.7800	0.0590	SD (est) (experimental)	estimated from Figure 2 using visual scaling method (phyton)
**412**	Das, 2025	Ion Chamber	PTW 31021 Semiflex	Experiments	ViewRay MRIdian	0.35	N/A	Perpendicular	N/A	2 x 2	(FOF)	0.9100	0.0160	SD (est) (experimental)	estimated from Figure 2 using visual scaling method (phyton)
**413**	Das, 2025	Ion Chamber	PTW 31021 Semiflex	Experiments	ViewRay MRIdian	0.35	N/A	Perpendicular	N/A	3 x 3	(FOF)	0.9500	0.0110	SD (est) (experimental)	estimated from Figure 2 using visual scaling method (phyton)
**414**	Das, 2025	Ion Chamber	PTW 31021 Semiflex	Experiments	ViewRay MRIdian	0.35	N/A	Perpendicular	N/A	4 x 4	(FOF)	0.9700	0.0100	SD (est) (experimental)	estimated from Figure 2 using visual scaling method (phyton)
**415**	Das, 2025	Diode	PTW 60023 microSilicon	Experiments	ViewRay MRIdian	0.35	N/A	Perpendicular	N/A	1 x 1	(FOF)	0.8200	0.0620	SD (est) (experimental)	estimated from Figure 2 using visual scaling method (phyton)
**416**	Das, 2025	Diode	PTW 60023 microSilicon	Experiments	ViewRay MRIdian	0.35	N/A	Perpendicular	N/A	2 x 2	(FOF)	0.9150	0.0170	SD (est) (experimental)	estimated from Figure 2 using visual scaling method (phyton)
**417**	Das, 2025	Diode	PTW 60023 microSilicon	Experiments	ViewRay MRIdian	0.35	N/A	Perpendicular	N/A	3 x 3	(FOF)	0.9350	0.0110	SD (est) (experimental)	estimated from Figure 2 using visual scaling method (phyton)
**418**	Das, 2025	Diode	PTW 60023 microSilicon	Experiments	ViewRay MRIdian	0.35	N/A	Perpendicular	N/A	4 x 4	(FOF)	0.9550	0.0100	SD (est) (experimental)	estimated from Figure 2 using visual scaling method (phyton)

**419**	Das, 2025	Diamond	PTW 60019 microDiamond	Experiments	ViewRay MRIdian	0.35	N/A	Perpendicular	N/A	1 x 1	(FOF)	0.8000	0.0600	SD (est) (experimental)	estimated from Figure 2 using visual scaling method (phyton)
**420**	Das, 2025	Diamond	PTW 60019 microDiamond	Experiments	ViewRay MRIdian	0.35	N/A	Perpendicular	N/A	2 x 2	(FOF)	0.9150	0.0170	SD (est) (experimental)	estimated from Figure 2 using visual scaling method (phyton)
**421**	Das, 2025	Diamond	PTW 60019 microDiamond	Experiments	ViewRay MRIdian	0.35	N/A	Perpendicular	N/A	3 x 3	(FOF)	0.9400	0.0110	SD (est) (experimental)	estimated from Figure 2 using visual scaling method (phyton)
**422**	Das, 2025	Diamond	PTW 60019 microDiamond	Experiments	ViewRay MRIdian	0.35	N/A	Perpendicular	N/A	4 x 4	(FOF)	0.9600	0.0100	SD (est) (experimental)	estimated from Figure 2 using visual scaling method (phyton)
**423**	Das, 2025	Scintillator	Standard Imaging PSD W2	Experiments	ViewRay MRIdian	0.35	N/A	Perpendicular	N/A	1 x 1	(FOF)	0.7100	0.0530	SD (est) (experimental)	estimated from Figure 2 using visual scaling method (phyton)
**424**	Das, 2025	Scintillator	Standard Imaging PSD W2	Experiments	ViewRay MRIdian	0.35	N/A	Perpendicular	N/A	2 x 2	(FOF)	0.8700	0.0160	SD (est) (experimental)	estimated from Figure 2 using visual scaling method (phyton)
**425**	Das, 2025	Scintillator	Standard Imaging PSD W2	Experiments	ViewRay MRIdian	0.35	N/A	Perpendicular	N/A	3 x 3	(FOF)	0.9200	0.0110	SD (est) (experimental)	estimated from Figure 2 using visual scaling method (phyton)
**426**	Das, 2025	Scintillator	Standard Imaging PSD W2	Experiments	ViewRay MRIdian	0.35	N/A	Perpendicular	N/A	4 x 4	(FOF)	0.9500	0.0100	SD (est) (experimental)	estimated from Figure 2 using visual scaling method (phyton)
**427**	Das, 2025	Scintillator	Standard Imaging BP-PSD	Experiments	ViewRay MRIdian	0.35	N/A	Perpendicular	N/A	1 x 1	(FOF)	0.7600	0.0570	SD (est) (experimental)	estimated from Figure 2 using visual scaling method (phyton)
**428**	Das, 2025	Scintillator	Standard Imaging BP-PSD	Experiments	ViewRay MRIdian	0.35	N/A	Perpendicular	N/A	2 x 2	(FOF)	0.9200	0.0170	SD (est) (experimental)	estimated from Figure 2 using visual scaling method (phyton)
**429**	Das, 2025	Scintillator	Standard Imaging BP-PSD	Experiments	ViewRay MRIdian	0.35	N/A	Perpendicular	N/A	3 x 3	(FOF)	0.9600	0.0120	SD (est) (experimental)	estimated from Figure 2 using visual scaling method (phyton)
**430**	Das, 2025	Scintillator	Standard Imaging BP-PSD	Experiments	ViewRay MRIdian	0.35	N/A	Perpendicular	N/A	4 x 4	(FOF)	0.9700	0.0100	SD (est) (experimental)	estimated from Figure 2 using visual scaling method (phyton)
**431**	Khan_ 2024	Scintillator	Blue Physics Model 10	MC simulations	ViewRay MRIdian	0.35	Perpendicular	Parallel	N/A	3.32 x 3.32	(k_clin, msr)	1.0070	0.0010	SD (est.)	estimated from Figure 4 using visual scaling method (phyton)
**432**	Khan_ 2024	Scintillator	Blue Physics Model 10	MC simulations	ViewRay MRIdian	0.35	Perpendicular	Parallel	N/A	2.49 x 2.49	(k_clin, msr)	0.9980	0.0020	SD (est.)	estimated from Figure 4 using visual scaling method (phyton)
**433**	Khan_ 2024	Scintillator	Blue Physics	MC simulation	ViewRay MRIdian	0.35	Perpendicular	Parallel	N/A	1.66 x	(k_clin, msr)	0.9960	0.0020	SD (est.)	estimated from Figure 4 using visual scaling method (phyton)

			Model 10	s						1.66					
**434**	Khan_ 2024	Scintill ator	Blue Physics Model 10	MC simulation s	ViewRay MRIdian	0.35	Perpen dicular	Parallel	N/A	0.83 x 0.83	(k_clin, msr)	0.993 0	0.0040	SD (est.)	estimated from Figure 4 using visual scaling method (phyton)
**435**	Khan_ 2024	Scintill ator	Blue Physics Model 10	MC simulation s	ViewRay MRIdian	0.35	Perpen dicular	Parallel	N/A	0.415 x 0.415	(k_clin, msr)	0.990 0	0.0050	SD (est.)	estimated from Figure 4 using visual scaling method (phyton)
**436**	Khan_ 2021	Diode	EDGE Detector	Experimen ts	ViewRay MRIdian	0.35	Parallel	Perpen dicular	N/A	3.3 x 3.3	(FOF)	0.888 0	0.0060	SD (est.)	estimated from Figure 4 using visual scaling method (phyton)
**437**	Khan_ 2021	Diode	EDGE Detector	Experimen ts	ViewRay MRIdian	0.35	Parallel	Perpen dicular	N/A	1.7 x 1.7	(FOF)	0.842 0	0.0060	SD (est.)	estimated from Figure 4 using visual scaling method (phyton)
**438**	Khan_ 2021	Ion Chamb er	N/A	MC simulation s	ViewRay MRIdian	0.35	Parallel	Perpen dicular	N/A	3.3 x 3.3	(FOF)	0.893 0	0.0060	SD (est.)	estimated from Figure 4 using visual scaling method (phyton)
**439**	Khan_ 2021	Ion Chamb er	N/A	MC simulation s	ViewRay MRIdian	0.35	Parallel	Perpen dicular	N/A	1.7 x 1.7	(FOF)	0.854 0	0.0060	SD (est.)	estimated from Figure 4 using visual scaling method (phyton)
**440**	Jelen_ 2020	Ion Chamb er	IBA FC65-G	Experimen ts	Australian MRI-Linac (Prototype)	1.00	Perpen dicular	Perpen dicular	Spiraling	2.6 x 2.6	(FOF)	0.800 0	0.0100	SD (est.)	estimated from Figure 11 using visual scaling method (phyton)
**441**	Jelen_ 2020	Diamon d	PTW 60019 microDiam ond	Experimen ts	Australian MRI-Linac (Prototype)	1.00	Parallel	Parallel	Spiraling	2.6 x 2.6	(FOF)	0.840 0	0.0100	SD (est.)	estimated from Figure 11 using visual scaling method (phyton)

Table S10. Overall pooled mean estimates, 95% confidence intervals (CI), number of data points (k), and heterogeneity (I^2) for Beam Quality Correction Factor (k_{msr}), Field Output Correction Factor (k_{clin}), and Uncorrected Field Output Factor (FOF) across all studies included in the meta-analysis, calculated using a random-effects model.

Dosimetric Quantity	Data Points (k)	Pooled Mean Estimate	95% Confidence Interval	Heterogeneity (I^2)
Beam Quality Corr. Factor (k_{msr})	350	1.006	[0.993, 1.019]	98.2%
Field Output Corr. Factor (k_{clin})	56	1.008	[0.993, 1.023]	92.5%
Field Output Factor (FOF)	35	0.871	[0.838, 0.904]	97.9%

Table S11. Pooled mean estimates, 95% confidence intervals (CI), number of data points (k), and heterogeneity (I^2) for Beam Quality Correction Factor (k_{msr}), Field Output Correction Factor (k_{clin}), and Uncorrected Field Output Factor (FOF), stratified by detector type (Diamond, Diode, Ion Chamber, Plastic Scintillator, TLD).

Dosimetric Quantity	Detector Type	Data Points (k)	Pooled Mean Estimate	95% Confidence Interval	Heterogeneity (I^2)
Beam Quality Corr. Factor (kmsr)	Diamond	51	1.010	[0.981, 1.039]	98.4%
	Diode	63	0.994	[0.975, 1.013]	96.9%
	Ion Chamber	230	1.006	[0.993, 1.019]	97.4%
	Plastic Scintillator	3	1.012	[1.002, 1.022]	0.0%

	TLD	3	1.001	[0.994, 1.008]	0.0%
Field Output Corr. Factor (kclin)	Diamond	3	0.980	[0.954, 1.006]	69.3%
	Diode	6	1.000	[0.982, 1.018]	74.0%
	Ion Chamber	36	1.008	[0.993, 1.023]	91.0%
	Plastic Scintillator	8	0.998	[0.991, 1.005]	62.5%
	TLD	3	1.007	[0.991, 1.023]	62.7%
Field Output Factor (FOF)	Diamond	5	0.871	[0.806, 0.936]	92.4%
	Diode	9	0.862	[0.814, 0.910]	96.9%
	Ion Chamber	13	0.825	[0.760, 0.890]	98.7%
	Plastic Scintillator	8	0.883	[0.824, 0.941]	97.4%

Table S12. Pooled mean estimates, 95% confidence intervals (CI), number of data points (k), and heterogeneity (I^2) for Beam Quality Correction Factor (k_{msr}), stratified by specific detector model.

**Detector Model**	**Data Points (k)**	**Pooled Mean Estimate**	**95% CI**	**I^2 (%)**
PTW 60019 microDiamond	51	1.010	[0.981, 1.039]	98.4
Blue Physics Model 10	9	0.999	[0.994, 1.005]	45.6
PTW 60023 microSilicon	21	1.002	[0.969, 1.035]	98.2
PTW 60012	24	0.988	[0.947, 1.029]	98.1
IBA Razor Diode	15	1.030	[1.010, 1.051]	89.2
PTW 31021 Semiflex 3D	60	1.012	[0.996, 1.027]	97.4

PTW 31022 PinPoint 3D	51	1.011	[0.993, 1.028]	97.8
SNC125c	21	1.001	[0.988, 1.014]	91.8
Exradin A1SL MR	7	1.000	[0.993, 1.006]	55.4
PTW 31010 Semiflex	28	1.033	[0.1003, 1.063]	99.1
IBA Razor Chamber	7	1.000	[0.993, 1.007]	0.0
PTW 31024	6	1.021	[0.988, 1.053]	97.1
Exradin A26 MR	6	0.997	[0.985, 1.009]	78.4
Exradin W2	7	0.998	[0.994, 1.003]	68.2
IBA Razor Nano	6	0.994	[0.987, 1.001]	0.0
PTW 31025	6	1.001	[0.988, 1.014]	80.5
PTW 31016 PinPoint 3D	4	0.967	[0.942, 0.992]	88.5
TLD-MTS 100M	3	1.001	[0.994, 1.008]	0.0

Table S13. Pooled mean estimates, 95% confidence intervals (CI), number of data points (k), and heterogeneity (I^2) for Field Output Correction Factor (k_{clin}), stratified by specific detector model.

Detector Model	Data Points (k)	Pooled Mean Estimate	95% CI	I^2 (%)
Blue Physics Model 10	5	0.997	[0.990, 1.004]	83.2
Exradin A1SL MR	6	1.012	[0.985, 1.039]	91.5
Exradin A26 MR	6	1.002	[0.988, 1.016]	74.3
Exradin W2	3	0.996	[0.984, 1.007]	56.6
IBA Razor Chamber	6	1.005	[0.989, 1.021]	78.4
IBA Razor Diode	3	1.004	[0.998, 1.010]	0.0
IBA Razor Nano	6	0.996	[0.988, 1.004]	59.8
PTW 31024	6	1.020	[0.987, 1.053]	97.0
PTW 31025	6	1.006	[0.989, 1.023]	83.8
PTW 60019 microDiamond	3	0.980	[0.954, 1.006]	69.3
PTW 60023 microSilicon	3	0.987	[0.959, 1.016]	84.4
TLD-MTS 100M	3	1.007	[0.991, 1.023]	62.7

Table S14. Pooled mean estimates, 95% confidence intervals (CI), number of data points (k), and heterogeneity (I^2) for Uncorrected Field Output Factor (FOF), stratified by specific detector model.

Detector Model	Data Points (k)	Pooled Mean Estimate	95% CI	I^2 (%)
PTW 31021 Semiflex 3D	7	0.865	[0.771, 0.960]	99.1
PTW 31022 PinPoint 3D	3	0.760	[0.603, 0.916]	98.6
PTW 60018	3	0.768	[0.651, 0.884]	97.4
PTW 60019 microDiamond	5	0.871	[0.806, 0.936]	92.4
PTW 60023 microSilicon	4	0.906	[0.835, 0.978]	96.6
Blue Physics Model 10	4	0.903	[0.814, 0.991]	96
Exradin W2	4	0.863	[0.751, 0.974]	96.4

Table S15. Pooled mean estimates, 95% confidence intervals (CI), number of data points (k), and heterogeneity (I^2) for Beam Quality Correction Factor (k_{msr}), Field Output Correction Factor (k_{clin}), and Uncorrected Field Output Factor (FOF), stratified by the magnetic field strength (0.35 T, 1.0 T, 1.4 T, 1.5 T) of the MR-Linac system.

Dosimetric Quantity	B-Field (T)	Data Points (k)	Pooled Mean Estimate	95% Confidence Interval	Heterogeneity (I^2)
Beam Quality Corr. Factor (kmsr)	0.35	54	0.998	[0.993, 1.003]	92.5%
	1.0	36	1.004	[0.982, 1.026]	97.4%
	1.4	28	1.009	[0.992, 1.026]	93.3%
	1.5	232	1.009	[0.993, 1.025]	98.6%
Field Output Corr. Factor (kclin)	0.35	8	0.998	[0.991, 1.005]	62.5%
	1.5	48	1.010	[0.993, 1.027]	93.1%
	0.35	20	0.902	[0.864, 0.940]	98.4%

Field Output Factor (FOF)	1.0	2	0.820	[0.795, 0.845]	0.0%
	1.5	13	0.814	[0.748, 0.879]	97.6%

Table S16. Pooled mean estimates, 95% confidence intervals (CI), number of data points (k), and heterogeneity (I^2) for Beam Quality Correction Factor (k_{msr}), Field Output Correction Factor (k_{clin}), and Uncorrected Field Output Factor (FOF), stratified by the specific MR-Linac system used in the studies.

**Dosimetric Quantity**	**MR-Linac System**	**Data Points (k)**	**Pooled Mean Estimate**	**95% Confidence Interval**	**Heterogeneity (I^2)**
Beam Quality Corr. Factor (kmsr)	Elekta Unity	216	1.009	[0.993, 1.024]	98.7%
	Elekta Precise linear accelerator	118	1.003	[0.992, 1.014]	96.6%
	Elekta Synergy	16	0.994	[0.978, 1.010]	89.8%
Field Output Corr. Factor (kclin)	Elekta Unity	51	1.010	[0.993, 1.026]	93.3%
	ViewRay MRIdian	5	0.997	[0.988, 1.006]	69.1%
Field Output Factor (FOF)	Elekta Unity	9	0.768	[0.697, 0.839]	98.1%
	ViewRay MRIdian	24	0.903	[0.865, 0.941]	98.4%
	Australian MRI-Linac	2	0.820	[0.795, 0.845]	0.0%

Table S17. Results of the meta-regression analysis modeling the effect of field size (cm^2) as a continuous variable on Beam Quality Correction Factor (k_{msr}), Field Output Correction Factor (k_{clin}), and Uncorrected Field Output Factor (FOF). The table shows the intercept (value at 0 cm^2), the slope (change per cm^2), and the p-value for the slope for each dosimetric quantity.

**Dosimetric Quantity**	**Intercept (at 0 cm^2)**	**Slope (per cm^2)**	**p-value (for slope)**

Beam Quality Corr. Factor (kmsr)	1.001	+0.0004	0.655
Field Output Corr. Factor (kclin)	1.002	+0.0011	0.518
Field Output Factor (FOF)	0.771	+0.0163	<0.001

Table S18. Pooled mean estimates, 95% confidence intervals (CI), number of data points (k), and heterogeneity (I^2) for Beam Quality Correction Factor (kmsr), stratified by detector orientation relative to the beam and magnetic field, including the direction of the Lorentz force effect where applicable.

**Detector Axis vs. Beam**	**Detector Axis vs. B-Field**	**Lorentz Force Effect**	**Data Points (k)**	**Pooled Mean Estimate**	**95% CI**	**I^2 (%)**
Parallel	Perpendicular	N/A	148	1.012	[0.992, 1.033]	99.0
Perpendicular	Parallel	N/A	96	1.006	[0.993, 1.019]	95.8
Perpendicular	Perpendicular	Force to Stem	48	1.008	[0.993, 1.023]	97.2
Perpendicular	Perpendicular	Force to Tip	48	0.998	[0.957, 1.039]	99.3
Both Orientations	Both Orientations	Both Orientations	3	1.001	[0.994, 1.008]	0.0
Other	Other	Force Parallel to LF	6	1.014	[1.002, 1.027]	86.8

Table S19. Pooled mean estimates, 95% confidence intervals (CI), number of data points (k), and heterogeneity (I^2) for Beam Quality Correction Factor (kmsr), Field Output Correction Factor (kclin), and Uncorrected Field Output Factor (FOF), stratified by the method used to obtain the data.

**Dosimetric Quantity**	**Generation Method**	**Data Points (k)**	**Pooled Mean Estimate**	**95% Confidence Interval**	**Heterogeneity (I^2)**
Beam Quality Corr. Factor (k_{msr})	Experiments	62	1.006	[0.999, 1.013]	94.0%
	MC simulations	288	1.006	[0.992, 1.020]	98.6%
Field Output Corr. Factor (k_{clin})	Experiments	0	-	-	-
	MC simulations	56	1.008	[0.993, 1.023]	92.5%
Field Output Factor (FOF)	Experiments	24	0.890	[0.854, 0.926]	98.4%
	MC simulations	11	0.793	[0.725, 0.861]	97.4%
