# Supplementary material for: Detector‐specific correction factors for small‐field photon dosimetry in magnetic resonance‐guided radiation therapy: A systematic review and meta‐analysis
Source: Med Phys. 2026 Jan 7;53(1):e70201. doi: 10.1002/mp.70201 (PMC12778898; doi:10.1002/mp.70201)
Supplement: Supplementary file 2 — Supporting Information [file MP-53-0-s002.pdf]

7.2 Appendix B: Tables

Table A1. Comprehensive Screening Log for All 86 Included Articles

Study ID	Final Decision	Reason for Decision / Notes
1: Tai_2025	Group 2: For Qualitative Synthesis (Discussion)	This study investigates methods for skin dose reduction. It does not report a core quantitative outcome like a detector-specific correction factor or output factor with uncertainty (Criterion 1.1 & 1.2 not met). It provides valuable methodological guidance (Criterion 2.2).
2: Tai_2022	Group 2: For Qualitative Synthesis (Discussion)	This paper focuses on modeling and measuring skin dose. It does not report detector-specific correction factors required for the meta-analysis (Criterion 1.1 not met). It offers important methodological guidance (Criterion 2.2).
3: Patterson_2022	Group 2: For Qualitative Synthesis (Discussion)	This study characterizes contaminant electrons. It does not provide the core quantitative outcomes with uncertainties required for the meta-analysis (Criterion 1.1 & 1.2 not met). Its value lies in explaining foundational physics (Criterion 2.4).
4: Oliver_2024	Group 2: For Qualitative Synthesis (Discussion)	This is a skin dose investigation that compares Monte Carlo simulations with measurements. It does not report specific, uncertainty-quantified detector correction factors (Criterion 1.1 & 1.2 not met). It provides a valuable comparative analysis (Criterion 2.1).
5: Delfs_2021	Group 1: For Quantitative Synthesis (Meta-Analysis)	Criterion 1.1-1.3 Met: The study reports magnetic field-dependent dose response for a 2.2 x 2.2 cm² field, which meets the small-field criterion. Results are presented numerically with uncertainties, and all methodological context is provided.
6: Shukla_2020	Group 2: For Qualitative Synthesis (Discussion)	This study provides valuable correction factors for a 10x10 cm² reference field. As it does not report data meeting the small-field criterion (≤ 4x4 cm²) for the meta-analysis, it is included for qualitative synthesis due to its important methodological guidance on reference dosimetry (Criterion 2.2).
7: Alissa_2023	Group 2: For Qualitative	The paper determines magnetic field correction factors for a 10x10 cm² reference field. Since it does not contain small-field data, it is excluded from the meta-analysis but

	Synthesis (Discussion)	included for its valuable contribution to the qualitative discussion on reference dosimetry.
8: Mao_2021	Group 2: For Qualitative Synthesis (Discussion)	This Monte Carlo study calculates correction factors for a 10x10 cm² reference field. It does not meet the small-field criterion for the meta-analysis but provides important methodological guidance on MC simulation techniques (Criterion 2.2).
9: Margaroni_2023	Group 1: For Quantitative Synthesis (Meta-Analysis)	Criterion 1.1-1.3 Met: The study provides Monte Carlo calculated correction factors for fields down to 3x3 cm² with uncertainties, meeting all criteria for the small-field meta-analysis.
10: Cervantes_2021	Group 1: For Quantitative Synthesis (Meta-Analysis)	Criterion 1.1-1.3 Met: This study reports quality correction factors for field widths down to 0.25 cm. The data is presented with uncertainties, and all methodological context is provided, making it ideal for the meta-analysis.
11: Cervantes_2020	Group 1: For Quantitative Synthesis (Meta-Analysis)	Criterion 1.1-1.3 Met: The study characterizes chamber response in small fields (e.g., equivalent to 1x1 cm²) and provides numerical data with uncertainties, meeting all criteria.
12: Cervantes_2022	Group 2: For Qualitative Synthesis (Discussion)	This is a Monte Carlo investigation of electron fluence perturbation. It explains foundational physics (Criterion 2.4) but does not report specific correction factors or output factors with uncertainties (Criterion 1.1 & 1.2 not met).
13: Blum_2021	Group 1: For Quantitative Synthesis (Meta-Analysis)	Criterion 1.1-1.3 Met: The study reports the dose response of two solid-state detectors in a 0.59 x 0.59 cm² field. All necessary quantitative data and methodological context are provided.
14: Tekin_2020	Group 1: For Quantitative Synthesis (Meta-Analysis)	Criterion 1.1-1.3 Met: This study investigates detector dose response in a 2.2 x 2.2 cm² field, meeting the small-field criterion. The results are presented numerically with associated uncertainties.
15: Muir_2022	Group 2: For	This work focuses on a prototype ionization chamber for QA. It does not report

	Qualitative Synthesis (Discussion)	standard correction factors for commercial detectors (Criterion 1.1 not met). It provides valuable methodological guidance (Criterion 2.2).
16: Frick_2025	Group 1: For Quantitative Synthesis (Meta-Analysis)	Criterion 1.1-1.3 Met: The paper explicitly determines output correction factors for fields ≤ 3x3 cm². The data is presented with uncertainties in Table 3, meeting all criteria for the meta-analysis.
17: Margaroni_2025	Group 1: For Quantitative Synthesis (Meta-Analysis)	Criterion 1.1-1.3 Met: This Monte Carlo study provides correction factors for fields down to 1x1 cm². Data is presented with uncertainties, and all methodological context is included.
18: Episkopakis_2023	Group 2: For Qualitative Synthesis (Discussion)	This study reports the dose-response of OSLDs in a 10x10 cm² reference field. It does not meet the small-field criterion for the meta-analysis, but provides valuable comparative data on OSLD performance (Criterion 2.1).
19: Khan_2024	Group 2: For Qualitative Synthesis (Discussion)	The paper calculates beam quality correction factors for a 10.5x10.5 cm² field. It is excluded from the meta-analysis due to the large field size but included in the qualitative review for its methodological approach.
20: Renaud_2020	Group 2: For Qualitative Synthesis (Discussion)	This study describes a methodology for absolute dosimetry. It does not report general detector correction factors or small-field output factors with uncertainties (Criterion 1.1 & 1.2 not met).
21: Oolbekkink_N/A	Group 2: For Qualitative Synthesis (Discussion)	This is a performance validation of a novel scintillation dosimeter (Criterion 2.3). It does not provide the standardized quantitative outcomes required for the meta-analysis.
22: Orlando_2025	Group 2: For Qualitative Synthesis (Discussion)	This study reports experimentally measured quality conversion factors for a 10x10 cm² reference field. It does not meet the small-field criterion but is included in the qualitative review for its important experimental data (Criterion 2.1).

23: Andreozzi_2020	Group 2: For Qualitative Synthesis (Discussion)	This paper describes a novel optical imaging method to quantify the ERE. It does not report detector-specific correction factors (Criterion 1.1 not met). It provides excellent methodological guidance (Criterion 2.2).
24: Orlando_N/A	Group 2: For Qualitative Synthesis (Discussion)	This study reports quality conversion factors for 10x10 cm^2 and 10.4x10.4 cm^2 fields. It is excluded from the meta-analysis due to the large field sizes but is valuable for qualitative synthesis due to its extensive experimental data.
25: Steciw_2024	Group 2: For Qualitative Synthesis (Discussion)	This article provides foundational physics insights into the LS-ERE phenomenon (Criterion 2.4) but does not report the core quantitative data required for the meta-analysis.
26: Billas_N/A	Group 2: For Qualitative Synthesis (Discussion)	This study reports correction factors for a 5x5 cm^2 field. As this is outside our pre-defined small-field criterion of ≤ 4x4 cm^2, it is included for qualitative synthesis to discuss dosimetry just outside the small-field regime.
27: Das_2025	Group 1: For Quantitative Synthesis (Meta-Analysis)	Criterion 1.1-1.3 Met: This study directly reports small-field output factors for a 1.1 x 1.1 cm^2 field. The data is presented numerically with associated uncertainties, and all methodological context is provided.
28: Kim_2020	Group 2: For Qualitative Synthesis (Discussion)	This is a surface dosimetry application study. It provides quantitative dose data but does not report detector-specific correction factors or output factors suitable for the meta-analysis (Criterion 1.1 not met).
29: Patterson_2023	Group 2: For Qualitative Synthesis (Discussion)	This work details high-resolution surface dosimetry. While providing important quantitative measurements, it does not report the core correction factors as defined by the protocol (Criterion 1.1 not met).
30: Tyagi_2022	Group 2: For Qualitative Synthesis	This study evaluates the impact of air gaps in QA phantoms. It provides methodological guidance (Criterion 2.2) but does not report detector correction factors.

	(Discussion)	
31: Klavsen_2024	Group 2: For Qualitative Synthesis (Discussion)	The paper focuses on quantifying the uncertainty of a measurement technique itself, which is valuable methodological guidance (Criterion 2.2), but does not report the primary outcome data for meta-analysis.
32: Lim-Reinders_2020	Group 2: For Qualitative Synthesis (Discussion)	This is a surface dose measurement study for IMRT plans. It does not report the core quantitative outcomes (correction factors, output factors) required for the meta-analysis (Criterion 1.1 not met).
33: Gayol_2025	Group 2: For Qualitative Synthesis (Discussion)	This is a methods development paper on a novel Monte Carlo subroutine. It does not report standardized correction factors for commercial detectors (Criterion 1.1 not met). It is a key paper for qualitative synthesis on advanced modeling (Criterion 2.2).
34: Zhang_2021	Group 2: For Qualitative Synthesis (Discussion)	The study assesses out-of-field dose, which is outside the scope of the in-field correction factor meta-analysis. It provides valuable methodological guidance (Criterion 2.2).
35: Chea_2024	Group 2: For Qualitative Synthesis (Discussion)	This is a detector assessment study for commissioning. It provides a comparative analysis of detectors (Criterion 2.1) but does not tabulate specific correction factors with uncertainties.
36: Baines_2021	Group 2: For Qualitative Synthesis (Discussion)	This paper investigates sources of out-of-field dose. It does not report the core in-field quantitative outcomes needed for meta-analysis but is valuable for its comparative analysis (Criterion 2.1).
37: Xhaferllari_2021	Group 2: For Qualitative Synthesis (Discussion)	The focus is on the clinical utility of Gafchromic film, validated with gamma analysis (Criterion 2.3). It does not report detector correction factors with uncertainties.
38: Yano_N/A	Group 1: For Quantitative	Criterion 1.1-1.3 Met: This Monte Carlo study reports calculated output factors for various field sizes, including a 1x1 cm^2 field. The results are presented numerically with

	Synthesis (Meta-Analysis)	associated statistical uncertainties.
39: Etienne_2025	Group 2: For Qualitative Synthesis (Discussion)	This article details the development of an EGSnrc MC model. It provides methodological guidance on MC modeling (Criterion 2.2) but does not report a series of detector-specific correction factors.
40: Shortall_2020	Group 2: For Qualitative Synthesis (Discussion)	This study provides experimental verification of the ERE. It explains foundational physics (Criterion 2.4) but does not report detector-specific correction factors.
41: Boh Lim_2022	Group 2: For Qualitative Synthesis (Discussion)	This paper is an evaluation of EBT-XD film for commissioning using gamma analysis (Criterion 2.3). It does not report specific output factors with associated uncertainties.
42: Crosby_2024	Group 2: For Qualitative Synthesis (Discussion)	This technical note characterizes a novel research platform. It provides methodological guidance (Criterion 2.2) but does not report standardized factors for meta-analysis.
43: van den Dobbelsteen_2022	Group 2: For Qualitative Synthesis (Discussion)	This study provides a dosimetric evaluation of off-axis fields. It provides methodological guidance (Criterion 2.2) but does not provide the core quantitative outcomes required.
44: Sung_2024	Group 2: For Qualitative Synthesis (Discussion)	This paper validates a secondary check software using dose differences and gamma pass rates (Criterion 2.3), not detector correction factors.
45: Ito_2023	Group 2: For Qualitative Synthesis (Discussion)	This article details the development of a dose calculation algorithm. It is a key paper for qualitative synthesis on TPS algorithms (Criterion 2.2) but does not report experimental detector factors.
46: Conrad_2023	Group 2: For	This study investigates the effect of different magnetic field strengths on superficial

	Qualitative Synthesis (Discussion)	dose. It provides a valuable comparative analysis (Criterion 2.1) but does not report the core outcomes for the meta-analysis.
47: Rojas-López_2024	Group 2: For Qualitative Synthesis (Discussion)	This is a commissioning report. While it includes output factors, the data presented in the tables do not include associated uncertainties (SD, SE, CI), thereby failing Criterion 1.2 for meta-analysis.
48: Cusumano_N/A	Group 2: For Qualitative Synthesis (Discussion)	This study characterizes a novel scintillator. It provides a comparative analysis (Criterion 2.1) but does not report factors in a format suitable for meta-analysis.
49: Khan_N/A	Group 1: For Quantitative Synthesis (Meta-Analysis)	Criterion 1.1-1.3 Met: The study reports both computational and experimental small-field output factors for a 1.1 x 1.1 cm^2 field. The results are presented numerically with associated uncertainties.
50: Frick_N/A	Group 2: For Qualitative Synthesis (Discussion)	This study provides a detailed experimental characterization of ionization chambers in a 10x10 cm^2 reference field. It does not meet the small-field criterion for the meta-analysis but offers valuable insights for qualitative synthesis.
51: Wang_2022	Group 2: For Qualitative Synthesis (Discussion)	This is a clinical study comparing treatment plans. It is valuable for its comparative clinical analysis (Criterion 2.1) but does not report detector-specific correction factors.
52: Oolbekkink_2025	Group 2: For Qualitative Synthesis (Discussion)	This paper describes the validation of a specific QA phantom (Criterion 2.3). It does not generate general detector correction factors for meta-analysis.
53: McDonald_2021	Group 2: For Qualitative Synthesis (Discussion)	This is a clinical feasibility study on workflow. It provides guidance on clinical implementation (Criterion 2.2) but not on detector dosimetry.

54: Mittauer_2024	Group 2: For Qualitative Synthesis (Discussion)	This paper details an SRS commissioning process, reporting results as gamma pass rates and positional accuracy, not the specific detector factors required for meta-analysis (Criterion 1.1 & 1.2 not met).
55: van den Dobbelsteen_2023	Group 2: For Qualitative Synthesis (Discussion)	This study is an experimental validation of a clinical process (multi-fraction adaptation). It meets Criterion 2.3 (Performance Validation) but does not report the core quantitative detector factors.
56: Lakomy_2022	Group 2: For Qualitative Synthesis (Discussion)	This R-IDEAL paper reports on clinical implementation and workflow. It does not report detector correction factors, making it suitable for qualitative review.
57: Khan_N/A	Group 1: For Quantitative Synthesis (Meta-Analysis)	Criterion 1.1-1.3 Met: This study reports calculated output factors for a range of field sizes, including a 1.7 x 1.7 cm^2 field. The results are presented with uncertainties implied by comparison to measurement.
58: Yang_N/A	Group 2: For Qualitative Synthesis (Discussion)	This paper describes an independent monitor unit (MU) check. It provides methodological guidance on an essential QA tool (Criterion 2.2) but not on fundamental detector physics.
59: Ruggieri_N/A	Group 2: For Qualitative Synthesis (Discussion)	This study validates a Monte Carlo-based dose check engine. It validates a software tool (Criterion 2.3) but does not report the detector-specific factors required.
60: Shortall_2020	Group 2: For Qualitative Synthesis (Discussion)	This study characterizes dose perturbations around gas cavities, explaining foundational physics (Criterion 2.4) and providing methodological guidance (Criterion 2.2).
61: Iijima_2021	Group 2: For Qualitative Synthesis	This paper describes a novel end-to-end QA phantom. It is valuable for its methodological guidance on QA (Criterion 2.2) but does not report specific detector correction factors.

	(Discussion)	
62: Jelen_2020	Group 1: For Quantitative Synthesis (Meta-Analysis)	Criterion 1.1-1.3 Met: This commissioning paper reports total scatter factors (equivalent to output factors) for a 2.6 x 2.6 cm^2 field. The data points in the relevant figure have error bars, representing the associated uncertainty.
63: Persson_2024	Group 2: For Qualitative Synthesis (Discussion)	This paper validates a dose reconstruction methodology (Criterion 2.3) but does not report the core quantitative detector factors needed for the meta-analysis.
64: Charters_2022	Group 2: For Qualitative Synthesis (Discussion)	This is a dosimetric evaluation of a respiratory gating system, providing a comparative analysis of different techniques (Criterion 2.1) but not the core data for meta-analysis.
65: Lin_2024	Group 2: For Qualitative Synthesis (Discussion)	This paper describes a QA software platform, falling under Criterion 2.3 (Performance Validation) and 2.2 (Methodological Guidance). It does not report the core data for meta-analysis.
66: van den Dobbelsteen_2024	Group 2: For Qualitative Synthesis (Discussion)	This is a performance validation study (Criterion 2.3) of an intrafraction motion correction technique, valuable for the systematic review but not the meta-analysis.
67: Placidi_2020	Group 2: For Qualitative Synthesis (Discussion)	This is a clinical study on an adaptive workflow for pancreatic cancer. It reports on DVH parameters, not detector correction factors, making it suitable for qualitative synthesis (Criterion 2.2).
68: Rusu_2023	Group 2: For Qualitative Synthesis (Discussion)	This clinical study investigates the impact of intrafraction motion, highlighting a key clinical challenge (Criterion 2.2) but not reporting the core quantitative outcomes for the meta-analysis.
69: van Timmeren_2020	Group 2: For Qualitative	This study compares the quality of online-adapted treatment plans, providing a valuable comparative analysis for the systematic review (Criterion 2.1).

	Synthesis (Discussion)	
70: Liu_2025	Group 2: For Qualitative Synthesis (Discussion)	This is a methods-development paper on a DL-based dose calculation engine. It is valuable for its novel methodology (Criterion 2.2) and discussion of future directions (Criterion 2.5).
71: Graham_2022	Group 2: For Qualitative Synthesis (Discussion)	This is a treatment planning study providing a direct comparative analysis of two technologies (Criterion 2.1) but does not report the detector factors required for meta-analysis.
72: Li_2021	Group 2: For Qualitative Synthesis (Discussion)	This study presents a DL-based method for 3D in vivo dose reconstruction. It offers methodological guidance on a novel QA technique (Criterion 2.2) but does not report reference dosimetry data.
73: Lee_2021	Group 2: For Qualitative Synthesis (Discussion)	This is an "in silico" treatment planning study. It provides methodological guidance on an advanced treatment technique (Criterion 2.2) but does not report measured detector data.
74: Slagowski_2020	Group 2: For Qualitative Synthesis (Discussion)	This study compares the dosimetric feasibility of brain SRS between an MR-linac and a conventional linac. It is an excellent example of a comparative analysis (Criterion 2.1).
75: Gupta_2024	Group 2: For Qualitative Synthesis (Discussion)	The focus is on measuring laryngeal motion to inform treatment planning. It provides methodological guidance on motion management (Criterion 2.2) but does not report detector physics data.
76: Adair-Smith_2023	Group 2: For Qualitative Synthesis (Discussion)	This paper evaluates a clinical workflow (radiographer contouring). It is a study on clinical practice (Criterion 2.2) and does not report detector correction factors.
77: Singhrao_2024	Group 2: For	This is a performance validation of an sCT algorithm (Criterion 2.3) and does not report

	Qualitative Synthesis (Discussion)	detector correction factors.
78: Chaknam_2024	Group 2: For Qualitative Synthesis (Discussion)	This study assesses the dosimetric impact of MRI geometric distortion. It provides methodological guidance on an important QA aspect (Criterion 2.2) but not detector correction factors.
79: Kazemifar_2020	Group 2: For Qualitative Synthesis (Discussion)	This is a dosimetric evaluation of sCT for MRI-only proton therapy. It is included in Group 2 for its general methodological relevance to sCT (Criterion 2.3), but the modality difference will be noted.
80: Singhrao_2020	Group 2: For Qualitative Synthesis (Discussion)	This paper describes a novel anthropomorphic phantom for QA. It provides methodological guidance (Criterion 2.2) but does not report general detector correction factors.
81: Galapon_2024	Group 2: For Qualitative Synthesis (Discussion)	This study evaluates uncertainty maps for sCTs for adaptive proton therapy. Included in Group 2 for its general methodological relevance to sCT QA (Criterion 2.2).
82: Kim_2024	Group 2: For Qualitative Synthesis (Discussion)	This is a comparative analysis (Criterion 2.1) and performance validation (Criterion 2.3) of sCT generation methods, not a report on detector correction factors.
83: Lerner_2022	Group 2: For Qualitative Synthesis (Discussion)	This is a prospective clinical feasibility study of an MRI-only workflow. It provides methodological guidance on clinical implementation (Criterion 2.2) but not the core quantitative detector data.
84: Wang_2022	Group 2: For Qualitative Synthesis (Discussion)	This study aims to generate sRPSP images for proton therapy. Included in Group 2 for its methodological guidance on sCT generation and QA (Criterion 2.2).

85: Chourak_2023	Group 2: For Qualitative Synthesis (Discussion)	This paper performs a sensitivity analysis for sCTs, providing methodological guidance (Criterion 2.2), but does not report measured detector correction factors.
86: Hoffmans_2020	Group 2: For Qualitative Synthesis (Discussion)	This is a performance validation study (Criterion 2.3) of an end-to-end test using a deformable phantom and film dosimetry, valuable for the systematic review.
